# ﻿Unlocking Mediterranean bryozoan diversity: seven new species unveiled after fixing a neotype for *Fenestrulina malusii* (Audouin & Savigny, 1826) (Cheilostomatida, Fenestrulinidae)

**DOI:** 10.3897/zookeys.1254.157989

**Published:** 2025-09-30

**Authors:** Antonietta Rosso, Emanuela Di Martino, Gemma Donato, Blanca Figuerola, Vasilis Gerovasileiou, Chiara Siddiolo, Alessandro Sinagra, Rossana Sanfilippo, Francesco Sciuto

**Affiliations:** 1 Department of Biological, Geological and Environmental Sciences, University of Catania, Catania, Italy; 2 CoNISMa – National Inter-universitary Consortium for Sea Sciences, Roma, Italy; 3 Department of Marine Biology and Oceanography, Institute of Marine Science (ICM-CSIC), Pg. Marítim de la Barceloneta 37-49, 08003 Barcelona, Spain; 4 Department of Environment, Faculty of Environment, Ionian University, Zakynthos, Greece; 5 Hellenic Centre for Marine Research (HCMR), Institute of Marine Biology, Biotechnology and Aquaculture (IMBBC), Heraklion, Greece; 6 University School for Advanced Studies IUSS Pavia, Pavia, Italy; 7 Formerly at Department of Biological, Geological and Environmental Sciences, University of Catania, Catania, Italy

**Keywords:** Biodiversity, Bryozoa, genus diagnosis emendation, species complex, taxonomy

## Abstract

*Fenestrulina malusii* (Audouin & Savigny), the type species of *Fenestrulina* Jullien, is among the most widely reported bryozoans globally. Following the loss of the original type material, presumed Mediterranean in origin based on Savigny’s depiction on *Sargassum*, we designate a neotype from a colony on deep *Laminaria* blades off Ustica Island (Tyrrhenian Sea). Additionally, seven new species are described from various habitats across the Mediterranean: *F. cavernicola***sp. nov.**, from the semi-dark and dark zones of a submarine cave in Lesvos Island (NE Aegean Sea); *F. communis***sp. nov.**, from beach-cast plastic near Palermo (NW Sicily); *F. foveolata***sp. nov.**, from Ile-Rousse Bank (Corsica); *F. granulosa***sp. nov.**, from *Posidonia* meadows in Dhiaporia (Chios Island, Aegean Sea); *F. kalliste***sp. nov.**, from outer-shelf coarse sediments off Calvi (NW Corsica); *F. ovata***sp. nov.**, from Trémies submarine cave, near Marseille (Gulf of Lion); and *F. variorugosa***sp. nov.**, from *Posidonia* rootlets off Formica Isle (Egadi Archipelago, near the Sicily Strait). Five of these species are currently known only from their type localities, while *F. communis***sp. nov.** and *F. variorugosa***sp. nov.** occur elsewhere. An additional species with distinct features is described but left in open nomenclature owing to the absence of an ovicell. A comprehensive review of the published images of the 77 species currently assigned to *Fenestrulina*, with the identification of new diagnostic features, including kenozooids, has led to a revised diagnosis of the genus.

## ﻿Introduction

*Fenestrulina malusii* (Audouin & Savigny, 1826) is one of the most frequently reported and apparently widespread extant bryozoan species. The Global Biodiversity Information Facility (GBIF 2024) lists 1,351 occurrences, while the Ocean Biodiversity Information System (OBIS 2024) reports 1,856 presence and 158 absence records spanning the period from 1889 to 2018.

This species was first illustrated by Savigny in 1817 and formally named by Audouin in 1826 as “Cellépore de Malus” or *Cellepora malusii* (Audouin 1826: 239, pl. 8, fig. 8), but no description was provided. It was reported as having been collected during the “Expédition d’Égypte”, but unlike for other species, Audouin (1826) did not specify whether the figured specimens originated from the Mediterranean or the Red Sea. However, Savigny’s (1817) drawings were produced during Napoleon’s Egyptian campaign, which unfolded along the Mediterranean coast (Blondeau and Aubert 1803), making a Mediterranean origin for the material highly probable.

Not long after its introduction, several authors began reporting *F. malusii* from various locations around the world under different generic names, including *Cellepora*, *Lepralia*, and *Microporella*, based on newly collected specimens. However, these identifications appear to have relied solely on Savigny’s drawing, and it remains uncertain whether the type material has ever been examined. Later, *Cellepora malusii* was designated as the type species of the new genus *Fenestrulina*, established by Jullien (1888: 37). He considered the species “cosmopolite” and provided a detailed description (pp. 38–42) of its soft body parts but omitted any information about the mineralised skeleton, stating that the species was too well known to warrant a description of its external form (“trop connue pour que je fasse ici la description de la forme extérieure de sa zoécie”). This suggests that, at least according to Jullien, there was a perceived consensus regarding the species’ identity at the time. However, the literature indicates that both then and later, the concept of *F. malusii* was highly subjective and likely too broad and flexible, accommodating a wide range of intraspecific variability and, consequently, multiple taxonomic entities. For instance, Busk (1857) described the frontal shield of *F. malusii* as being completely covered with pseudopores, which contrasts sharply with the autozooids illustrated by Savigny, where a few pseudopores are confined to the lateral margins. Furthermore, a potentially broader or differing interpretation of the species compared to Jullien’s concept is reflected in the extensive synonymy lists provided by authors such as Harmer (1957), and in the number of taxa still catalogued under the name *F. malusii* in museum collections today (e.g., Wasson and De Blauwe 2014; this study).

Strikingly, despite this taxonomic uncertainty and the absence of reference material, many species have been described in comparison to *F. malusii*, or at least to the prevailing, subjective concept of the species at the time (e.g., Busk 1857; Harmer 1957). Even in recent years, despite increasing recognition of the species’ exclusively Atlanto-Mediterranean distribution (e.g., Hayward and Ryland 1999; Liu et al. 2003), images labelled as *F. malusii* continue to be reported from various global locations, suggesting that *F. malusii* represents a species complex (e.g., Hayward and Thorpe 1989; Souto et al. 2010a) in need of a thorough revision. Misidentifications of colonies have been noted, for instance, by Wasson and De Blauwe (2014), who separated specimens of *F. delicia* Winston, Hayward & Craig, 2000, from material stored as *F. malusii* in the collections of the Natural History Museum, London, UK (NHMUK) and other UK archives.

Focusing on the Atlanto-Mediterranean region, efforts to distinguish morphologically distinct entities has already been undertaken, and some species described from this area in recent decades may result from the ongoing dismantling of the *F. malusii* species complex. Four species have been identified to date, two from the Atlantic European coast and two from the western Mediterranean. In the Atlantic, *Fenestrulina asturiasensis* Álvarez, 1992, is known from a single locality off Spain in the Bay of Biscay (Álvarez 1992), while *F. inesae* Souto, Reverter Gil & Fernandez Pulpeiro, 2010, has been reported from Algarve, off southern Portugal (Souto et al. 2010a). In the Mediterranean, *F. barrosoi* Álvarez, 1993 occurs in the Alboran Sea (Álvarez 1993), and *F. juani* Souto, Reverter Gil & Fernandez Pulpeiro, 2010 off the Balearic Islands (Souto et al. 2010b). Even after the separation of these species, images of *F. malusii* available online or used for comparison in published studies clearly indicate that a plethora of distinct entities continues to be grouped under this species name within the relatively restricted Atlanto-Mediterranean region. This ongoing confusion likely stems from the traditional species concept and the brevity and simplicity of historical descriptions, which focused on a few diagnostic features observable with optical instruments. However, the morphological variability observed today suggests that there is still no clear consensus on the species identity. For instance, colonies from the Aegean Sea documented by Gordon (1984: pl. 41F) differ from those from Spain illustrated by Ramalho (https://www.gbif.org/occurrence/gallery?taxon_key=1008943) and Zabala and Madurell (https://www.gbif.org/occurrence/1227779187), as well as from specimens from France recorded by DORIS (2024). Extremely high variability has also been observed in colonies collected over the past 40 years from several Mediterranean localities by one of us (AR), some of which were previously reported as *F. malusii* in earlier studies (Rosso 1989, 1996a, 1996b; Di Geronimo et al. 1990, 1993, 1997, 1998; Lodolo et al. 2017; Rosso et al. 2019a, 2019b, 2019c). However, Scanning Electron Microscopy (SEM) examination has since revealed that these colonies belong to distinct taxa. This is also evident in additional *F. malusii* specimens recorded from the Mediterranean in studies on bryozoan diversity at both regional and basinal scales (Gautier 1962; Harmelin 1976; Zabala 1986; Zabala and Maluquer 1988; Hayward and McKinney 2002; Chimenz Gusso et al. 2014). In addition, because *F. malusii* was long considered the only Mediterranean species of the easily recognisable genus *Fenestrulina*, it has frequently appeared in ecological studies on specific habitats or geographical areas, often written by non-taxonomists. Occasionally, it has even been cited as an indicator for *Posidonia* meadows (see Discussion). However, all material mentioned in these studies requires revision due to the absence of accompanying figures and descriptions.

In this context, revision and modern redescription of the type material of *F. malusii* are urgently needed to resolve taxonomic uncertainties and to distinguish this species from closely related taxa currently encompassed within its presumed intraspecific variability. However, this effort is hindered by the absence of type specimens. The original material is not listed among the types stored at the Muséum national d’Histoire naturelle in Paris (MNHN) (d’Hondt 2009), and recent attempts to locate it have been unsuccessful. According to d’Hondt (2006: 11), Savigny’s collection was presumably available in Paris until the early 20^th^ century, and subsequently destroyed during World War II bombings. As a result, a neotype must be designated from the type locality. However, uncertainty also surrounds the provenance of the type material. Its supposed Red Sea origin was first mentioned by d’Orbigny (1852: 444), without further clarification and was questioned by Hayward and Ryland (1999). Savigny’s drawing depicts a colony on an algal frond that likely belongs to *Sargassum vulgare* C. Agardh (M. Verlaque and J.-G. Harmelin, pers. comm., 30 July 2024). This species, characterised by its lanceolate fronds, is common in the Mediterranean, and remains unreported from the Red Sea, despite its widespread global distribution (GBIF 2024). In our opinion, and contrary to d’Orbigny’s suggestion, these observations, together with the aforementioned historical context, strongly support a Mediterranean origin for the type material of *F. malusii*, prompting us to seek a Mediterranean colony suitable for designation as the neotype.

The designation of a neotype is a delicate and potentially problematic action, especially when the precise locality and ecological origin of the original material were not specified. This issue has been encountered in the cases of *Parasmittina raigii* (Audouin & Savigny, 1826) and *Microporella ciliata* (Pallas, 1866). For *P. raigii*, Hayward and Parker (1994: figs 6F, 7A, B) arbitrarily selected a neotype from the Red Sea, but doubts remain regarding its conspecificity with the original material. Notably, Savigny’s (1817: pl. 7, fig. 10) drawing depicts a colony with numerous large avicularia, which appear to be absent in the designated neotype. In contrast, similar specimens from the Mediterranean (Lebanon and Port-Cros, France) documented by Harmelin et al. (2009: fig. 4) do exhibit such avicularia. However, while they are clearly adventitious and frontal in the Mediterranean specimens, Savigny’s illustration suggests they may sometimes be interzooidal, especially along the colony margin. A similar issue arises with *M. ciliata*, whose neotype was designated from a specific locality and depth (Punta Palummo Bank in the Bay of Naples, at ~40 m depth) within its broadly reported Mediterranean distribution (Kukliński and Taylor 2008). However, subsequent findings and re-examination of past collections have shown that this morphotype is exceptionally rare in the Mediterranean, documented so far from only two localities (Di Martino and Rosso 2021). In contrast, four other species with similar morphologies are more common and widespread, particularly in shallower and more accessible habitats (Di Martino and Rosso 2021), which are likely the original source of the lost type material. Our initial search for suitable *F. malusii* material focused on the southeastern sector of the Mediterranean, where Napoleonic campaigns took place. However, investigations of bryozoan collections at the NHMUK, the MNHN and The Steinhardt Museum of Natural History, Tel Aviv, Israel (SMNH) yielded no results. Consequently, we face the challenge of selecting a neotype from other Mediterranean regions within our available material. Despite the difficulties posed by the presence of multiple *Fenestrulina* morphotypes in the Mediterranean, we were fortunate to identify colonies that closely match Savigny’s original drawing.

Considering all the above factors, we designated a neotype of *Fenestrulina malusii*, ensuring full compliance with the qualifying conditions set out in Article 75.3 of the International Code of Zoological Nomenclature (ICZN 1999).

The aims of this paper are to designate a neotype for *F. malusii*; describe seven new Mediterranean species of *Fenestrulina* and an additional taxon left in open nomenclature, all resulting from reassessment of the *F. malusii* species complex; update the diagnosis of the genus *Fenestrulina* through comprehensive examination of the studied specimens and a synthesis of information from the literature, covering the vast majority of the 77 species currently assigned to the genus, including those newly described in this study.

## ﻿Materials and methods

**Figure 1.**
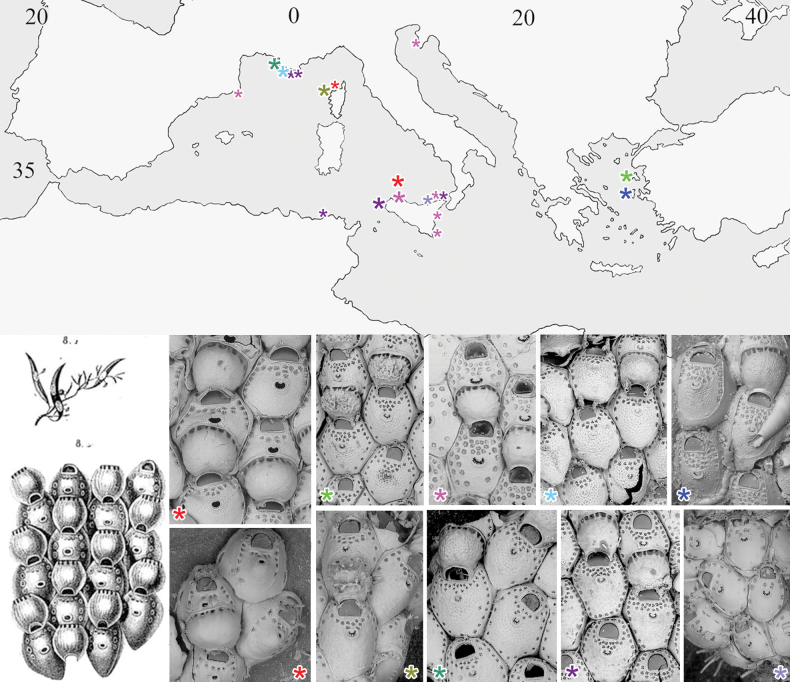
Geographical distribution of Mediterranean *Fenestrulina* species (and specimens) examined in this study. Asterisks denote newly described species and the only confirmed record of *Fenestrulina malusii* (Audouin & Savigny, 1826), illustrated here with Savigny’s original drawing (reproduced from d’Hondt 2006) shown on the left. Type material is shown for each species, except for *F. malusii*, for which the neotype (upper image) and a colony fragment from Ile-Rousse Bank, NW Corsica (lower image) are figured. Large symbols indicate type localities; small symbols mark additional occurrences. Symbol colours correspond to species as follows: red = *F. malusii*; bright green = *F. cavernicola* sp. nov.; vivid pink = *F. communis* sp. nov.; pale blue = *F. foveolata* sp. nov.; blue = *F. granulosa* sp. nov.; olive green = *F. kalliste* sp. nov.; dark green = *F. ovata* sp. nov.; purple = *F. variorugosa* sp. nov.; lilac = *Fenestrulina* sp. Note: in areas with multiple records, only one symbol is used to represent the locality.

This study is mainly based on material collected during the past 40 years through several biodiversity surveys conducted under multiple projects of the University of Catania. It includes also selected colonies housed in the Natural History Museum, London (**NHMUK**) and the Muséum national d’Histoire naturelle in Paris (**MNHN**), as well as specimens and photographs kindly provided by colleagues, particularly J.-G. Harmelin (Station Marine d’Endoume, Marseille, France). The origin of the analysed materials and the type locality of each species are shown in Fig. 1, while sample metadata are summarised in Table 1. Further details on past cruises and sample collections made by the authors, as well as the fauna associated with *Fenestrulina* spp. in those samples, can be found in the following references: CL cruise, off Calvi; R/V Catherine Laurence, Bracors-3 cruise (Rosso 1989; Emig 2018); Apollo Bank, Ustica, southern Tyrrhenian Sea (Di Geronimo et al. 1990); Accademia cave, Ustica, southern Tyrrhenian Sea (Di Geronimo et al. 1997); Egadi Islands (CoNISMa 2009); ST: Ciclopi Islands Marine Protected Area (MPA) (Rosso et al. 2019a); Granchi, Gymnasium and Mazzere caves, Plemmirio MPA (Rosso et al. 2013); PS/81, Piattaforma Siciliana cruise (Rosso 1989, 1996a, 1996b); Agios Vasilios cave, Lesvos Island (Rosso et al. 2019b, 2019c); and off Palamós (Subías-Baratau et al. 2022). Further isolated specimens reported from additional localities, i.e., those from the Graham Bank, Sicily Channel (Di Geronimo et al. 1993; Lodolo et al. 2017), the Amendolara Bank in the northern Ionian Sea (Di Geronimo et al. 1998), and the Antikythera shipwreck, Aegean Sea (Ricci et al. 2019), were not relocated for SEM examination and are, therefore, not included in Table 1 or the synonymy lists.

**Table 1.** List of samples from which the *Fenestrulina* colonies, examined in the present paper, originate. For each sample, information is provided about locality and geographic area, water depth and bionomic assemblage. The number of both living and dead colonies found at the time of sampling is indicated. Abbreviations for samples—AV: Agios Vasilios cave; CL: Calvi; GR: Granchi cave; GY: Gymnasium cave; MZ: Mazzere cave; MI_SdL_G: Milazzo, Secca di Levante scraped; MI_SdPn_G: Milazzo, Secca di Ponente scraped; PS: Piattaforma Siciliana = Sicily shelf; PTT: President Théodore Tissier; ST: Santa Tecla. Abbreviations for biocoenoses — AI: Infralittoral Algae; C: Coralligenous; CB: Bathyal Corals; DC: Coastal Detritic; DL: Offshore Detritic; GO: Dark Caves; GSO: Semidark Caves; HP: *Posidonia* Meadows; SGCF: Coarse Sands and fine Cobbles swept by Bottom Currents; NA: not applicable. Abbreviations for *Fenestrulina* species—Fm: *F. malusii*; Fca: *F. cavernicola* sp. nov.; Fco: *F. communis* sp. nov.; Ff: *F. foveolata* sp. nov.; Fg: *F. granulosa* sp. nov.; Fk: *F. kalliste* sp. nov.; Fo: *F. ovata* sp. nov.; Fv: *F. variorugosa* sp. nov.; Fsp: *Fenestrulina* sp.

Sea	Locality	Sample (Bionomy)	Depth (m)	Living	Dead	Year
Liguro-Provençal	Palamós	Plastic debris (NA)	100	4-Fco	–	2020
	Cassis, Trémies cave	Left chamber (GO)	6	9-Fo	–	1985
		Dark Zone B (GO)	ca 8	6-Fo	–	1982
	Cassis, Calanque Port Miou	JGH-13.06 (C)	17	4-Fo	–	1973
	Cassis, Canyon Cassidaigne	NA (CB)	300	1-Fv	–	1969
	Banyuls-sur-Mer	*5* (CB)	200-300	5-Fv	–	1984
	Veyron Plateau	NA (HP)	24	1-Fv	–	1983
	Ponteau, Gulf of Fos	Pottery (NA)	23	–	1-Fo?	1975
	Port Cros Island	NA, stone (Gravel)	7	1-Fv	–	NA
	Ile-Rousse	PTT, st. 423 (C-*Laminaria*)	85-100	5-Fm; 13-Ff	–	1957
	Calvi	CL 74-12B (DL)	110	–	1-Fk	1983
Tyrrhenian	Egadi Islands	*Posidonia* rhizomes (HP)	8	2-Fv	–	2009
	Apollo Bank (Ustica)	*Laminaria* fronds (C)	60	544-Fm	–	1987
		Sediment (C+DC)	60	1-Fm	11-Fm	
	Accademia cave (Ustica)	Cobbles (C-GSO)	1	–	1-Fco	1989
	Cinisi (Palermo)	Stranded plastic (NA)	NA	16-Fco	–	2023
	Capo Milazzo	MI_SdL_G (C)	33	2-Fv	–	2024
		MI_SdPn_G (C)	33	5-Fv; 1-Fsp.	–	2024
	Tono beach (Milazzo)	Stranded plastic (NA)	NA	2-Fco	–	2024
Ionian	Gulf of Catania	ST.1.Z9 (AI)	9	1-Fco	–	2015
		Ciclopi 20B (SGCF)	50	1-Fco	–	2000
	Plemmirio MPA (Granchi, Gymnasium and Mazzere caves)	GR2 (GSO-GO)	*ca* 20	–	1-Fco	2009-10
		GY2 (GSO-GO)		1-Fco	1-Fco	
		MZ1 (GSO-GO)		–	1-Fco	
	Gulf of Noto	PS/81 CR1 (C+DC)	45	1-Fco	1-Fco	1981
Aegean	Agios Vasilios cave (Lesvos)	AV1 (GSO)	30	2-Fca	1-Fca	2010
		AV2 (GO)	30	1-Fca	–	
	Dhiaporia, off Chios	Unknown (?HP)	? 30-50	1-Fg	–	1967

New, unpublished material was obtained through specialised surveys focused on collecting waste, especially drift plastic, stranded on selected beaches in Sicily to investigate associated fouling communities, and partly conducted in the frame of the PiaCeRi projects awarded to AR and EDM, as well as the doctoral research project of CS (see also Rosso and Siddiolo 2024; Rosso et al. 2025). *Fenestrulina* colonies were found at two locations along the Tyrrhenian coast of Sicily. Colonies were more abundant on a plastic item collected from Magaggiari beach, Cinisi, near Palermo (NW Sicily) in late 2023, and less so on plastic debris from Tono beach, near the tip of the Capo Milazzo Peninsula (NE Sicily), collected in spring 2024. A further few colonies were found in samples collected as part of a study characterising the coralligenous habitat in the Capo Milazzo MPA, conducted within the doctoral research project of GD. Detailed information is provided below for each species.

Scanning electron microscopy (SEM) was performed on uncoated specimens using a TESCAN VEGA 2 LMU in backscattered-electron/low-vacuum mode at the Microscopical Laboratory of the Department of Biological, Geological and Environmental Sciences of the University of Catania.

**Figure 2.**
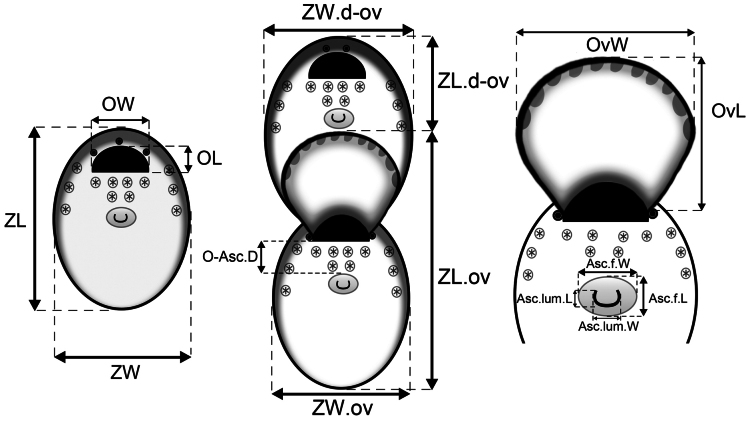
Scheme used for measuring *Fenestrulina* morphological characters. Abbreviations: Asc.f.L and Asc.f.W: ascopore field length and width; Asc.lum.L and Asc.lum.W: ascopore lumen length and width; O.Asc.D: distance between orifice and ascopore; OL and OW: orifice length and width; OvL and OvW: ovicell length and width; ZL and ZW: zooid length and width; ZL.d-ov and ZW.d-ov: length and width of zooids distal to an ovicell; ZL.ov and ZW.ov: length and width of ovicellate zooids.

Measurements (see Fig. 2) were taken from SEM micrographs using the image processing program ImageJ (https://imagej.nih.gov/) and given as ranges, with mean ± standard deviation and, in parentheses, the number of measurements made (*n*). Abbreviations for the measurements of morphological characters are reported in Fig. 2 and Tables 2–4.

**Table 2.** Measurements and qualitative characters for *Fenestrulina malusii*, *F. cavernicola* sp. nov. and *F. communis* sp. nov. (see Fig. 2 for details on the criteria used for measurements and other abbreviations). Anc.L and Anc.W: ancestrula length and width; NA: not applicable; NO: not observed; perianc.: periancestrular; prox.: proximal; d-ov: added when measurements refer to an autozooid located distally to an ovicell.

	Apollo Bank, Ustica	Lesvos Island	Cinisi, Palermo
	*F. malusii* (Audouin & Savigny)	*F. cavernicola* sp. nov.	*F. communis* sp. nov.
ZL	347–535; 421 ± 44 (*n* = 20)	513–525; 518 ± 5 (*n* = 4)	587–715; 646 ± 48 (*n* = 13)
ZW	278–389; 333 ± 32 (*n* = 20)	441–494; 473 ± 23 (*n* = 4)	407–568; 471 ± 38 (*n* = 13)
ZL/ZW	1.26	1.09	1.37
ZL.ov	621–729; 683 ± 42 (*n* = 10)	775–810; 789 ± 18 (*n* = 3)	828–962; 889 ± 43 (*n* = 8)
ZW.ov	262–424; 330 ± 51 (*n* = 10)	419–533; 468 ± 59 (*n* = 3)	401–586; 473 ± 53 (*n* = 8)
ZL.ov/ZW.ov	2.07	1.68	1.88
ZL.d-ov	450–515; 488 ± 20 (*n* = 8)	279–392; 319 ± 43 (*n* = 5)	623–652; 641 ± 19 (*n* = 4)
ZW.d-ov	330–432; 381 ± 30 (*n* = 8)	521–536; 529 ± 11 (*n* = 2)	424–443; 431 ± 8 (*n* = 4)
Pseudopore number	12–20; 16 ± 2 (*n* = 25)	17–35; 26 ± 6 (*n* = 15)	18–40; 26 ± 6 (*n* = 29)
Pseudopore diameter	21–36; 30 ± 4 (*n* = 29)	21–32; 28 ± 3 (*n* = 19)	31–45; 40 ± 4 (*n* = 29)
Pseudopore shape	Semicircular to circular	Tri- to quadrifoliate	Subcircular, infundibular
Pseudopore processes	3–6 radial spiny	3–4 spiny, unjointed	Star-shaped
Pseudopores distribution	Absent proximally	All around or absent proximally	All around or absent proximally
Pseudopore marginal rows	1	1	1–2
Pseudopores rows O-Asc	1–2	1–3	2–3
Pseudopore adjacent to frontal shield rim	Yes	Yes	No
Frontal shield texture	Smooth to finely granular	Dimpled	Smooth
OL	66–86; 77 ± 6 (*n* = 16)	103–118; 111 ± 7 (*n* = 3)	103–136; 123 ± 10 (*n* = 11)
OW	103–133; 118 ± 8 (*n* = 16)	151–159; 155 ± 6 (*n* = 2)	157–188; 176 ± 10 (*n* = 11)
OL/OW	0.65	0.71	0.70
ZL/OL	5.47	4.67	5.25
Orifice distal margin	Undulated to denticulated	Undulated	Fairly denticulated
Orifice proximal margin	2 denticles at corners	2 denticles at corners	Shoulders at corners
Oral spines adult Z	3, occasionally 4	3, rarely 2	2, rarely 3
Oral spines perianc. Z	3–4	2–3	4
Bifurcation prox. pair	Occasionally on perianc. Z	Occasionally compressed	No
Ascopore field shape	Circular-transversely elliptical	Circular-transversely elliptical	Reniform
Ascopore lumen shape	Cordiform to reniform	Transversely C-shaped	Transversely C-shaped
Ascopore rim	Smooth rimmed	Denticulated	Denticulated
Asc.f.L	44–91; 57 ± 10 (*n* = 23)	63–73; 70 ± 3 (*n* = 8)	62–83; 74 ± 6 (*n* = 20)
Asc.f.W	60–140; 83 ± 18 (*n* = 23)	82–97; 87 ± 7 (*n* = 8)	92–138; 113 ± 13 (*n* = 20)
Asc.f.L/Asc.f.W	0.69	0.80	0.65
Asc.lum.L	20–43; 30 ± 8 (*n* = 23)	29–42; 34 ± 4 (*n* = 8)	35–56; 45 ± 5 (*n* = 19)
Asc.lum.W	35–56; 43 ± 6 (*n* = 23)	47–59; 53 ± 5 (*n* = 8)	62–111; 82 ± 12 (*n* = 20)
Asc.f.L.d-ov	45–59; 51 ± 5 (*n* = 8)	51–71; 62 ± 9 (*n* = 4)	65–74; 67 ± 6 (*n* = 4)
Asc.f.W.d-ov	75–98; 85 ± 7 (*n* = 8)	78–93; 84 ± 7 (*n* = 4)	116–131; 124 ± 6 (*n* = 4)
Asc.f.L/Asc.f.W.d-ov	0.60	0.74	0.54
Asc.lum.L-ov	23–38; 32 ± 5 (*n* = 8)	31–38; 34 ± 3 (*n* = 4)	39–48; 43 ± 5 (*n* = 4)
Asc.lum.W-ov	55–71; 64 ± 6 (*n* = 8)	43–56; 51 ± 6 (*n* = 4)	86–101; 93 ± 7 (*n* = 4)
O-Asc.D	57–114; 81 ± 15 (*n* = 34)	74–129; 101 ± 19 (*n* = 17)	107–158; 133 ± 15 (*n* = 23)
OvL	249–350; 318 ± 25 (*n* = 18)	300–324; 312 ± 11 (*n* = 4)	338–401; 359 ± 19 (*n* = 11)
OvW	298–360; 323 ± 15 (*n* = 18)	320–369; 346 ± 22 (*n* = 4)	320–378; 338 ± 16 (*n* = 11)
Ov pore diameter	20–61; 32 ± 9 (*n* = 33)	NA	29–51; 38 ± 6 (*n* = 18)
OvL/OvW	0.98	0.90	1.06
ZL.ov/OvL	2.15	2.53	2.48
Ovicell frontal texture	Smooth to finely granular	With crests and spine-like processes	Smooth to gently nodular
Spines	2, unbranched	2, presumably branched	2, slender unbranched
Ovicell proximal rim	Variably arched	High-arched	Low arched, distal third
Ovicell lateral lappets	Thin, pointed	Long, laterally indented	Not developed
Anc.L	277–342; 304 ± 34 (*n* = 3)	388	436
Anc.W	222–280; 246 ± 30 (*n* = 3)	350	379
Ancestrula spines	10	NO	10

**Table 3.** Measurements and qualitative characters for *Fenestrulina foveolata* sp. nov., *F. granulosa* sp. nov. and *F. kalliste* sp. nov. (see Fig. 2 for details on the criteria used for measurements and other abbreviations). Anc.L and Anc.W: ancestrula length and width; NA: not applicable; NO: not observed; perianc.: periancestrular; prox.: proximal; d-ov: added when measurements refer to an autozooid located distally to an ovicell.

	Ile-Rousse, NW Corsica	Chios	Calvi, NW Corsica
	*F. foveolata* sp. nov.	*F. granulosa* sp. nov.	*F. kalliste* sp. nov.
ZL	354–561; 500 ± 48 (*n* = 14)	475–548; 518 ± 38 (*n* = 3)	420–536; 478 ± 82 (*n* = 2)
ZW	373–483; 436 ± 31 (*n* = 14)	354–428; 387 ± 38 (*n* = 3)	340–439; 390 ± 70 (*n* = 2)
ZL/ZW	1.15	1.34	1.23
ZL.ov	696–832; 790 ± 55 (*n* = 5)	661–856; 759 ± 138 (*n* = 2)	833–984; 913 ± 76 (*n* = 3)
ZW.ov	400–493; 429 ± 40 (*n* = 5)	454	405–520; 455 ± 59 (*n* = 3)
ZL.ov/ZW.ov	1.84	1.67	2.01
ZL.d-ov	324–460; 405 ± 72 (*n* = 3)	238–288; 263 ± 35 (*n* = 2)	278–313; 296 ± 25 (*n* = 2)
ZW.d-ov	427–469; 447 ± 21 (*n* = 3)	424–463; 443 ± 27 (*n* = 2)	425–461; 443 ± 25 (*n* = 2)
Pseudopore number	16–26; 21 ± 3 (*n* = 21)	26–34; 30 ± 3 (*n* = 7)	17–25; 21 ± 3 (*n* = 6)
Pseudopore diameter	21–35; 27 ± 3 (*n* = 21)	20–35; 26 ± 4 (*n* = 22)	22–40; 29 ± 5 (*n* = 20)
Pseudopore shape	Tri- to quadrifoliate	Tri- to quadrifoliate	Irregular, infundibular
Pseudopore processes	1–4 radial spiny, unjointed	3–5 or more, spiny, unjointed	1–4 radial spiny, unjointed
Pseudopores distribution	Absent proximally	Absent proximally	Occasionally proximally
Pseudopore marginal rows	1	1	1
Pseudopores rows O-Asc	1–2	4	1–2
Pseudopore adjacent to frontal shield rim	Yes	Yes	No
Frontal shield texture	Dimpled	Coarsely granular	Smooth to gently nodular
OL	81–105; 94 ± 9 (*n* = 7)	94–102; 99 ± 3 (*n* = 5)	115–138; 127 ± 16 (*n* = 2)
OW	109–133; 123 ± 8 (*n* = 7)	122–152; 138 ± 11 (*n* = 5)	145–169; 157 ± 18 (*n* = 2)
OL/OW	0.76	0.72	0.81
ZL/OL	5.32	5.23	3.76
Orifice distal margin	NO	NO	Smooth
Orifice proximal margin	NO	NO	2 denticles at corners
Oral spines adult Z	3, occasionally 4	1	3
Oral spines perianc. Z	4	NO	3
Bifurcation prox. pair	Yes	NA	Yes
Ascopore field shape	Circular-transversely elliptical	Reniform to cordiform	Circular to elliptical
Ascopore lumen shape	Transversely C-shaped	Transversely C-shaped	Transversely C-shaped
Ascopore rim	Denticulated	Denticulated	Denticulated
Asc.f.L	50–70; 62 ± 7 (*n* = 14)	49–58; 54 ± 4 (*n* = 6)	70–72; 71 ± 1 (*n* = 2)
Asc.f.W	62–91; 78 ± 9 (*n* = 14)	70–78; 74 ± 4 (*n* = 6)	88–91; 90 ± 2 (*n* = 2)
Asc.f.L/Asc.f.W	0.79	0.73	0.79
Asc.lum.L	27–39; 34 ± 4 (*n* = 8)	20–26; 23 ± 3 (*n* = 6)	41–44; 43 ± 2 (*n* = 2)
Asc.lum.W	36–55; 48 ± 6 (*n* = 8)	43–52; 48 ± 3 (*n* = 6)	50–57; 54 ± 5 (*n* = 2)
Asc.f.L.d-ov	46–52; 49 ± 3 (*n* = 4)	39–41; 40 ± 1 (*n* = 2)	61–76; 68 ± 7 (*n* = 3)
Asc.f.W.d-ov	56–86; 67 ± 13 (*n* = 4)	78–88; 83 ± 7 (*n* = 2)	80–93; 86 ± 6 (*n* = 3)
Asc.f.L/Asc.f.W.d-ov	0.73	0.48	0.79
Asc.lum.L-ov	28–34; 30 ± 3 (*n* = 4)	21–22; 22 ± 1 (*n* = 2)	32–33; 33 ± 1 (*n* = 3)
Asc.lum.W-ov	40–48; 44 ± 3 (*n* = 4)	58–60; 59 ± 1 (*n* = 2)	47–63; 55 ± 8 (*n* = 3)
O-Asc.D	80–118; 106 ± 9 (*n* = 17)	119–171; 134 ± 17 (*n* = 8)	81–109; 94 ± 10 (*n* = 5)
OvL	300–347; 315 ± 19 (*n* = 5)	367	346–385; 369 ± 20 (*n* = 3)
OvW	318–348; 334 ± 10 (*n* = 6)	327	310–356; 326 ± 26 (*n* = 3)
Ov pore diameter	15–26; 22 ± 3 (*n* = 12)	18–81; 36 ± 15 (*n* = 15)	16–28; 20 ± 5 (*n* = 10)
OvL/OvW	0.94	1.12	1.13
ZL.ov/OvL	2.51	2.07	2.47
Ovicell frontal texture	Dimpled	Granular	Tuberculate to rugose
Spines	2, branched	None	2, branched
Ovicell proximal rim	Highly arched	Tubular, arched	Arched, distal third
Ovicell lateral lappets	Short, not indented	Not developed	Not developed
Anc.L	387	NO	NO
Anc.W	282	NO	NO
Ancestrula spines	10	NO	NO

**Table 4.** Measurements and qualitative characters for *Fenestrulina ovata* sp. nov., *F. variorugosa* sp. nov. and *Fenestrulina* sp. (see Fig. 2 for details on the criteria used for measurements and other abbreviations). Anc.L and Anc.W: ancestrula length and width; NA: not applicable; NO: not observed; perianc.: periancestrular; prox.: proximal; d-ov: added when measurements refer to an autozooid located distally to an ovicell.

	Trémies cave, Cassis	Egadi Islands	Milazzo Peninsula
	*F. ovata* sp. nov.	*F. variorugosa* sp. nov.	*Fenestrulina* sp.
ZL	518–639; 582 ± 41 (*n* = 13)	515–646; 599 ± 42 (*n* = 16)	359–473; 408 ± 38 (*n* = 10)
ZW	391–538; 470 ± 48 (*n* = 13)	379–525; 436 ± 53 (*n* = 16)	282–431; 315 ± 51 (*n* = 10)
ZL/ZW	1.24	1.37	1.29
ZL.ov	766–878; 838 ± 52 (*n* = 4)	860–917; 883 ± 30 (*n* = 3)	NO
ZW.ov	377–507; 449 ± 54 (*n* = 4)	444–565; 451 ± 12 (*n* = 3)	NO
ZL.ov/ZW.ov	1.87	1.96	NA
ZL.d-ov	410–554; 461 ± 80 (*n* = 3)	313–381; 352 ± 33 (*n* = 4)	NO
ZW.d-ov	398–459; 421 ± 33 (*n* = 3)	372–435; 395 ± 28 (*n* = 4)	NO
Pseudopore number	25–44; 34 ± 6 (*n* = 14)	32–45; 39 ± 4 (*n* = 18)	7–23; 13 ± 5 (*n* = 10)
Pseudopore diameter	18–27; 22 ± 3 (*n* = 20)	24–38; 32 ± 4 (*n* = 20)	18–37; 24 ± 4 (*n* = 34)
Pseudopore shape	Subcircular to trifoliate	Subcircular to lobate	Subcircular, infundibular
Pseudopore processes	2–4 spiny, unjointed	3–4 radial spiny	2–4 spiny, jointed
Pseudopores distribution	All around or absent proximally	All around or absent proximally	All around or absent proximally
Pseudopore marginal rows	1–2	1	1
Pseudopores rows O-Asc	1–3	1–3	1–2
Pseudopore adjacent to frontal shield rim	Yes	Yes, in some autozooids	Yes, in a few autozooids
Frontal shield texture	Gently dimpled centrally	Smooth to nodular	Irregularly smooth
OL	114–127; 120 ± 4 (*n* = 13)	112–137; 128 ± 7 (*n* = 20)	87–103; 91 ± 5 (*n* = 7)
OW	139–163; 152 ± 8 (*n* = 13)	144–170; 158 ± 7 (*n* = 20)	102–124; 112 ± 9 (*n* = 7)
OL/OW	0.79	0.81	0.81
ZL/OL	4.85	4.67	4.48
Orifice distal margin	Smooth	Fairly denticulated	Fairly undulated
Orifice proximal margin	Low shoulders at corners	2 denticles at corners	NO
Oral spines adult Z	3 distal, occasionally 1–2	2, very distal, rarely 3	4, occasionally 3
Oral spines perianc. Z	NO	4	NO
Bifurcation prox. pair	No	No	Yes
Ascopore field shape	Circular-transversely elliptical	Reniform	Reniform
Ascopore lumen shape	Transversely C-shaped	Transversely C-shaped	Transversely C-shaped
Ascopore rim	Denticulated	Denticulated	Denticulated
Asc.f.L	56–78; 66 ± 6 (*n* = 11)	56–68; 63 ± 4 (*n* = 6)	39–54; 48 ± 5 (*n* = 11)
Asc.f.W	79–96; 86 ± 5 (*n* = 11)	88–99; 92 ± 4 (*n* = 6)	49–74; 66 ± 8 (*n* = 11)
Asc.f.L/Asc.f.W	0.77	0.68	0.72
Asc.lum.L	28–40; 34 ± 5 (*n* = 10)	36–46; 39 ± 4 (*n* = 6)	26–33; 29 ± 2 (*n* = 11)
Asc.lum.W	46–62; 54 ± 4 (*n* = 10)	64–72; 68 ± 3 (*n* = 6)	36–61; 50 ± 8 (*n* = 11)
Asc.f.L.d-ov	40–67; 54 ± 19 (*n* = 2)	57–63; 60 ± 3 (*n* = 4)	NO
Asc.f.W.d-ov	84–91; 88 ± 5 (*n* = 2)	80–91; 86 ± 4 (*n* = 4)	NO
Asc.f.L/Asc.f.W.d-ov	0.61	0.70	NA
Asc.lum.L-ov	27–30; 29 ± 2 (*n* = 2)	34–38; 37 ± 2 (*n* = 4)	NO
Asc.lum.W-ov	53–60; 57 ± 5 (*n* = 2)	59–68; 66 ± 4 (*n* = 4)	NO
O-Asc.D	84–126; 102 ± 11 (*n* = 20)	70–109; 89 ± 11 (*n* = 23)	62–108; 74 ± 12 (*n* = 13)
OvL	371–432; 404 ± 28 (*n* = 5)	387–422; 406 ± 17 (*n* = 5)	NO
OvW	373–417; 389 ± 17 (*n* = 5)	347–368; 353 ± 8 (*n* = 5)	NO
Ov pore diameter	12–40; 28 ± 8 (*n* = 25)	19–34; 26 ± 4 (*n* = 14)	NO
OvL/OvW	1.04	1.15	NA
ZL.ov/OvL	2.07	2.17	NA
Ovicell frontal texture	Dimpled	Gently nodular to radially wrinkled	NO
Spines	2, slender, hidden	2, unbranched	NO
Ovicell proximal rim	Low arched	High-arched	NO
Ovicell lateral lappets	Ovate to proximal orifice	Long, laterally indented	NO
Anc.L	NO	305	NO
Anc.W	NO	220	NO
Ancestrula spines	NO	10	NO

Note that we attribute the authorship of *F. malusii* to Audouin & Savigny, 1826, consistent with our approach to *Microporella* species (Di Martino and Rosso 2021). Type and figured specimens are part of Rosso, Harmelin, and Di Martino-Figuerola collections housed at the Museum of Palaeontology of the University of Catania (**PMC**), under the catalogue numbers specified for each species, except for those specimens belonging to the NHMUK and the MNHN collections. A. Rosso and E. Di Martino were responsible for the systematic section of this paper and are to be considered the taxonomic authors for the newly described species.

## ﻿Taxonomic treatment

### ﻿Phylum Bryozoa Ehrenberg, 1831

**Class Gymnolaemata Allman, 1856**

**Order Cheilostomatida Busk, 1852**

**Superfamily Schizoporelloidea Jullien, 1883**

**Family Fenestrulinidae Jullien, 1888**

#### ﻿Genus Fenestrulina

Taxon classificationAnimaliaCheilostomatidaFenestrulinidae

Jullien, 1888

B231FDAC-21B3-5997-A404-F44DA13747B3

##### Type species.

*Cellepora malusii* Audouin & Savigny, 1826.

#### ﻿ Fenestrulina malusii

Taxon classificationAnimaliaCheilostomatidaFenestrulinidae

(Audouin & Savigny, 1826)

4D64D8B5-5058-5F2D-913D-9F95DEB374CB

Figs 1, 3, 4, 5, 6, 22, 23, 24; Tables 1, 2

Fenestrulina malusii Audouin 1826: 239, pl. 8, fig. 8.
Fenestrulina malusii Audouin: Di Geronimo et al. 1988; 1990: table 1; Rosso 1989: tables 3d, 4d.

##### Type material.

Italy • ***Neotype*** 1 ovicellate colony, including the regenerated ancestrula and more than 100 autozooids. On fronds of *Laminaria rodriguezii* Bornet, Mediterranean, Tyrrhenian Sea, southwest of Ustica Island, Apollo Bank; 38°7'N, 13°1'E; 60 m depth; Jun. 1986; I. Di Geronimo leg.; scuba diving; PMC.B37.Neotype 23.2.2024.

##### Other examined material.

Italy • 18 small colonies, each including tens of autozooids, with ancestrula and ovicells, except for one only consisting of a large lobe of ~30 autozooids; same details as the neotype; PMC Rosso-Collection I.H.B.115.a. Italy • Additional 525 ovicellate and non-ovicellate colonies, more poorly preserved than previous material; same details as the neotype; PMC Rosso-Collection I.H.B.115.b. France • 5 fragmentary colonies still attached to their substrate and some isolated detached autozooids including one with opposite regeneration. On fronds of *L. rodriguezii*, Mediterranean, Liguro-Provençal basin, NW Corsica, Ile-Rousse Bank; coordinates not available; 85–100 m depth; 5 Aug. 1957; R/V Président Théodore Tissier survey, St. 423; J.-G. Harmelin leg.; PMC. Harmelin-Collection F.H.B.115.c.

##### Diagnosis.

*Fenestrulina* with smooth-rimmed, roundish ascopore, a simple distal process, a wide lumen centrally positioned within a markedly convex, smooth to finely granular frontal shield bordered by a row of small marginal pseudopores not extending proximally; ovicell smooth.

##### Description.

**Figure 3.**
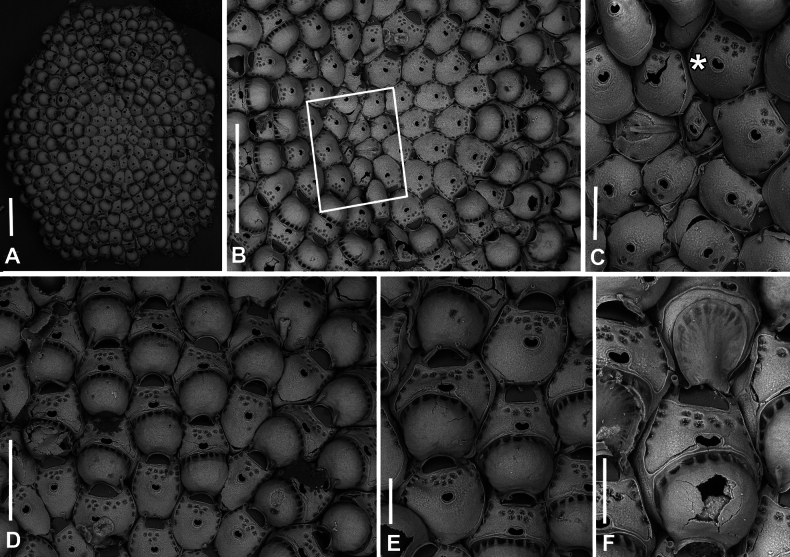
*Fenestrulina malusii* (Audouin & Savigny, 1826) neotype PMC.B37.Neotype.23.2.2024.a, Apollo Bank, Ustica Island, Tyrrhenian Sea. **A.** General view of the colony, largely composed of ovicellate autozooids; **B.** Central zone with non-ovicellate autozooids, encircled by rings of ovicellate ones; **C.** Enlargement of the framed area in **B**, showing the ancestrula regenerated as a miniature autozooid, and surrounding periancestrular autozooids, one of which is ovicellate (asterisk); **D.** Colony periphery, almost entirely composed of ovicellate autozooids; **E.** A single non-ovicellate autozooid surrounded by ovicellate ones. Note the reniform, smooth ascopore with a tiny distal process; **F.** Developing ovicell. Scale bars: 1 mm (**A, B**); 200 μm (**C, E, F**); 500 μm (**D**).

**Figure 4.**
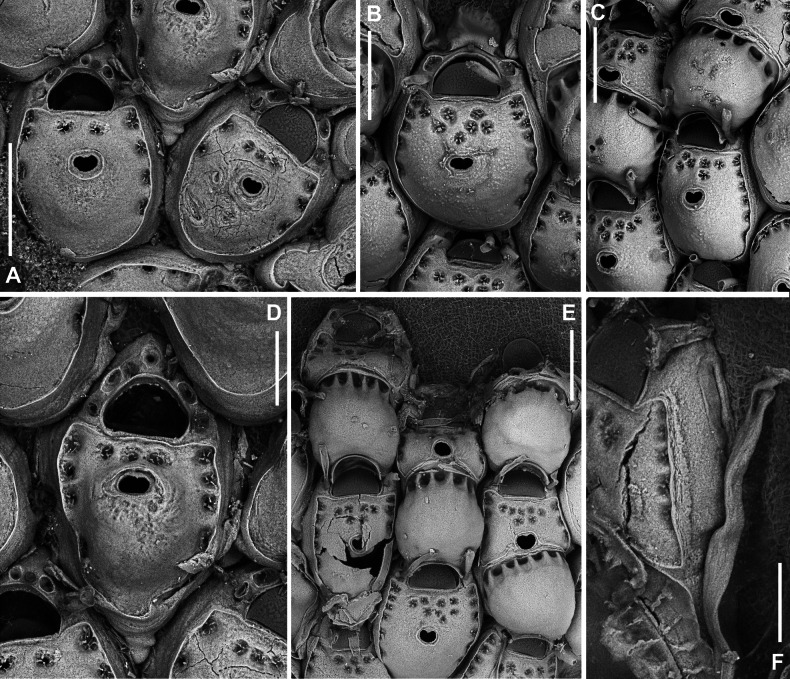
*Fenestrulina malusii* (Audouin & Savigny, 1826) Apollo Bank, Ustica Island, Tyrrhenian Sea. **A, D.**PMC. Rosso Coll. I.H.B.115.a2; **B, C, F.**PMC. Rosso Coll. I.H.B.115.a3; **E.**PMC. Rosso Coll. I.H.B.115.a5. **A.** Non-ovicellate autozooids from the colony centre, showing few pseudopores; **B.** Non-ovicellate autozooid from the colony periphery, with more numerous pseudopores; **C.** Inclined ovicellate autozooids showing the proximal constriction of the ovicell and its upward-folding rim; **D.** Autozooid showing the orifice with a finely and irregularly denticulate distal margin and a straight proximal margin bearing two tiny denticles. Note the substrate exposed between the loosely spaced autozooids; **E.** Colony margin with developing autozooids; **F.** Fissure-like pore chambers along the distolateral wall of an autozooid. In panels **B, C**, and **E** note the long, weakly calcified oral spines. Scale bars: 200 μm (**A–C, E**); 100 μm (**D, F**).

**Figure 5.**
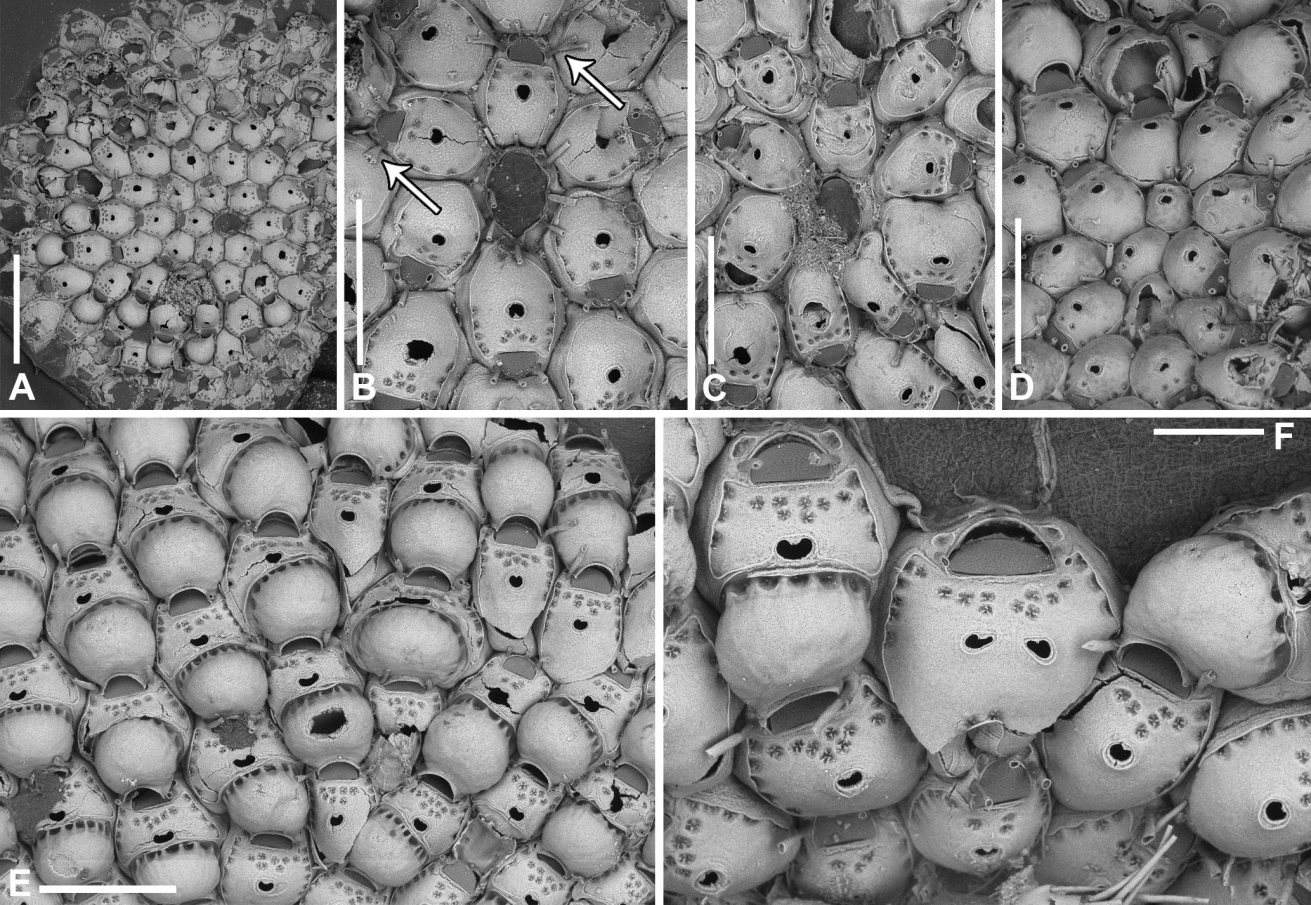
*Fenestrulina malusii* (Audouin & Savigny, 1826) Apollo Bank, Ustica Island, Tyrrhenian Sea. **A, B.**PMC. Rosso Coll. I. H. B.115a1; **C.**PMC. Rosso Collection I. H. B.115a2; **D.**PMC. Rosso Coll. I.H.B.115.a6; **E.** neotype PMC.B37.Neotype.23.2.2024; **F.**PMC. Rosso Coll. I. H. B.115.a4. **A.** Colony showing a ring of ovicellate autozooids surrounding the first generations of non-ovicellate autozooids near the ancestrula; **B.** Detail of the ancestrula and six periancestrular autozooids in panel **A**. Arrows indicate bifurcated spines; **C.** Tatiform ancestrula surrounded by seven irregularly budded and partly regenerated autozooids; **D.** Ancestrula regenerated as a miniature autozooid. Note the early development of ovicells; **E.** Zone of ovicellate autozooids with only three non-ovicellate ones, showing shape variability, and an abnormal autozooid producing a large ovicell; **F.** Large teratological autozooid with a wide orifice and two drop-shaped ascopores, resulting from the fusion of two contiguous autozooids, as indicated by the morphology of the proximal margin. Scale bars: 1 mm (**A**); 500 μm (**B–E**); 200 μm (**F**).

**Figure 6.**
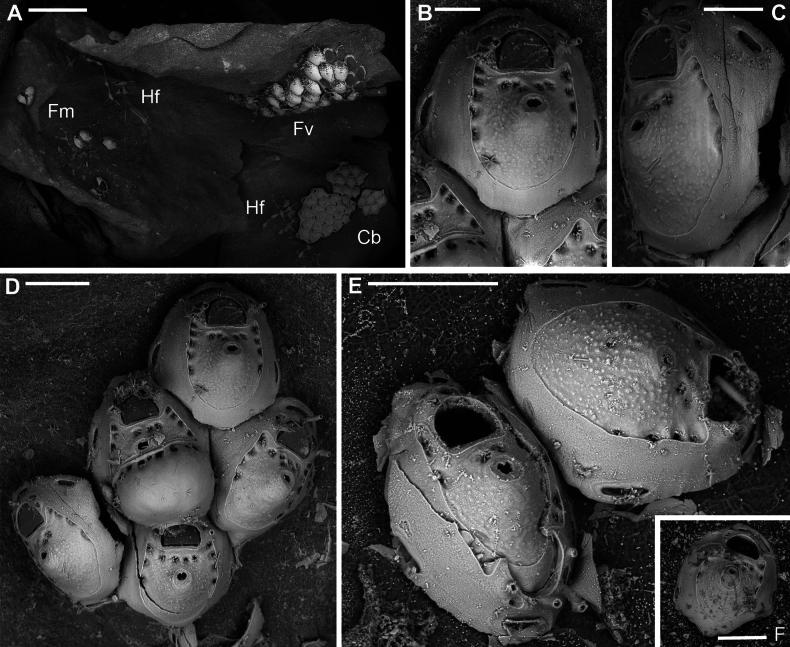
*Fenestrulina malusii* (Audouin & Savigny, 1826) PMC Harmelin Coll. I.H.B.115.c, Ile-Rousse Bank, NW Corsica, Liguro-Provençal basin. **A.** Fragment of a *Laminaria* frond with some autozooids (two enlarged in panel **E**) still attached, indicating the remnants of a once larger colony. Note the co-occurrence of *F. variorugosa* sp. nov. and other bryozoans: Cb = *Chorizopora brongniartii* (Audouin & Savigny, 1826); Fm = *F. malusii*; Fv = *F. variorugosa* sp. nov.; Hf = *Hippothoa flagellum* Manzoni, 1870; **B, C.** Close-ups of two autozooids from the colony fragment in panel **D**, showing elongate pore chambers along distal and distolateral walls; **D.** Group of autozooid, including one ovicellate; **E.** Two autozooid from the colony in panel **A**, one regenerated with reversed polarity; **F.** Isolated detached autozooid with a regenerated ascopore zone. Scale bars: 1 mm (**A**); 100 μm (**B, C**); 200 μm (**D–F**).

Colony encrusting algal fronds, multiserial, unilaminar, typically subcircular to slightly subelliptical (Figs 3A, 5A), rarely lobate, up to ~7 mm in maximum dimension; initially consisting of concentric generations of alternating zooids, later becoming progressively irregular; interzooidal communications via pore-chambers: two proximolateral, two distolateral, one distal (~180 μm long) near base of vertical walls; pore-chamber windows fissure-like and barely visible (Fig. 4F) or subelliptical (Fig. 6B–E), usually masked by developing autozooids, even at colony periphery.

Autozooids ovoidal to rounded hexagonal, distinct, boundaries marked by narrow, deep grooves widening into subtriangular spaces at triple junctions (Figs 3D–F, 4, 5B–D, 6). Lateral and proximal walls well exposed (50–70 μm wide), deeply sloping, in contact with neighbouring zooids only near base (Figs 4, 6). Cryptocystidean frontal area bordered by a thin, raised rim of smooth calcification, typically straight distally just proximal to orifice (Fig. 4A, C, D), or with paired, short (~30 μm) lateral extensions (Figs 4A–D, 5F, 6B–D). Frontal shield convex, most elevated at ascopore level, smooth to finely, densely and evenly granular; perforated by fissure-like, semicircular to circular pseudopores, mainly adjacent to the edge of the slightly higher gymnocystal rim (Figs 3C, E, F, 4, 6B–E, 23A), confined to the distal half to two-thirds of autozooid, numbering 12–20 (8–10 in periancestrular autozooids) (Fig. 5); area between orifice and ascopore with 5–10 additional pseudopores (2 or 3 in periancestrular autozooids). Pseudopores with 3–6 (usually 5) radial spiny processes centrally unjointed (Figs 4B, C, 5F, 23A). Two, rarely three, cryptocystidean areas with simple pores distal to orifice, interspersed among spines, soon concealed by distal autozooids (often covering oral spines as well), visible only at colony margin or in disjointed autozooids (Fig. 4D). Basal wall nearly uncalcified, except for a thin peripheral ring near vertical walls.

Primary orifice transversely D-shaped, hinge-line straight, with two minute denticles near proximal corners; distal rim slightly undulating to distinctly denticulated (Fig. 4D). Three, occasionally four, slender, weakly calcified tubular oral spines, up to ~130 μm long (base diameter 15–20 μm); proximalmost pair larger, positioned at mid-orifice length. Periancestrular autozooids usually with four oral spines, proximalmost pair occasionally bifurcated (Fig. 5B, arrowed). In ovicellate zooids, spines reduced to two, always visible, adjacent to and often indenting lateral ovicell margin (Figs 3D–F, 4C, E, 5D–F, 6D).

Ascopore centrally placed, ~80 μm proximal to orifice (Figs 3E, 4, 5), with a smooth rimmed subcircular, heart-shaped to transversely reniform lumen, featuring a simple to bifurcated distal process, occasionally subcircular (Fig. 5B); situated within a circular to transversely elliptical narrow field of smooth gymnocystal calcification, marked by a raised rim, laterally merging with the arched proximal rim of frontal shield in ovicellate zooids (Fig. 3E, F).

Ovicell subglobular, prominent, partially obscuring the distal part of the orifice, seemingly subcleithral and only partly closed by the operculum, produced by the distal autozooid (Fig. 3D, E). Endooecium calcified, smooth, rimmed by a row of 14–17 large, quadrangular pores separated by calcified ribs, creating a scalloped distal margin; narrowing proximally, with proximal rim folded upward into a thin protruding visor and extending into pointed lateral wings (Figs 4C, E, 5D–F). Ectooecium mainly cuticular with a slightly raised rim of gymnocystal calcification along the proximal raised edge of the distal autozooid (Fig. 3F).

Ancestrula tatiform (Figs 5A–C, 24A), oval, slightly smaller than periancestrular autozooids; gymnocyst more extensive proximally (~80 μm wide), tapering distally, rim sometimes undulating between 10 gymnocystal spines (five distal, more closely spaced than the equally spaced proximal ones). Cryptocystidean areas with simple pseudopores lateral to the distal triplet of spines, barely detectable. Ancestrula sometimes regenerating as miniature autozooids (Fig. 3C) or resembling a miniature autozooid without clear signs of regeneration (Fig. 5D). Budding pattern: one distal, two distolateral, two proximolateral, and one or two proximal zooids, totalling six or seven periancestrular autozooids, sometimes ovicellate (Fig. 3C).

Kenozooids not observed.

##### Remarks.

A notable character of *F. malusii* is the ascopore with a smooth rim and a wide cordiform to reniform lumen, unique among all species examined from the Mediterranean. A smooth-rimmed ascopore is clearly depicted in Savigny’s drawing of *F. malusii* (Fig. 1). When properly oriented, frontal views of ovicellate regions also reveal the same characters well illustrated in Savigny’s drawings, including the scarcity of pseudopores and their location, and the distal pores of the ovicell. These traits are particularly distinctive in the Mediterranean material, leading to the selection of this *Fenestrulina* population, and the best preserved colony within it, as the neotype for the species, in the absence of colonies from the original collection (see Introduction). A smooth-rimmed ascopore has been reported in some species from the Southern Hemisphere, such as *F. cervicornis* Hayward & Ryland, 1990 from the Ross Sea (Antarctica), *F. fritilla* Hayward & Ryland, 1990 and *F. jocunda* Hayward & Ryland, 1990, both from South Georgia and the former species also from Burdwood Bank (subantarctic region), and *F. microstoma* Moyano, 1983 from off the Chilean coast, north of Concepción, in the Pacific Ocean. In *F. cervicornis*, *F. fritilla* and *F. microstoma*, however, the lumen is subelliptical or slightly crescentic, while in *F. jocunda*, it is slit-like and mounted on an elevation (Moyano 1983; Hayward and Ryland 1990; Hayward 1995). All these species strongly differ from *F. malusii* in several characters, such as the bifurcation or trifurcation of the proximalmost pair of oral spines in *F. cervicornis*, the absence of oral spines and the entirely pseudoporous frontal shield in *F. fritilla*, the large cribriform pseudopores of the frontal shield and the deeply pitted, wrinkled appearance of the ovicell endooecium in *F. jocunda*, and the almost entirely perforated frontal shield in *F. microstoma* (Moyano 1983; Hayward and Ryland 1990; Hayward 1995). Broadly non-denticulate, but distinctly different, thin, slit-like C-shaped ascopores occur in *F. thyreophora* (Busk, 1857), a widespread, presumed highly variable southern hemisphere species.

Although roughly smooth surfaced, autozooids of *F. malusii* often show some granules, recalling those of *Fenestrulina* sp., a still unnamed species from Safaga Bay in the Red Sea (Ostrovsky et al. 2024; https://bryozoancollection.univie.ac.at/Sammlung/Bryozoa/Safaga_Bay/Cheilostomata/Microporellidae/Fenestrulina/Fenestrulina_sp.html). However, in that species, the granules are fewer and larger, the ascopore is C-shaped, its rim serrated, the frontal pseudopores differ in number, shape and location, and the ovicell lacks the folded proximal margin. A granular ornamentation of the frontal shield is also typical of the Mediterranean species *F. granulosa* sp. nov., which, however, also has a granular endooecium, more numerous trifoliate to quadrifoliate pseudopores arranged in three or four rows between the orifice and ascopore, extensive cryptocystidean lappets lateral to the orifice, a sporadic single distal oral spine, and a denticulate ascopore. The orifice is often distally obscured, with spines (especially the distal one) hidden by the swollen proximal portion of distal autozooids, which appear somewhat imbricated. Lateral walls sloping toward the organic substrate are largely exposed in this species, as seen also in *F. epiphytica* Hayward & Ryland (1995: fig. 13B, C). In our material, the substrate is sometimes partly exposed when autozooids remain unjointed, mostly at triple junctions (Fig. 4D). The spacing of autozooids, the connections through thin joints suggested by the commonly fissure-like windows in the lateral walls (Fig. 4F), the significant reduction or even absence of calcification in the basal walls (Figs 4B, F, 5F, 6D, E), and the apparent weak calcification of all walls (often leading to the collapse of autozooids in many colonies), especially at the margins where autozooids remain incompletely calcified (Fig. 5A), suggest functional adaptations for colonising flexible substrates. Colonies of this species have been found associated with *L. rodriguezii* fronds in high hydrodynamic environments of the Apollo Bank, off NW Sicily (Di Geronimo et al. 1990), and the Ile-Rousse Bank, off NW Corsica. However, these adaptations also lead to the detachment of colonies, and even the disarticulation of individual autozooids, after colony death or in long-term preserved, desiccated colonies (Fig. 6). Loosely connected autozooids have also been observed in *F. commensalis* Viera & Stampar, 2014 as an adaptation to ensure flexibility and growth on its cerianthid host tube (Vieira and Stampar 2014). Similarly, widely exposed lateral walls are also typical features of the possibly cryptogenic species *F. delicia*, mostly associated with the kelp *Agarum cribrosum* Bory, 1826 in the Gulf of Maine, though also reported from hard substrates (e.g., Winston et al. 2000; De Blauwe et al. 2014). Somewhat disjointed autozooids have been observed in *F. granulosa* sp. nov., also from flexible plant substrates, and in *F. dictyota* Hayward & Ryland, 1990 from Tristan da Cunha in the southern Atlantic Ocean.

The ancestrula can be a small autozooid but is usually tatiform and often regenerated as a miniature autozooid. In some cases, including the neotype (Fig. 3), central parts of colonies, where autozooids radiate, show regeneration, with some autozooids exhibiting varying degrees of damage and repair. In these cases, the ancestrula is difficult to identify as two centrally located modules with opposite polarity (with or without signs of regeneration) are present. In two cases documented via SEM, an ovicellate autozooid is adjacent to one of these modules (Fig. 3C). Interestingly, *F. malusii* exhibits three out of four types of ancestrulae typical of the genus, i.e., simply tatiform, tatiform regenerated as a miniaturised autozooid, or a small autozooid-like ancestrula.

Ovicells are notably large relative to autozooids, as indicated by the low ZOvL/OvL ratio (2.15). Variability is evident in the shape (length/width) and size of autozooids, with some abnormalities observed, such as disproportionately large ovicells and deformed maternal and distal autozooids producing the ovicell (e.g., Fig. 5E, F). In one instance, an extremely large autozooid with a dimorphic large orifice was observed at the colony margin, likely resulting from the fusion of two contiguous autozooids, as indicated by the presence of two ascopores and a bifid proximal margin with caudal extensions budded from the preceding autozooid. This may be due to the failure of a zooidal row bifurcation. Similar deformities have also been noted in *F. communis* sp. nov. The occurrence of numerous ovicells (e.g., Fig. 3A, D) in all examined colonies, despite their small size (Fig. 5A), and their early formation, even in periancestrular autozooids or those immediately subsequent (Figs 3B, C, 5A, D), represents a reproductive strategy of this species. These observations in colonies collected during summer align with Bishop’s (1989) theory on the early production of ovicells for larval incubation and release in “spot colonies”, a strategy typical of r-selected species colonising ephemeral substrates, such as seasonally developing algae and/or small, unstable substrates (e.g., Rosso et al. 2014). In this case, the distal (older) parts of *Laminaria* blades, which grow seasonally from the base, are prone to senescence and/or breakage/removal by mechanical forces and feeding activity by organisms.

##### Habitat distribution.

All colonies of *F. malusii* examined have been found on the large, flat, and smooth blades of the fleshy alga *L. rodriguezii*, collected from a rocky elevation swept by strong bottom currents, at a depth of ~60 m, where a particular facies of the Coralligenous biocoenosis develops owing to the local transparency of the water, allowing deep light penetration (Di Geronimo et al. 1990). A few additional colonies originated from *Laminaria* fronds collected off Ile-Rousse, Corsica, at 85–100 m depth. The species was first drawn on a frond of *S. vulgare* by Savigny (1817), indicating a preference for flexible substrates. In contrast, the possible association of *F. malusii* with *Posidonia* meadows currently reported in ecological literature relating to the Mediterranean, remains to be ascertained.

##### Geographical distribution.

Although widely reported from the Mediterranean (and in the Atlantic), after the examination of a great number of colonies and images, we currently confirm the occurrence of *F. malusii* from only two localities: the Apollo Bank near Ustica Island in the SW Tyrrhenian Sea, and a *Laminaria* bank off Ile-Rousse, NW Corsica, in the Liguro-Provençal basin. However, the geographical distribution of the species may be wider than currently recognised, as indicated by its occurrence on *Laminaria*, which in the Mediterranean can extend to relatively deep waters (~100 m), habitats that are less frequently explored. Current presence off the Egyptian coast, site of original description, remains unconfirmed.

#### ﻿ Fenestrulina cavernicola

Taxon classificationAnimaliaCheilostomatidaFenestrulinidae

Rosso & Di Martino sp. nov.

64B20FAD-D626-52A2-9C22-B9977AF23BA7

https://zoobank.org/B991E649-A1D9-40D0-9493-DCE27E87792E

**Figure 7.**
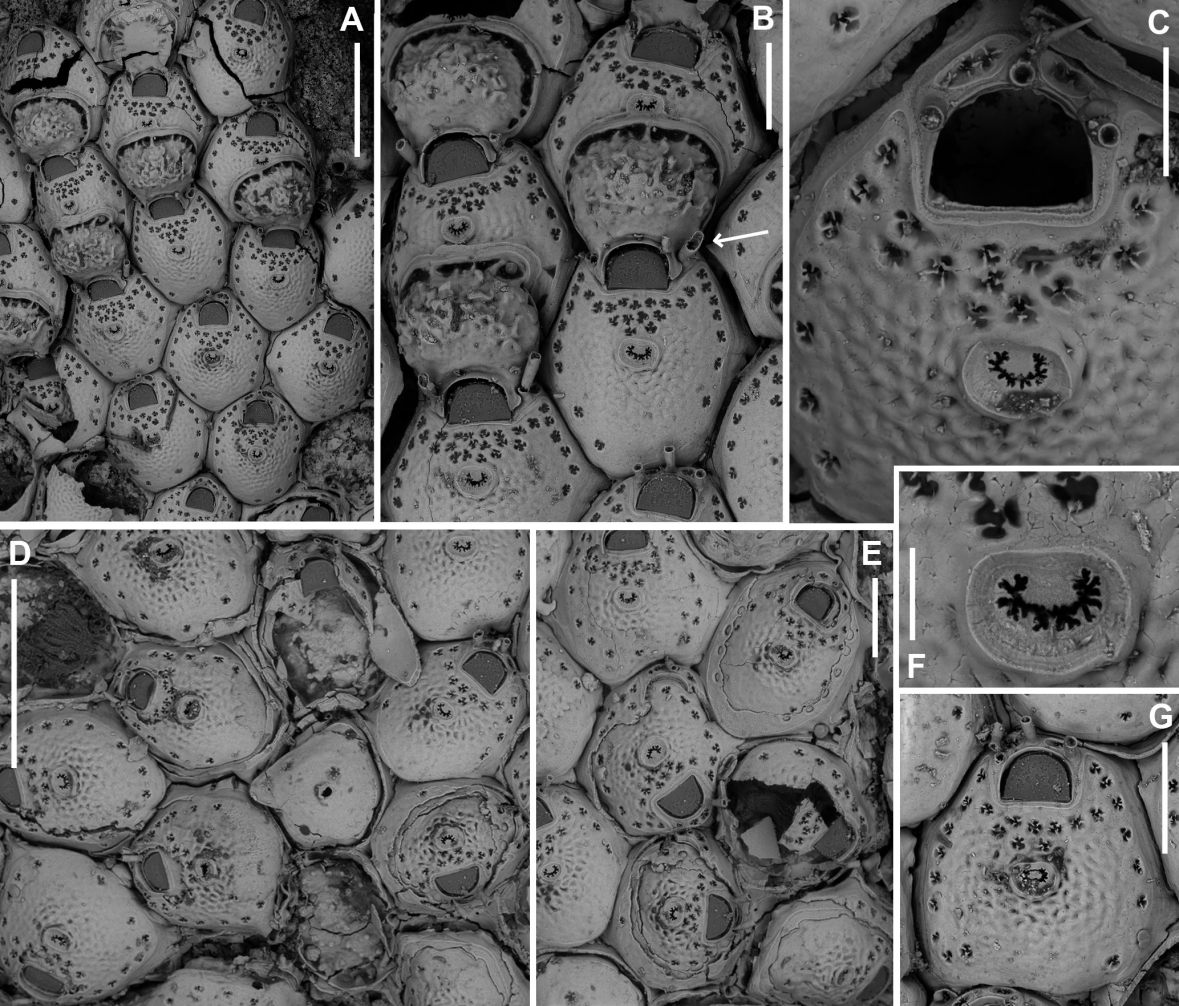
*Fenestrulina cavernicola* Rosso & Di Martino, sp. nov. holotype PMC.B38.23.2.2024.a, Agios Vasilios cave, Lesvos Island, Aegean Sea. **A.** Group of ovicellate and non-ovicellate autozooids. Note the dimpled texture of the frontal shield; **B.** Group of ovicells with rugose endooecium, widely separated from the rudimentary ectooecial rim and proximolaterally indented by two oral spines, which occasionally appear flattened (arrowed); **C.** Distal part of an autozooid showing the orifice with two lateral denticles and three distally placed oral spines. Lateral lappets and distal areas of cryptocystidean calcification are developed, while the gymnocyst just around the orifice is poorly expressed; **D.** Periancestrular autozooids and ancestrula regenerated as a kenozooid with a few pseudopores and a central ascopore with an apparently smooth rim. Multiple regeneration events are evident; **E.** Cluster of damaged and regenerated autozooids, with intramural budding forming in directions even opposite to the original polarity; **F.** Detail of an ascopore and several trifoliate or quadrifoliate pseudopores; **G.** Non-ovicellate autozooid. Scale bars: 500 μm (**A, D**); 200 μm (**B, E, G**); 100 μm (**C**); 50 μm (**F**).

Figs 1, 7, 22, 23, 24; Tables 1, 2

Fenestrulina sp. 1: Rosso et al. 2019b: table 1; Rosso et al. 2019c: table 1, fig. 1e.

##### Type material.

Greece • ***Holotype*** ovicellate colony including ~40 autozooids and the regenerated ancestrula, on a flexible laminar substrate scraped from cave walls. Mediterranean, NE Aegean Sea, Lesvos Island, Agios Vasilios cave; 38.969°N, 26.541°E; 24–40 m depth; summer 2010; scuba diving; V. Gerovasileiou leg.; PMC.B38.23.2.2024.a. Greece • ***Paratypes*** 3 detached colony fragments only consisting of few autozooids; same details as the holotype; PMC.B38.23.2.2024.b1.

##### Diagnosis.

*Fenestrulina* with a dimpled texture of the frontal shield; relatively few tri- to quadrifoliate pseudopores; endooecium prominently rough, bordered by a smooth, low ectooecium, separated by a wide fissure with a few bridge-like connections.

##### Description.

Colony encrusting multiserial, unilaminar; interzooidal communications via pore-chambers, two proximolateral, two distolateral, and one distal.

Autozooids ovoidal to rounded hexagonal, distinct, boundaries marked by narrow, deep grooves (Fig. 7A). Lateral and proximal walls only exposing their upper parts, enlarged at corners, sloping to subvertical. Frontal shield moderately convex, more elevated at ascopore level, with a dimpled texture more evident around the ascopore. Gymnocyst forming a discontinuous narrow rim of calcification distally and laterally to orifice. Cryptocystidean area extensive, outlined by a delicate edge-line, more visible distally, mirroring autozooidal boundary and orifice proximal and lateral margins in non-ovicellate autozooids, diverging laterally in ovicellate ones (Fig. 7B, C), forming subtriangular latero-oral extensions (70–100 μm long). Pseudopores arranged in a single lateral row of 10–20, closely and evenly spaced distally, looser or absent proximally (Figs 7A, B, D, E, G, 23B), near but not leaning on frontal edge. Two, rarely three, additional rows of pseudopores (9–15) between orifice and ascopore; more numerous pseudopores in ovicellate autozooids. Pseudopores on a level with the frontal surface, flower-like in appearance, tri- to quadrifoliate, with three to four laterally compressed spiny processes projecting centrally, unjointed (Figs 7C, F, 23B). Two circular to transversely elliptical cryptocystidean areas distal to orifice, lined by an irregularly lobate rim, including few coalescing pseudopores with numerous spiny processes (Fig. 7C, G), hidden in ovicellate autozooids.

Primary orifice transversely D-shaped, hinge-line straight, lined by a thin and smooth rim of calcification, laterally ending in two denticles near proximal orifice corners; distal rim irregularly undulating (Fig. 7C). Three, rarely two, tubular, slender oral spines, up to ~100 μm long (diameter of the base 15–20 μm), placed distally and/or distolaterally (Fig. 7C, G); spine number remaining constant even in periancestrular zooids (Fig. 7D), two in ovicellate zooids always visible but slightly displaced proximally, occasionally compressed (Fig. 7B, arrowed).

Ascopore nearly central, ~100 μm proximal to orifice (Fig. 7C), lumen transversely C-shaped (Fig. 7C, F), with denticulated rim, situated in a circular to transversally elliptical field of smooth gymnocystal calcification with smooth raised rim, often laterally fusing with arched proximal rim of frontal shield in presence of ovicell (Fig. 7A, B).

Ovicell subglobular, prominent, proximally confined between oral spines, slightly obscuring the orifice distally, seemingly subcleithral, only partially closed by operculum, produced by the distal autozooid (Fig. 7A, B). Endooecium calcified, covered by roughly radial to irregularly undulating, sometimes coalescing crests and isolated spine-like processes, proximally smooth, and protruding in a proximally and upward folded visor-like edge; rimmed by a ~50 μm wide fissure, interrupted by prominent spikes adjoining the surrounding ectooecium, forming a few thin bridge-like structures. Ectooecium a thin and prominent raised rim of gymnocystal calcification, well separated from and capping the endooecium, leaning on proximal side of the frontal raised edge of distal autozooid (Fig. 7B).

Ancestrula tatiform, regenerated as a kenozooid with a central hole (possibly a simple ascopore) and a few pseudopores in the only observed instance (Figs 7D, 24B), budding one distal and two distolateral zooids, and surrounded by six autozooids slightly smaller than subsequent ones.

Additional kenozooids not observed.

##### Etymology.

Referring to the submarine cave habitat that this species typically colonises.

##### Remarks.

*Fenestrulina cavernicola* sp. nov. is most similar to *Fenestrulina juani* Souto, Reverter-Gil & Fernandez Pulpeiro, 2010b, described from detritic bottoms at 57 m depth in the Menorca Channel (Balearic Islands, western Mediterranean). However, *F. juani* differs in the following characters: 1) the frontal shield has a markedly polygonal reticulate pattern covering almost the entire surface; 2) the pseudopores have a pseudo-stellate appearance given by the infundibular shape and spindle-like processes only slightly projecting radially; 3) the pseudopores are more numerous (28–44 with a mean of 35) but smaller (18–27 μm in diameter with a mean of 23); 4) the ascopore is surrounded by a cup-shaped gymnocystal extension, especially prominent proximally, but in *F. cavernicola* sp. nov. it is not as developed; 5) oral spines are usually six, except in periancestrular autozooids having three spines with the proximal pair bifurcating near the base, whereas *F. cavernicola* sp. nov. consistently has three relatively long cylindrical spines in non-ovicellate zooids, with a single instance of lateral compression in the lateral spine of an ovicellate zooid; 6) spines are shifted more proximally, and their base diameter is larger; 7) ovicell ornamentation is more pronounced, with irregular, thick, prominent nodules and regularly spaced bridge-like structures forming in the space between the endo- and ectooecium, before becoming filled by secondary calcification; 8) extensions of the cryptocystidean frontal area lateral to the orifice are similar in ovicellate and non-ovicellate zooids, with the sides starting to diverge at the level of the proximal rim; 9) autozooids are approximately the same size but more elongate (longer and narrower, ZL/ZW: 1.64 vs 1.09) than in *F. cavernicola* sp. nov., and the orifice is proportionately smaller (ZL/OL: 6.77 vs 4.67).

In the dimpled appearance of the frontal surface, *F. cavernicola* sp. nov. is also similar to *F. foveolata* sp. nov., but the latter species differs in several key features: 1) the ovicell has distinct ornamentation, with short, non-indented lateral lappets rimmed by a row of small (15–26 μm in diameter) peripheral pores; 2) frontal pseudopores are fewer, absent proximally, and more often lining the gymnocystal margin; 3) the autozooids, orifices, and ascopore field are smaller; 4) the proximal oral spines are well developed and bifurcated. *Fenestrulina cavernicola* sp. nov. also recalls *F. gelasinoides* Gordon, 1984 from the Kermadec Ridge, but this species has differently shaped frontal pseudopores and a much more prominent dimpled-surfaced ovicell “resembling a golf ball” (Gordon 1984).

Heavily ornamented ovicells, ranging from nodular to wrinkled or irregularly crested, are relatively uncommon in the genus *Fenestrulina.* In addition to *F. juani* and *F. kalliste* sp. nov., they occur in *F. reticulata* Powell, 1967 from New Zealand, *F. horrida* Moyano, 1985 from southern Chile, as well as in *F. cervicornis*, *F. crystallina* Hayward & Ryland, 1990, *F. fritilla* and *F. rugula* Hayward & Ryland, 1990, all from Antarctica. However, the appearance of ovicells in *F. cavernicola* sp. nov. is unique owing to a combination of features, such as the prickly texture of the endooecium, the significant elevation of the thin ectooecium, and the large fissure between them.

Spinulose ascopores occur in some *Fenestrulina* species, but unlike those of *F. cavernicola* sp. nov., they typically show radial processes meeting at the centre, as seen in *F. harmeri* Winston & Heimberg (1986: 28, fig. 69).

In *F. cavernicola* sp. nov., intraspecific variability is limited but regenerative capability, involving both autozooids and ancestrula, is high. The ancestrula has been observed once regenerated as a kenozooid with scant pseudopores and a possible ascopore (Fig. 7D), one of only two such records within the genus. Damaged autozooids are often regenerated one or two times through internal budding, reconstructing from restricted areas around the orifice to almost the entire frontal wall, often involving deflection from the original growth direction or even inversion (Fig. 7D, E).

The occasional occurrence of compressed proximal spines (Fig. 7B) may indicate enlargement before potential bifurcations, although complete or preserved bifurcated spines have never been observed in the available material of this species.

##### Habitat distribution.

To date, *F. cavernicola* sp. nov. has been recorded only from a submarine cave, scraped from both dimly lit and completely dark sectors (Rosso et al. 2019b, c).

##### Geographical distribution.

Currently known only from its type locality, Lesvos Island, northern Aegean Sea, Greece.

#### ﻿ Fenestrulina communis

Taxon classificationAnimaliaCheilostomatidaFenestrulinidae

Rosso & Di Martino sp. nov.

CD90932C-E88D-5A3B-AADD-0F491A3FB905

https://zoobank.org/C5933529-DC05-4DCF-AED4-3CA639BD3466

Figs 1, 8, 9, 10, 11, 12, 22, 23, 24; Tables 1, 2

Fenestrulina malusii (Audouin): Rosso 1989: tables 3d, 4d; Hayward and Ryland 1999: 300, fig. 138A, B; Hayward and McKinney 2002: 81, fig. 37A–D; Rosso et al. 2013: table 1; Rosso et al. 2019a: table 1; Subías-Baratau et al. 2022: table 2, fig. 5a.

##### Type material.

Italy • ***Holotype*** large ovicellate colony encrusting a flat and smooth plastic item, with several lobes, including hundreds of ovicellate and non-ovicellate zooids and the ancestrula. Mediterranean, Tyrrhenian Sea, NW Sicily, Magaggiari beach, Cinisi, near Palermo; 38.159651°N, 13.083972°E; stranded on the beach; Dec. 2023; C. Siddiolo leg.; PMC.B39.23.2.2024.a. Italy • ***Paratypes*** 15 colonies, some small, including only the ancestrula and a few periancestrular autozooids, others large, with ovicellate and non-ovicellate autozooids and often the ancestrula, plus five isolated ancestrulae; same details as the holotype; PMC.B39.23.2.2024.b1–8 on the same plastic item as the holotype; PMC.B39.23.2.2024.b9–15 on a second bent plastic item.

##### Other material examined.

Italy • 2 living colonies on plastic. Mediterranean, Tyrrhenian Sea, NE Sicily, Tono beach, Capo Milazzo Peninsula; 38°14'43"N, 15°14'26"E; stranded on the beach; Apr. 2024; A. Rosso & E. Di Martino leg.; PMC Rosso-Collection I.H.B.116.a. Italy • 1 fragmentary colony, Mediterranean, Ionian Sea, SE Sicily, Plemmirio MPA, Granchi submarine cave, sample GR2; 15°19'40.2"N, 37°01'13.2"E; 20 m depth; Sept. 2009; V. Di Martino leg.; scuba diving • 2 colonies, one on the free valve of the brachiopod *Novocrania anomala* (Müller, 1776) and one on a fragment of the bryozoan *Reteporella elegans* Harmelin, 1976. Mediterranean, Ionian Sea, SE Sicily, Plemmirio MPA, Gymnasium submarine cave, sample GY2; 15°18'48"N, 37°00'12"E; 20 m depth; Sept. 2009; V. Di Martino leg.; scuba diving • 1 fragmentary colony, Mediterranean, Ionian Sea, SE Sicily, Plemmirio MPA, Mazzere submarine cave, sample MZ1; 15°18'35.6"N, 37°00'18.3"E; 20 m depth; Sept. 2009; V. Di Martino leg.; scuba diving; PMC Rosso-Collection I.H.B.116.b. Italy • few zooids on a shell fragment. Mediterranean, Ionian Sea, SE Sicily, Gulf of Noto, sample PS/81 CR1; 36°44'N, 15°10'E; 45 m depth; Jun. 1981; dredge; PMC Rosso-Collection I.H.B.116.e. Italy • 1 colony including the ancestrula and ~30 ovicellate and non ovicellate autozooids on a bioclast. Mediterranean, Ionian Sea, SE Sicily, Ciclopi Island MPA, Ciclopi 2000 cruise, sample 20B; 4145500N, 513130E; 50 m depth; Jul. 2000; A. Rosso leg.; dredging; PMC Rosso-Collection I.H.B.116.c. Italy • few zooids on an alga. Mediterranean, Ionian Sea, SE Sicily, Ciclopi Island MPA, off Santa Tecla, sample ST.1.Z9; 37°38'17"N, 15°10'53"E; 9 m depth; Jun. 2015; M. Catra & R. Leonardi leg.; scuba diving; PMC Rosso-Collection I.H.B.116.d. Spain • 4 small colonies on three benthic plastic debris. Mediterranean, Liguro-Provençal basin, off Palamós; 41.6°N, 3.4°E; 100 m depth; Dec. 2020; B. Figuerola leg.; trawl; PMC EDM-BF Collection SP.H.B.116.f.

##### Diagnosis.

*Fenestrulina* with large frontal pseudopores, partially occluded by a star-shaped plate formed by spinules slender at pore margins, flattening and merging at the centre.

##### Description.

**Figure 8.**
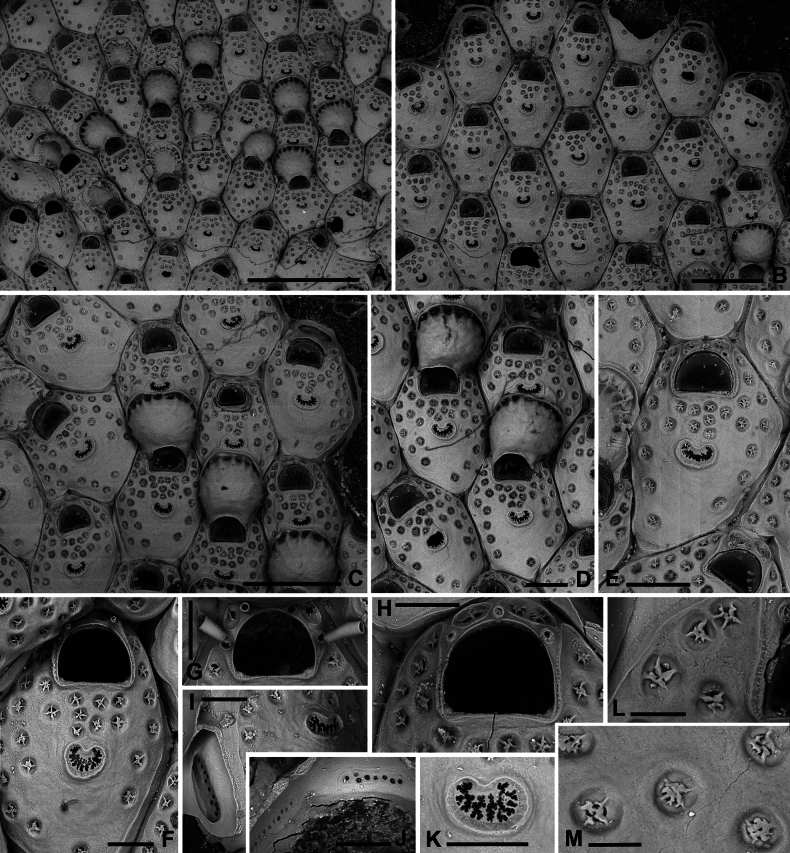
*Fenestrulina communis* Rosso & Di Martino, sp. nov. from a plastic item stranded on Magaggiari beach, Cinisi (Palermo), NW Sicily, Tyrrhenian Sea. **A, C, D.** Paratype PMC.B43.23.2.2024.b8; **B, F, G, K.** Holotype PMC.B39.23.2.2024.a; **E, H, I.** Paratype PMC. B39.23.2.2024.b9; **J, L, M.** Paratype PMC. B39.23.2.2024.b10. **A.** General view of a colony portion showing common ovicellate autozooids; **B.** Non-ovicellate autozooids regularly arranged near the colony margin; **C.** Non-ovicellate autozooids, some distal to ovicells, illustrating morphological variation; **D.** Ovicells with moderately tuberculate surfaces and scalloped distal rims; **E.** Non-ovicellate autozooid; **F.** Autozooid with two oral spines placed very distally and semicircular orifice; **G, H.** Close-up of two orifices with four and three spines, respectively. Note the arched distal rim with few faint denticles and the straight proximal rim with shouldered corners; **I, J.** Multiporous septula seen externally (**I**) and internally (**J**) along lateral and distal walls; **K.** Ascopore with some denticles merging; **L, M.** Frontal pseudopores showing different degrees of development of the stellate process. Note that few pseudopores are present in the proximal frontal shields in panels **A–F**. Scale bars: 1 mm (**A**); 500 μm (**B, C**); 200 μm (**D, E**); 100 μm (**F–K**); 50 μm (**L, M**).

**Figure 9.**
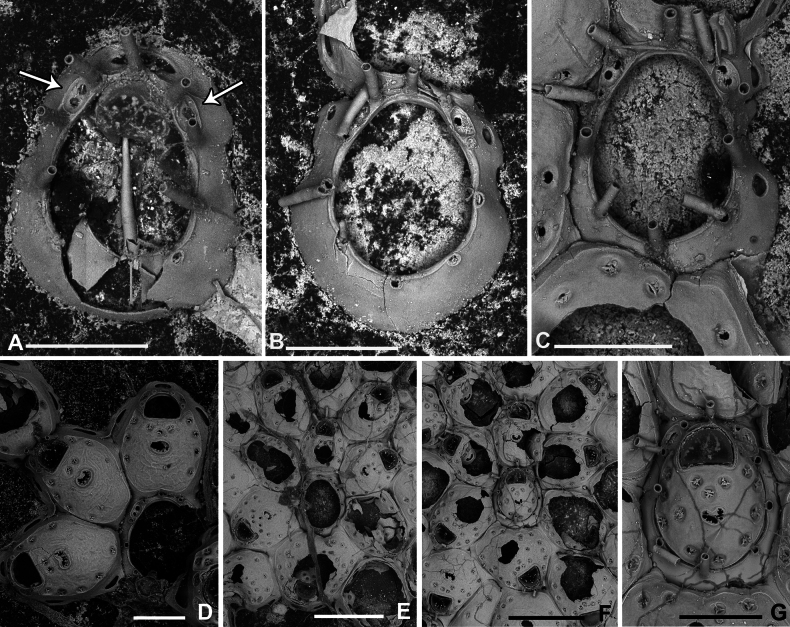
*Fenestrulina communis* Rosso & Di Martino, sp. nov. tatiform ancestrula and periancestrular autozooids from a plastic item stranded on Magaggiari beach, Cinisi (Palermo), NW Sicily, Tyrrhenian Sea. **A.** Paratype PMC. B43.23.2.2024.b.16; **B.** Paratype PMC. B39.23.2.2024.b.17; **C.** Paratype PMC.B39.23.2.2024.b.10; **D.** Paratype PMC.B39.23.2.2024.b.1; **E.** Paratype PMC.B39.23.2.2024.b.8; **F**, **G.** Holotype PMC.B39.23.2.2024.a. **A.** Recently metamorphosed ancestrula with only the distal pore chamber. Note the ten long spines and cryptocystidean areas between oral spines (arrowed); **B.** Ancestrula budding the first distal autozooid, showing two distolateral budding loci, possibly formed after a resorption process; **C.** Close-up of the ancestrula of an asymmetrical young colony with four zooids. The ancestrula shows one distal and one proximal budding locus on one side. Note the large lumina of some incompletely formed pseudopores in periancestrular autozooids; **D.** Another asymmetrical young colony, apparently ceasing growth near an adult conspecific colony; **E.** Tatiform ancestrula typically surrounded by six periancestrular autozooids; **F, G.** Periancestrular area of the holotype (**F**) and a detail of the ancestrula (**G**), regenerated as a miniature autozooid with a central ascopore. Scale bars: 200 μm (**A–D, G**); 500 μm (**E, F**).

**Figure 10.**
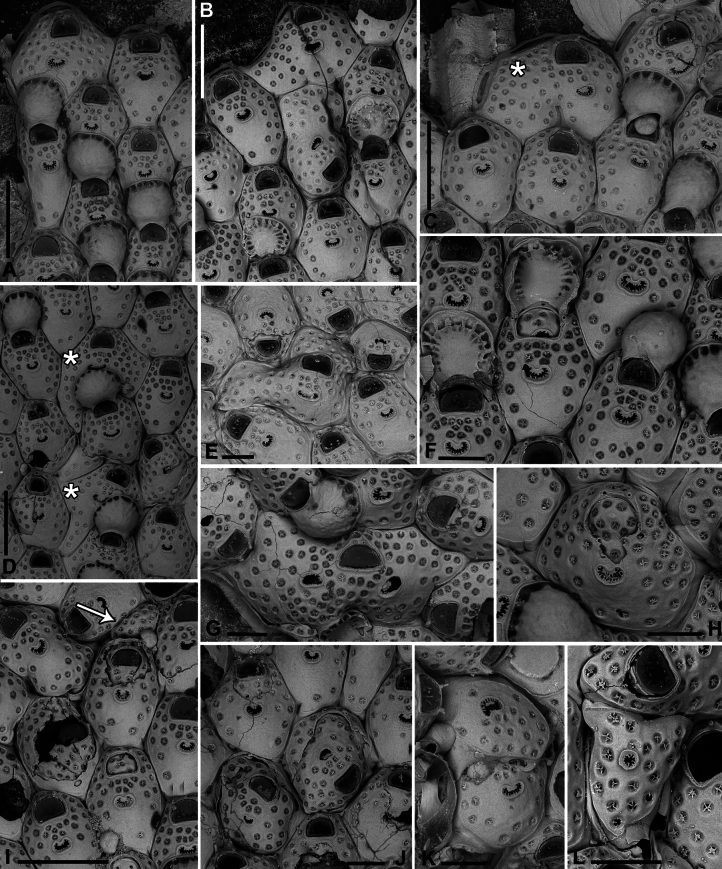
*Fenestrulina communis* Rosso & Di Martino, sp. nov. kenozooids and teratologies from a plastic item stranded on Magaggiari beach, Cinisi (Palermo), NW Sicily, Tyrrhenian Sea. **A–D, F, G, K, L.** Holotype PMC.B39.23.2.2024.a; **E.** Paratype PMC.B39.23.2.2024.b9; **H.** Paratype PMC.B39.23.2.2024.b2; **I.** Paratype PMC.B39.23.2.2024.b7a; **J.** Paratype PMC.B39.23.2.2024.b8. **A.** Wide and slender autozooids; **B.** Enlarged autozooid at colony margin, placed distally to an autozooid with reversed polarity; **C.** Large peripheral autozooids, one possibly resulting from fusion of two neighbouring zooids (asterisk); **D.** Group of autozooids, two showing aberrant morphology (asterisks); **E.** Aberrant autozooids at the contact zone between adjacent colonies; **F.** Ovicell lacking the ectooecium and orifice with a closure plate in a non-functional autozooid; **G.** Irregularly-shaped autozooid with a single orifice and two ascopores, possibly owing to fusion of two autozooids; **H.** Damaged autozooid showing the orifice with a pseudoporous closure plate; **I.** Regenerated autozooids and a small, evenly pseudoporous kenozooid (arrowed); **J.** Regenerated autozooids, including one with reversed polarity; **K.** Two overgrowing kenozooids with C-shaped ascopores and pseudoporous frontal shields; **L.** Kenozooid with an evenly pseudoporous frontal shield and a circular ascopore. Scale bars: 500 μm (**A–D, I**); 200 μm (**E–H, J–L**).

**Figure 11.**
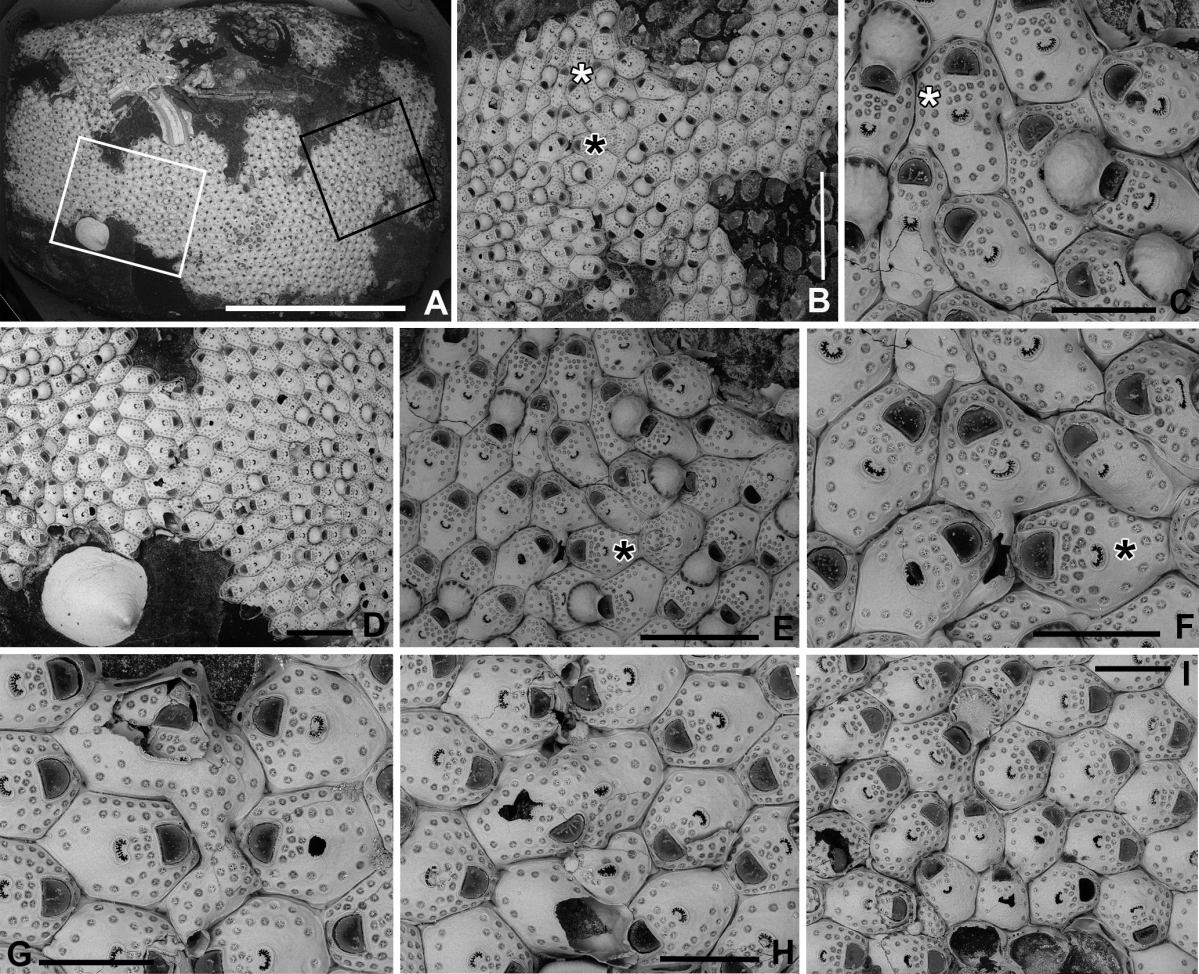
*Fenestrulina communis* Rosso & Di Martino, sp. nov. intraspecific interactions and contact zones between adjacent colonies from a plastic item stranded on Magaggiari beach, Cinisi (Palermo), NW Sicily, Tyrrhenian Sea. Holotype PMC.B39.23.2.2024.a, and paratypes PMC.B39.23.2.2024.b2 and PMC.B39.23.2.2024.b7a, b. **A.** Fragment of the plastic item colonized by *F. communis* sp. nov. showing the lobate holotype extending from the central part of the white frame to the central part of the black frame, surrounded by some paratypes (1–7); **B.** Enlargement of the black-framed portion in panel **A** showing the contact between the holotype and paratype b2; **C.** Autozooid (white asterisk in panel **B**) with lateral prominences, possibly marking the homosyndrome between the holotype and paratype b2; **D.** Enlargement of the white-framed portion in panel **A** showing the contact between the holotype and paratype 7a, and the stand-off between paratypes 7a and 7b near an anomiid bivalve; **E, F.** Progressively enlarged views of the contact zone in panel **B** (black asterisk), highlighting a caudate autozooid to the left of the zooid with asterisk. Note the cryptocystidean areas encircling the orifices in some zooids; **G.** Enlargements of the upper contact area in panel **D**, showing an enlarged autozooid with a zigzag-shaped, expanded caudal region; **H.** Enlargement of the lower contact zone in panel **D**, showing a close-up of an autozooid with two ascopores, indicating fusion of the two colonies. A kenozooid with an elliptical ascopore is also visible; **I.** Enlargement of the area immediately above the anomiid bivalve in panel **D**, showing deflecting zooids at the contact zone between paratypes 7a and b. Scale bars: 1 cm (**A**); 2 mm (**B**); 500 μm (**C–E, G, I)**; 1 mm (**F, H**).

Colony glassy in appearance, encrusting, multiserial, unilaminar, forming large patches up to 1.5 cm^2^ on flat and smooth plastic substrates, with an irregularly lobate outline (Fig. 11A). Interzooidal communications via multiporous septula: two proximolateral (150 μm wide), two distolateral (148–208 μm wide, *n* = 5) and one distal (31–172 μm wide, *n* = 4), located at mid-length along lateral and distal walls (Figs 8B, 9D, 10A–C), each comprising a dozen round pores (6–9 μm in diameter) (Fig. 8I, J).

Autozooids large, roughly hexagonal, distinct, contiguous, boundaries marked by narrow, deep grooves, occasionally widening into subtriangular spaces at triple junctions. Lateral walls sub-vertical, slightly exposed at junctions (Fig. 8C–E). Frontal shield gently marked by a slightly raised rim of smooth calcification lining orifice proximally and laterally, extending distally into long (mean length 148 μm, *n* = 11) lappets on both sides of the orifice (Fig. 8D–F, H). Lappets sometimes encircling and merging beyond orifice in irregular zooids (Figs 10D, G, L, 11F). Surface gently convex, slightly more raised at ascopore level, smooth; circular pseudopores, ~30 per zooid, reduced to 13–16 in periancestrular zooids, increasing to 40 in later autozooids (Fig. 8A–E), more in teratological forms. Pseudopores mainly in distal half, arranged in two or three rows between orifice and ascopore, one or two lateral rows in distal half, often absent/sparse proximally (Fig. 8B–E). Pseudopores irregularly subcircular, slightly infundibular, lumen partly occluded by an irregularly spiny, star-shaped calcification process, depressed in relation to frontal surface, formed by 3–5 spinules progressively flattening and merging centrally, tending to obliterate the lumen but often leaving a small round central opening (Figs 8L, M, 23C). Two, occasionally one, cryptocystidean areas distally to orifice, between spines, each with 1–3 pseudopores (Figs 8E, G, H, 10G, H). Basal wall largely uncalcified.

Primary orifice transversely D-shaped, hinge-line straight with two shoulders at proximal corners; distal rim finely denticulated (Fig. 8F–H). Oral spines usually two, occasionally three, four in periancestrular zooids, ~100 μm long (base diameter 15–20 μm), distally positioned, never proximal than to mid-orifice length (Fig. 8E–H). Ovicellate autozooids with two spines, barely visible in frontal view, lateral to ovicell proximal rim corners.

Ascopore centrally placed, ~130 μm proximal to orifice, distance often exceeding orifice length (Fig. 8A–F); situated in a reniform field of smooth gymnocystal calcification marked by a slightly raised rim, often fusing proximally with arched proximal rim of frontal shield in presence of ovicell; lumen large, transversely C-shaped between the distal short and wide tongue and the arched proximal border; rim denticulate, denticles simple or bi- to trifurcated (Fig. 8C–F), occasionally almost meeting (Fig. 8K).

Ovicell subglobular, prominent, narrowing proximally to fit orifice width, slightly obscuring distal part of orifice, seemingly subcleithral, produced by distal autozooid (Fig. 8C, D). Endooecium calcified, smooth to gently nodular, faintly ribbed at periphery, rimmed by a row of ~15 large, quadrangular pores separated by narrow calcified bridges, giving scalloped appearance; proximal margin on a level with, or just proximal to, proximalmost pair of oral spines, rim slightly folded upwards. Ectooecium reduced to a slightly raised rim of gymnocystal calcification, lining proximal raised edge of distal autozooid.

Ancestrula tatiform (Figs 9, 24C–D), oval but irregularly outlined, smaller than periancestrular autozooids, gymnocyst narrow (60–100 μm wide), more extensive proximally with ten spines, five surrounding orifice, slightly more closely spaced than proximal ones, slightly indenting the raised rim, delimiting the narrower (~15 μm), almost smooth cryptocyst. Opesia oval, occupying almost four-fifth of total length (~300 μm long by 250 μm wide). Two longitudinally elongated (Fig. 9A–C) cryptocystidean areas (each with 1 or 2 pores) between the three distalmost spines and the proximalmost ones, one on each side. Ancestrula first showing only one large distal pore-chamber window connecting it to the first budded distal autozooid; budding pattern: one distal, two distolateral, two proximolateral and one, or rarely two, proximal autozooids, totalling six or seven periancestrular autozooids (Fig. 9). Budding loci seemingly produced after resorption (compare Fig. 9A with Fig. 9B, C). Ancestrula often regenerating as a miniature autozooid (Fig. 9F, G).

**Figure 12.**
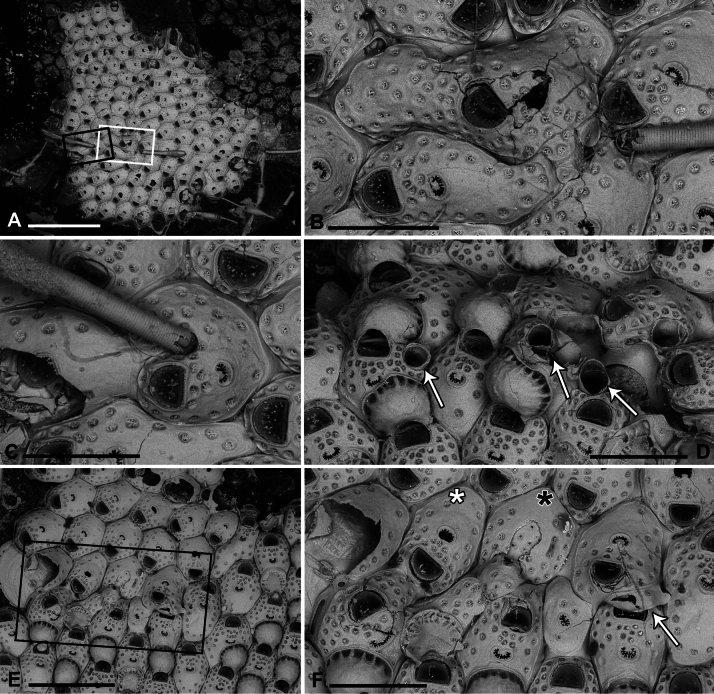
*Fenestrulina communis* Rosso & Di Martino, sp. nov. inter- and intraspecific interactions from a plastic item stranded on Magaggiari beach, Cinisi (Palermo), NW Sicily, Tyrrhenian Sea. Holotype PMC.B39.23.2.2024.a, and paratypes PMC.B39.23.2.2024.b6 and PMC.B439.23.2.2024.b9. **A.** Paratype b9 intergrowing with an *Aetea* colony; **B.** Enlargement of the white-framed area in panel **A** showing deformed autozooids and interzooidal spaces filled with a cluster of small kenozooids lacking ascopores. One erect tube of *Aetea* passes between contiguous *Fenestrulina* autozooids; **C.** Enlargement of the black-framed area in panel **A** showing a deformed *Fenestrulina* autozooid encircling an erect portion of *Aetea*; **D.** Colonies overgrowing an encrusting, unidentified cyclostome whose peristome openings remain unobstructed (arrowed); **E.** Contact zone between the holotype (bottom) and paratype b6, both overgrowing an anomiid bivalve (left); **F.** Enlargement of the black-framed area in panel **E** showing a possible homosyndrome marked by the fusion of two autozooids (white asterisk), a damaged autozooid regenerated as an irregularly elongated kenozooid with an ascopore (black asterisk) and a second small kenozooid (arrow), both connecting the two colonies, the latter elevating on the opposite lobe. A kenozooid with a median ascopore is also visible. Scale bars: 2 mm (**A**); 500 μm (**B–D, F**); 1 mm (**E**).

Kenozooids present, usually observed at colony lobe contacts, between neighbouring colonies, and in damaged areas; from very small (~80 μm) to large, irregularly shaped, in furrows between autozooids, or similar in size to autozooids, irregularly polygonal in shape (Figs 10I, K, L, 11H, I, 12F), with scattered (Fig. 12F) or more densely spaced pseudopores (Fig. 10K, L); the ascopore almost centrally placed, circular to ellipsoidal, evenly denticulated without distal tongue (Fig. 10L), or C-shaped as in autozooids (Fig. 10K), or absent (Fig. 10I).

##### Etymology.

From the Latin *communis*, meaning common, referring to the common/frequent occurrence of this species in multiple samples and localities within the Mediterranean.

##### Remarks.

Colonies reported as *F. malusii* from the British Isles (Hayward and Ryland 1999) and off Rovinj (Croatia) in the northern Adriatic Sea (Hayward and McKinney 2002) resemble *F. communis* sp. nov., especially in the morphology and location of pseudopores, as well as their stellate calcification processes. Autozooids from the British Isles show two or three oral spines, similar to those in our material from stranded plastic debris, which usually bear two very distally located and closely spaced spines (Fig. 8E, F). Three spines are observed less frequently (Fig. 8H), while four spines are exceptional (Fig. 8G). In contrast, specimens from the northern Adriatic Sea are described as having four oral spines, although most figured specimens show three, except for periancestrular autozooids that have five (Hayward and McKinney 2002: fig. 37A and D, respectively). Autozooid measurements reported for these populations, especially those from the northern Adriatic, are slightly smaller than those colonising plastic items in Sicily. This reduced size reflects the prevalence of young colonies, mainly composed of periancestrular autozooids and those in the early astogenetic repetition zone. This interpretation is supported by the transition from four to three oral spines shown in Hayward and McKinney (2002: fig. 37A). Similarly, relatively small autozooids have been documented in colonies from eastern Sicily (Ionian Sea), collected in submarine caves of the Plemmirio MPA (Rosso et al. 2013) or associated with infralittoral algae and circalittoral detritic bottoms at Ciclopi Islands MPA (Rosso et al. 2014, 2019a). These autozooids, however, have two or three oral spines. Unlike the type material, lateral pseudopores in some autozooids, particularly those from submarine caves, tend to develop near or along the marginal elevated rim of the frontal shield.

Additional colonies from unspecified Mediterranean localities may also belong to this species. This includes a colony housed at NHMUK, figured by Wasson and De Blauwe (2014: fig. 4), as well as the specimen figured by Zabala and Madurell in GBIF (2024). In contrast, the colony from Chios Island (Aegean Sea, Greece), identified as *F. malusii* s.s. by Gordon (1984), differs in several respects, including a rugose to crested ovicell, more infundibular frontal pseudopores partially occluded by radial denticles that only occasionally meet at the centre, and a shorter distance between the orifice and the ascopore. This morphotype may represent a distinct species, but additional material is required to confirm its taxonomic identity.

*Fenestrulina communis* sp. nov. is also similar to *F. inesae* Souto, Reverter-Gil & Fernandez-Pulpeiro, 2010a from off Algarve (southern Portugal, Atlantic Ocean), mainly in the stellate appearance of its frontal pseudopores. However, *F. inesae* has ~60 frontal pseudopores, far exceeding the 18–40 observed in *F. communis* sp. nov. Its pseudopores are also significantly smaller in diameter (25 μm vs 31–45 μm). Furthermore, in *F. inesae*, autozooids are slightly shorter (595 vs 646 μm), orifices are longer and comparably more elongate (144 × 160 μm; OL/OW: 0.90 vs 123 × 176 μm; OL/OW: 0.70), ovicells are distinctly shorter and wider than long, unlike the almost isodiametric ones visible in *F. communis* sp. nov. (250 × 323 μm vs 359 × 338 μm), and the ascopore is significantly smaller and longer than wider (89 × 59 vs 74 × 113 μm).

*Fenestrulina communis* sp. nov. shows high variability in autozooid shape and size. Some are highly elongate (Fig. 10A), others notably widened (Fig. 10A, B, D), with widths nearly matching two contiguous autozooids. Some enlarged forms likely result from the fusion of initially separated buds (Fig. 10C). The shape also varies from elongate hexagonal/ovoidal to highly irregular (Fig. 10C, D), with some morphologies seemingly adapted to fill gaps between colonies at contact zones or in damaged colony portions (Figs 10G, 11B, D, E, I). Indeed, colonies densely encrusted the plastic substrate, with at least nine colonies (including two juveniles) counted on ~4 cm^2^, plus additional detached colonies, as indicated by their left traces (Fig. 11A). Interestingly, at colony encounter edges, overgrowth was rarely observed, with irregularly shaped kenozooids forming only in a few cases (e.g., Fig. 10K). More commonly, colonies apparently fused, with autozooids at contact points modified to maximise the encrustation of the available substrate without overgrowing each other. We observed colonies with autozooids: 1) curving and deflecting from their original direction to merge and continue growing alongside (Fig. 11B–F); 2) apparently fusing to form larger ‘double’ autozooids with two widely spaced ascopores, none aligned with the orifice (e.g., Fig. 10G); 3) irregularly shaped along boundaries, some with prominences or cauda-like extensions to connect with autozooids from another colony (Fig. 11B–F). Three similar homosyndrome cases were reported in California for colonies of putative *F. malusii* encrusting *Macrocystis pyrifera* (Linnaeus) C. Agardh and *Agarum fimbriatum* Harvey at 5–25 m depth off Santa Catalina Island, and anthropogenic substrates at the Marine Science Center (Nielsen 1981). There, colony fusion involved ovicell formation, with an autozooid from one colony induced by a maternal zooid from another via its distal pore chamber (Nielsen 1981: fig. 21A, B). The interacting colonies were similar in size and possibly genetically related (Nielsen 1981). Due to the detachment of large colony portions, the full size of interacting colonies in our material remains uncertain. However, their occurrence on the same drift plastic item suggests they originated from one or few pioneer colonies, whose offspring settled nearby.

Interactions between *F. communis* sp. nov. colonies and other bryozoan species were also observed, including an undetermined cyclostome and *Aetea* (Fig. 12A–D). In both instances, *Fenestrulina* colonies overgrew the encrusting skeletal portions of the competitors without obliterating the zooidal openings but only encircling the peristomes (Fig. 12D) and the erect zooidal tubes of *Aetea* (Fig. 12B, C), sometimes deforming their own autozooids (Fig. 12C). Some colonies also interacted with young anomiid bivalves, either overgrowing them (Fig. 12E, F) or ceasing growth at a short distance (Fig. 11A–D), displaying a stand-off behaviour (Taylor and Wilson 2003).

Numerous autozooids show regeneration, often by intramural budding, producing new orifices, partial frontal shields (Fig. 10I, J), entire new autozooids (Fig. 10B), occasionally with reverse polarity (Fig. 10B, J), and kenozooids (Fig. 12F). Closure plates occluding orifices are also common. They resemble the frontal shield, including pseudopores (Fig. 10H, I), and sometimes an ascopore (e.g., Fig. 10F). An ovicell lacking its ectooecium was also observed (Fig. 10F).

##### Habitat distribution.

*Fenestrulina communis* sp. nov. occurs in relatively shaded shelf habitats, ranging from semidark and dark submarine caves at ~20 m depth to deeper (50 m) coarse detritic bottoms swept by currents. Colonies studied by Hayward and McKinney (2002) from near Rovinj, though not explicitly stated, likely originated from alga/plant-rich habitats at less than 40 m depth. The species has also been found at shallower depths (9 m) in the Infralittoral Algae biocoenosis, but is also capable of thriving in well-lit conditions, as evidenced by its settlement and growth on drift plastic items, the source of most studied colonies. Additionally, colonies have been found on benthic plastic items collected at 100 m depth off Catalonia. The colonisation likely occurred while the plastic was still buoyant and floating in shallower waters, before the accumulated encrustation increased its weight, eventually causing it to sink to the seafloor (Subías-Baratau et al. 2022).

##### Geographical distribution.

*Fenestrulina communis* sp. nov. is an Atlanto-Mediterranean species. Its distribution appears to be centred around the British Isles in the Atlantic (Hayward and Ryland 1999), and extends across the Mediterranean, with records from the western Ionian Sea and the Tyrrhenian Sea off the Italian coast, as well as the northern Adriatic Sea off Croatia (Hayward and McKinney 2002). The species’ ability to encrust floating objects, including anthropogenic debris, suggests its opportunistic behaviour and may facilitate its wide distribution across the western Mediterranean, including the Catalan region and the southwestern Tyrrhenian Sea. However, it is plausible that the species also occurs in natural habitats in these areas, as they fall within its known distributional range. *Fenestrulina communis* sp. nov. seems to align with the modern to contemporary concept of *F. malusii*, as demonstrated by the number of synonymies proposed in relation to the limited literature illustrating *Fenestrulina* colonies. Consequently, a thorough revision of existing collections with colonies identified as *F. malusii* from additional sites across the western Mediterranean would likely reveal that they belong to this species rather than *F. malusii*.

#### ﻿ Fenestrulina foveolata

Taxon classificationAnimaliaCheilostomatidaFenestrulinidae

Rosso & Di Martino sp. nov.

C8667C61-D83A-58EB-871A-9A2C28BBB8B2

https://zoobank.org/42868B00-7E3D-45AB-B610-A968F788BFD8

**Figure 13.**
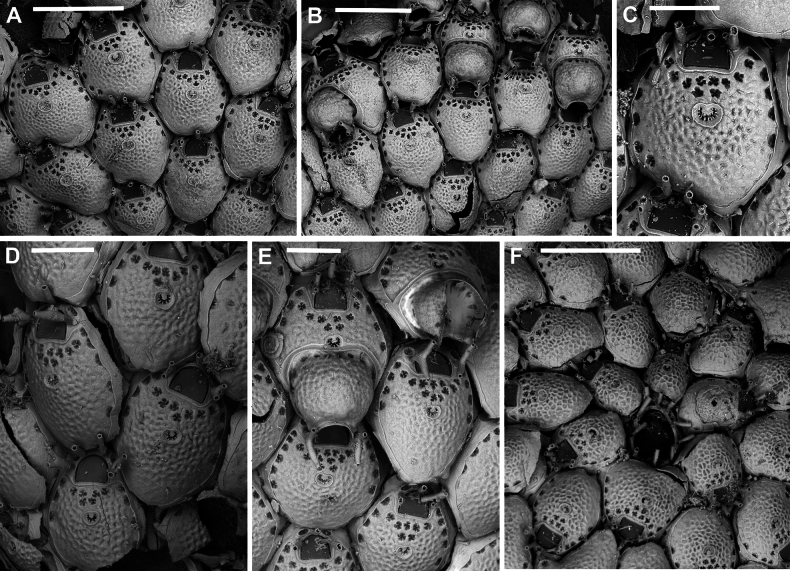
*Fenestrulina foveolata* Rosso & Di Martino, sp. nov. holotype PMC.B40.23.2.2024, Ile-Rousse Bank, NW Corsica, Liguro-Provençal basin **A.** Autozooids with three or four oral spines; the proximal pair are sometimes bifurcated; **B.** Autozooids, some ovicellate. Note the persistence of the bifurcated proximal spine pair proximally to ovicells; **C.** Close-up of an autozooid showing the dimpled frontal shield and the foliate pseudopores often leaning against the cryptocystidean rim; **D.** Autozooids; **E.** Ovicells, including one broken frontally, showing relatively small peripheral pores; **F.** Ancestrula and periancestrular autozooids. Scale bars: 500 μm (**A, B, F**); 200 μm (**C–E**).

Figs 1, 13, 22, 23, 24; Tables 1, 3

##### Type material.

France • ***Holotype*** colony including the ancestrula and some ovicells on fronds of *Laminaria rodriguezii*. Mediterranean, Liguro-Provençal basin, NW Corsica, Ile-Rousse Bank; coordinates not available; 85–100 m depth; 5 Aug. 1957; R/V Président Théodore Tissier survey, St. 423; J.-G. Harmelin leg.; PMC.B40.23.10.2024.a. France • ***Paratypes*** 17 additional colonies and isolated autozooids; same details as the holotype; PMC.B40.23.10.2024.b.

##### Diagnosis.

*Fenestrulina* with partly exposed lateral walls; dimpled frontal shield and ovicell endooecium; endooecium lined by a row of ~15 small peripheral pores and a smooth, low rim of ectooecial calcification; a few tri- to quadrifoliate pseudopores restricted to the distal half of autozooids; transversely C-shaped denticulate ascopore within a subcircular to transversely elliptical gymnocystal field; three or four stout spines, the proximalmost pair bifurcated.

##### Description.

Colony encrusting, multiserial, unilaminar; interzooidal communications via one proximal, two proximolateral, two (occasionally 3 or 4) distolateral, and one distal pore-chamber.

Autozooids ovoidal to round hexagonal, distinct, boundaries marked by narrow, deep grooves (Fig. 13A, B). Lateral and proximal walls steeply sloping to sub-vertical, exposing only their upper parts, generally more expanded and more gently sloping at corners. Frontal shield slightly convex, more elevated at ascopore level, with a dimpled texture, particularly near the ascopore. Gymnocyst forming a discontinuous narrow rim distal and lateral to orifice. Cryptocystidean area extensive, outlined by a raised edge-line, mirroring autozooidal boundary and proximal and lateral margins of orifice, lining it or slightly diverging distalwards in non-ovicellate autozooids, diverging much more in ovicellate ones (Fig. 13C–E), forming subtriangular latero-oral extensions (56–106 μm long), longer in non-ovicellate autozooids. Pseudopores of the frontal shield arranged in a single lateral row of 8–12, irregularly spaced in the distal half of autozooid, absent proximally (Fig. 13A–F), often adjacent to the frontal edge. Two, rarely three, additional rows of pseudopores (6–17) occurring between orifice and ascopore. Pseudopores on a level with frontal surface, spiculate, typically tri- to quadrifoliate, with two to five compressed spiny processes projecting centrally but unjointed (Figs 13C, 23D). Two circular-elliptical cryptocystidean areas, lined by an irregularly lobate rim, occur distal to the orifice, each area seemingly including a single pseudopore or fused pseudopores with numerous spiny processes (Fig. 13D).

Primary orifice transversely D-shaped, hinge-line straight, lined by a thin, smooth rim; proximal and distal rims hidden by opercula. Three, occasionally four, tubular and relatively stout oral spines, up to 80 μm long and 15–20 μm in diameter, placed distally and/or distolaterally (Fig. 13B–E); periancestrular autozooids usually with four spines (Fig. 13F), the proximalmost pair more developed and bifurcated, branches facing upwards; proximalmost bifurcated spines persisting in ovicellate zooids, with distal branches almost leaning against the ovicell (Fig. 13B, E).

Ascopore placed slightly distal to autozooid centre, at variable distance (80–118 μm) from the orifice (Fig. 13C–D), lumen transversely C-shaped, with finely denticulated rim, situated in a sub-circular to transversally elliptical field of smooth gymnocystal calcification marked by a smooth raised rim, often fusing with the arched proximal rim of the frontal shield in the presence of an ovicell (Fig. 13E).

Ovicell subglobular, prominent, slightly obscuring the distal part of the orifice, with short lateral lappets not indented by oral spines, proximolateral corners remaining distal to the spines on each side, seemingly subcleithral, only partly closed by the operculum, produced by the distal autozooid (Fig. 13B, E). Endooecium well calcified, with a dimpled surface similar to autozooid frontal shield, proximally smoother, its narrow rim folding upward; rimmed by a ~30 μm large depression, largely filled by endooecial calcification interrupted by 15 or more marginal pores. Ectooecium consisting of a thin, prominent, raised gymnocystal rim, leaning against the proximal frontal raised edge of the distal autozooid (Fig. 13E).

Ancestrula tatiform (Figs 13F, 24E), with a narrow cryptocystidean rim encircled by ten spines: four distal, more closely spaced; six lateral and proximal, more widely spaced. Budding pattern: one distal, two distolateral and, subsequently, two proximolateral zooids along with a larger proximal autozooid, forming a ring of six periancestrular autozooids.

Kenozooids not observed.

##### Etymology.

From the Latin *fovea*, meaning pit, alluding to the dimpled surface of both the frontal shield and the ovicell endooecium.

##### Remarks.

*Fenestrulina foveolata* sp. nov. mostly resembles *F. cavernicola* sp. nov. but differs in having a distinct ornamentation of the ovicell, with short, non-indented lateral lappets rimmed by a row of small peripheral pores, fewer frontal pseudopores, absent proximally, and more often adjacent to the gymnocystal margin, smaller autozooids, orifices, and ascopore field, and proximal oral spines well developed and bifurcated. A dimpled, but less pronounced, ooecial surface also occurs in *F. ovata* sp. nov., which, however, lacks bifurcated spines and has spine bases only barely visible in ovicellate autozooids. This species also has an ovicell with arcuate lateral lappets overarching the lateral sides of the orifice, absent in *F. foveolata* sp. nov. The dimpled surface of both autozooids and ovicells is also reminiscent of *F. gelasinoides*, but that species has stronger ornamentation, occluded autozooidal pseudopores and inconspicuous, unbranched oral spines in ovicellate autozooids. Based on Gautier’s (1962) report of a bifurcate proximal pair of oral spines (p. 170) and of deep colonies with a more granulose frontal shield (p. 171), at least some of his *F. malusii* material may belong to this species or to *F. kalliste* sp. nov.

##### Habitat distribution.

*Fenestrulina foveolata* sp. nov. has been found only on *Laminaria* fronds, co-occurring with *F. malusii*.

##### Geographical distribution.

*Fenestrulina foveolata* sp. nov. is currently known only from its type locality off Corsica (Ile-Rousse Bank), in the Liguro-Provençal basin.

#### ﻿ Fenestrulina granulosa

Taxon classificationAnimaliaCheilostomatidaFenestrulinidae

Rosso & Di Martino sp. nov.

6C0CE304-BE73-53EB-9ED3-F42640AA2361

https://zoobank.org/F12D60BA-1B0C-4E0A-B040-5F633ACCEAA5

**Figure 14.**
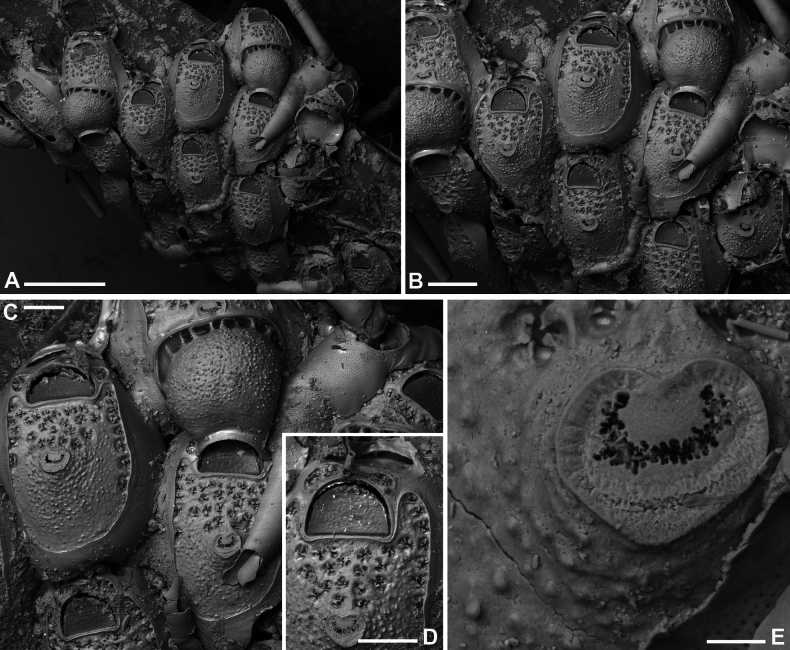
*Fenestrulina granulosa* Rosso & Di Martino, sp. nov. holotype NHMUK 2009.11.2.2, Dhiaporia, Chios, Aegean Sea. **A.** General view of the dry-preserved colony on a *Posidonia oceanica* leaf; **B.** Cluster of some better preserved autozooids, two ovicellate; **C.** Close-up of two autozooids and an ovicell, showing the characteristic finely granular calcification; **D.** Close-up of the distal part of an autozooid. Note the denticulate/undulating distal margin of the orifice; **E.** Close-up of the ascopore and a pseudopore with centrally unjointed radial spine-like processes. Scale bars: 500 μm (**A**); 200 μm (**B**); 100 μm (**C, D**); 20 μm (**E**).

Figs 1, 14, 22, 23; Tables 1, 3

Fenestrulina malusii : Hayward 1974: table 1, pars; Hayward 1975: table 1, pars.

##### Type material.

Greece • ***Holotype*** ovicellate colony on a *Posidonia* leaf including a few dozen autozooids, without ancestrula. Mediterranean, Aegean Sea, Chios, Dhiaporia; University College Swansea Expedition to Chios; 38°20'N, 26°00'E; 30 m depth; Aug. 1967; scuba diving. NHMUK 2009.11.2.2.

##### Diagnosis.

*Fenestrulina* with well-exposed lateral and proximal walls, finely granular frontal shield with a centrally located C-shaped ascopore; pseudopores mostly restricted distally in three or four rows between the orifice and ascopore, and in a single proximally incomplete peripheral row; orifice with an irregularly denticulated distal margin and a single distal spine concealed in ovicellate autozooids; ovicell endooecium finely granular except for the proximal folded rim.

##### Description.

Colony encrusting, multiserial, unilaminar; interzooidal communications via two proximolateral, two distolateral and one distal pore-chambers, externally visible as elongate, elliptical windows.

Autozooids ovoidal, distinct, with wide grooves in-between (Fig. 14A–C); vertical walls gently sloping, largely exposed proximally and laterally, sometimes revealing the substrate at triple junctions (Fig. 14C). Frontal shield moderately convex, more elevated centrally at ascopore level. Gymnocyst present only distally and laterally to the orifice. Cryptocystidean area finely granular, granules ~5 μm in diameter, more raised centrally, but attenuating and smaller to absent towards the margins; marked by a thin raised rim, distally lining the orifice proximally and laterally, extending up to half its length or more (Fig. 14C), forming blunt subtriangular latero-oral extensions of variable length (39–88 μm long). Pseudopores of the frontal shield numbering 26–34, closely spaced in a single row along autozooid distal half, occasionally extending more proximally, with four, rarely three, additional irregular rows of pseudopores (18–20) between orifice and ascopore (Fig. 14B–D). Pseudopores 26 μm in maximum dimension, on a level with frontal surface, tri- to quadrifoliate, with 3–5, occasionally more, spiny, platy or denticulate processes converging centrally but remaining unjointed (Figs 14E, 23E). Two relatively small (38–58 μm wide), subcircular to subelliptical or larger, arched and elongate (~75 μm long), smoothly-rimmed cryptocystidean areas distal to the orifice, occasionally shifted laterally (Fig. 14C, D), each bearing 1–3 pseudopores with numerous spiny processes, exposed also in ovicellate zooids (Fig. 14C).

Primary orifice transversely D-shaped, hinge-line straight; distal rim with an irregularly denticulate shelf (Fig. 14D). A single tiny spine (base diameter ~13 μm) located mid-distally to the orifice (Fig. 14D). Spines absent in ovicellate zooids (Fig. 14B, C), the mid-distal one remaining concealed beneath the ovicell.

Ascopore centrally placed, 119–171 μm proximal to orifice (Fig. 14B–D), the lumen transversely C-shaped, with a strongly irregular denticulate rim, some denticles leaf-shaped with 3–5 smaller denticles; situated in a cordiform-to-reniform field of flat gymnocystal calcification with a peripheral radially ribbed band, smooth-rimmed, slightly raised proximally on the frontal shield surface; fusing with the arched proximal rim of the frontal shield when distal to an ovicell (Fig. 14B, C).

Ovicell globular and slightly elongate, prominent, narrowing proximally, obscuring the distal part of orifice, seemingly subcleithral, produced by the distal autozooid (Fig. 14A–C). Endooecium well calcified, finely granular, granules more prominent and more densely spaced distally, attenuating and reducing proximally to a thin (~20 μm), smooth tubular proximal edge; peripheral row of 14–16 subquadrangular (each 20–48 μm wide) or occasionally elongate (up to 80 μm) pores.

Ancestrula and kenozooids not observed.

##### Etymology.

From the Latin *granulosus*, meaning granular, in reference to the distinctive granular surface of both the frontal shield and the ovicell endooecium, a unique feature among all known species of the genus.

##### Remarks.

*Fenestrulina granulosa* sp. nov. resembles *F. malusii* in the granulation of the frontal shield and the ovicell endooecium, the shape of the pseudopores and their location restricted to the distal portion of the autozooidal frontal shield, and the raised rim demarcating the frontal cryptocystidean area from the widely exposed lateral walls. However, the two species can be readily distinguished. In *F. malusii*, the granules are significantly fewer and less pronounced. Additionally, *F. malusii* has a distinctive ascopore with a circular gymnocystal field and a smooth-rimmed lumen, the autozooids are slightly smaller and comparatively squatter, while the orifice is noticeably smaller and protected by three or four distal spines, with the proximal pair persisting in ovicellate autozooids. Granular ornamentation is rare within the genus, occurring only in *F. malusii* and *F. granulosa* sp. nov. in the Mediterranean, and in a few additional species globally. A similar granulation on both the autozooidal frontal shield and the ovicell is observed in *F. antarctica* Hayward & Thorpe, 1989, a species recorded from the Palmer Archipelago, Bellingshausen Sea and Ross Sea. This species, however, differs in having stellate pseudopores in the frontal shield of very large autozooids that do not expose lateral walls and lack a cryptocystidean rim; oral spines are absent and the ovicell proximal rim joins the proximal corners of the orifice. A somewhat similar but sparser granulation on the frontal shield is present in an unnamed species from Safaga Bay (see Remarks for *F. malusii*). Large, prominent, almost tubercular granules also occur on the ovicell and the proximal lobe of the tubular collar of *F. personata* (MacGillivray, 1883) from southern Australia and New Zealand. However, this species has a distinctively smooth frontal shield, a smooth-rimmed ascopore, sparse non-radiate pseudopores that are simple holes, the absence of such pores between the orifice and ascopore, and a distributional pattern opposite to that seen in other *Fenestrulina* species.

The widely exposed and gently sloping lateral walls of *F. granulosa* sp. nov., which reduce the contact surface between adjacent autozooids, may represent specialised adaptation to minimise colony breakage when on flexible substrates (see *F. malusii*).

The studied colony was among those examined by Hayward (1974) and identified as *F. malusii* from six different localities around the Isle of Chios: Emborios Bay, Cape Mastika, Venetica, Kokkina, Foradhas, and Dhiaporia. Unfortunately, most of this material could not be located and therefore remains unexamined. Consequently, it remains uncertain whether all the specimens belong to *F. granulosa* sp. nov. or if they represent additional species.

##### Habitat distribution.

The only examined colony of *F. granulosa* sp. nov. encrusts a flexible organic substrate, specifically a *Posidonia* leaf (A. Herdman, pers. comm., Oct. 2024), which is heavily colonised by several bryozoan species. This aligns with the shallow-water range of the collections of the University College Swansea Expedition to Chios, as reported by Hayward (1974), which did not exceed 61 m and was predominantly within the first 50 m, encompassing the depth range of *Posidonia* meadows. However, owing to the grouping of stations from different localities and the limited, sporadic information on species records other than *F. malusii*, drawing further conclusions about the habitat is challenging. If we assume that all of Hayward’s (1974) records pertain to the same species (difficult without specimens), *F. granulosa* sp. nov. may also inhabit various environments, including submarine caves, rocky infralittoral habitats, and bioconstructions (possibly coralligenous habitats), in addition to *Posidonia* meadows and associated *Pinna* valves.

##### Geographical distribution.

*Fenestrulina granulosa* sp. nov. is currently known only from its type locality off Chios Island, in the north-eastern Aegean Sea. While no precise collection site is indicated, the examined colony originates from one of the sampling stations of the 1967 University College Swansea Expedition to Chios, whose material was later studied by Hayward (1974).

#### ﻿ Fenestrulina kalliste

Taxon classificationAnimaliaCheilostomatidaFenestrulinidae

Rosso & Di Martino sp. nov.

8E910AC6-FFCA-5DE7-96CE-AA3EF4C2D876

https://zoobank.org/98EE9DC5-D525-4E3B-918A-9A9071A1F194

**Figure 15.**
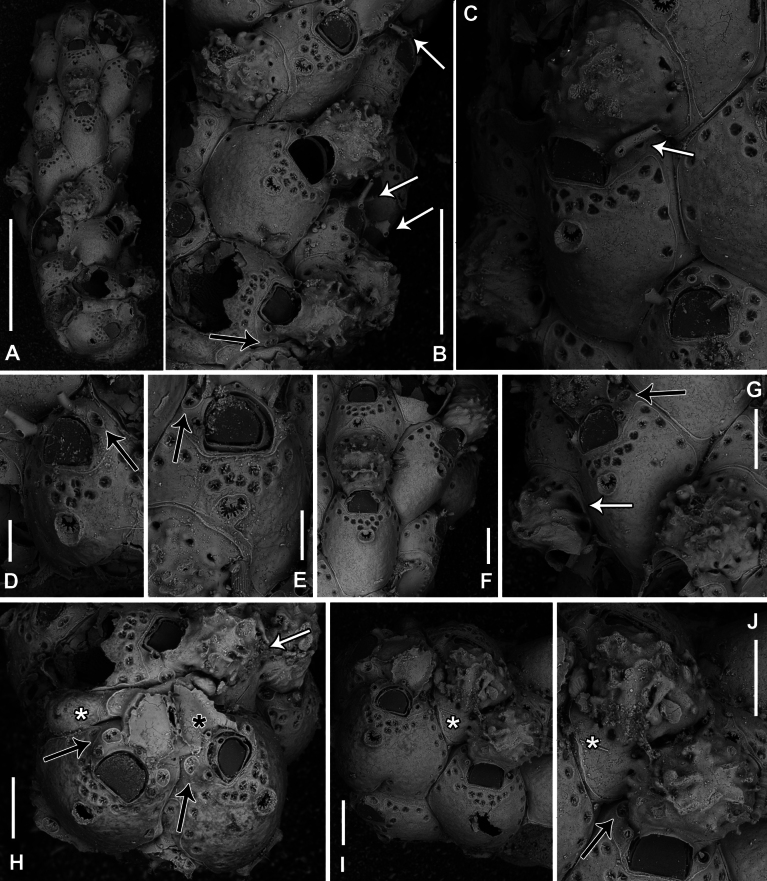
*Fenestrulina kalliste* Rosso & Di Martino, sp. nov. holotype PMC.B41.23.2.2024.a, Calvi, NW Corsica, Liguro-Provençal basin. **A.** General view of the colony; **B.** Ovicellate autozooids. Note the tuberculate-to-wrinkled appearance of the ovicells, the bifurcate spines (white arrows), and the exposed cryptocystidean areas lateral to the ovicells (black arrow); **C.** Ovicellate autozooid with one bifurcated oral spine still in place (arrowed). Note the prominent rim of the ascopore field and the ovicell pores distally occluded by secondary calcification; **D.** Autozooid with three spines and exposed cryptocystidean areas (arrowed); **E.** Regenerated autozooid with orificial cryptocystidean area (arrowed); **F.** Colony portion showing variability in ovicell ornamentation; **G.** Differently inclined ovicells showing the prominence and morphology of their proximal rim. A particularly large ovicell pore (white arrow) and cryptocystidean areas distal to the orifice (black arrow) are indicated; **H.** Broken twin ovicells in two contiguous autozooids formed by the same kenozooid (white asterisk); distal spine still evident in one case (black asterisk); exposed cryptocystidean areas are black arrowed; lateral view of the kenozooidal ovicell depicted in **I** and **J** (white arrow); **I**, **J.** Overview and detail of an ovicell formed by a distal kenozooid (asterisks). Arrow: exposed cryptocystidean area. Scale bars: 1 mm (**A**); 500 μm (**B**); 200 μm (**C, F, G–J**); 100 μm (**D, E**).

Figs 1, 15, 22, 23; Tables 1, 3

Fenestrulina malusii (Audouin): Rosso 1989: tables 5a, 6d.

##### Type material.

France • ***Holotype*** 1 dead ovicellate colony on a phidoloporid fragment including ~30 autozooids, without ancestrula on biogenic debris. Mediterranean, Liguro-Provençal basin, NW Corsica, Calvi; R/V *Catherine Laurence*; Bracors-3, Stn CL 74-12B; 42°34'35"N, 8°41'23"E; 110 m depth; Jun. 1983; PMC.B41.23.2.2024.a.

##### Diagnosis.

*Fenestrulina* with smooth frontal shield; scant number of pseudopores both peripherally and between the orifice and the ascopore; branching proximal spines persisting on ovicellate autozooids; prominent nodular ovicell ornamentation.

##### Description.

Colony encrusting, multiserial, unilaminar; interzooidal communications via two proximolateral, two distolateral and one distal pore-chambers, externally visible as elongate, elliptical windows, internally as multiporous septula.

Autozooids rounded hexagonal, distinct, separated by narrow, deep grooves (Fig. 15A, B, F). Upper vertical walls of autozooidal distal half slightly exposed, more evident at triple junctions (Fig. 15C, G), deeply sloping. Frontal shield smooth to gently nodular, moderately convex, more elevated at ascopore level. Gymnocyst present only distal and lateral to orifice. Cryptocystidean area marked by a thin raised rim, lining proximal margin of orifice, diverging laterally (Fig. 15C, E, G, J), forming blunt subtriangular latero-oral extensions (~100 μm long). Pseudopores of the frontal shield irregularly shaped, slightly infundibular, arranged in a single lateral row, usually restricted to the distal half of the autozooid, occasionally present proximally (Fig. 15C, I). One to two additional irregular rows of pseudopores (9–12) between orifice and ascopore. Pseudopores on a level with frontal surface, circular to irregular, with 1–4, mostly three, laterally compressed spiny processes converging centrally but not fusing at their tips (Figs 15C–E, 23F). Two relatively large (50–67 μm wide), subelliptical, smoothly-rimmed cryptocystidean areas distolateral to orifice, between or distal to spines (Fig. 15C–E), each bearing one or two pseudopores with numerous spiny processes, exposed also in ovicellate zooids (Fig. 15B, G–J).

Primary orifice transversely D-shaped, hinge-line straight, lined by a smooth thin rim of calcification, ending in two denticles near proximal corners of orifice; distal rim smooth (Fig. 15B). Three tubular oral spines along the arched distal rim of orifice (Fig. 15D, E), mid spine thinner (base diameter 19–26 μm) than proximal ones (27–35 μm at the base, widening). Proximal spines bifurcating (Fig. 15B, C arrowed, D) at ~50 μm from the base, maximum diameter 42 μm; proximal branch smoothly rimmed at bifurcation level, presumably the site of an articulation missing in all available material; distal branch at least up to ~100 μm long. Ovicellate zooids with two spines at ovicell proximal corners (Fig. 15B, C, F–J), distal spine concealed but persisting underneath (Fig. 15H, black asterisk).

Ascopore relatively distal, ~94 μm proximal to the orifice (Fig. 15D, E), lumen transversely C-shaped, rim strongly denticulated, denticles simple to leaf-shaped with 3–5 smaller denticles; set in circular to transversely elliptical field of smooth gymnocyst, smooth-rimmed, flared, vertically protruding from the shield surface; often fusing with the arched proximal rim of the frontal shield when distal to an ovicell (Fig. 15B, C, E).

Ovicell globular, slightly elongate, prominent, narrowing proximally, obscuring the distal part of orifice, seemingly subcleithral, produced by the distal autozooid (Fig. 15B, C, F, G) or by a small polygonal to irregularly elongate kenozooid (Fig. 15H–J, white asterisks). Endooecium well calcified, tuberculate-to-rugose, radial patterned, crossed by transverse crests, proximally smooth, proximal edge thin and slightly (~20 μm) folded upwards; with a sub-peripheral row of a dozen circular pores (~20 μm in diameter), barely detectable frontally (Fig. 15A, C, F, G), occasionally coalescing into a single elongate, 68 μm long, pore (Fig. 15G, white arrow). Ectooecium with a thin, gently raised rim of gymnocyst lining proximal edge of distal autozooidal cryptocystidean area.

Kenozooids with a triangular (Fig. 15I, J) to irregularly elongate (Fig. 15H) visible portion, lacking pseudopores and ascopore, apparently exclusively produced in connection to ovicell formation.

Ancestrula not observed.

##### Etymology.

From the Greek *kalliste* (*καλλίστη*), meaning “the most beautiful”, used as a noun in apposition, referring to the name given by ancient Greeks and later by J.J. Rousseau to Corsica, from where the material of this species originates. Kalliste is also the name of a marine nymph, the daughter of the sea-god Triton and Libya of Egypt.

##### Remarks.

*Fenestrulina kalliste* sp. nov. resembles *F. cavernicola* sp. nov., and especially *F. juani*, in having a markedly ornamented, elongate ovicell (OvL/OvW: 1.13). However, *F. kalliste* sp. nov. has smaller nodules, often aligned to form roughly radial to transverse crests, while *F. cavernicola* sp. nov. has spiny processes on the endooecium, and *F. juani* very prominent, thick and rounded nodules. Similarities with *F. juani* include bifurcated proximal oral spines and the protruding ascopore gymnocystal field that in *F. juani* is significantly more prominent, especially proximally, becoming asymmetrically cup shaped. The ascopore, in *F. juani*, is larger (80 × 105 μm vs 71 × 90 μm), but the lumen is smaller (20 × 39 μm vs 43 × 54 μm), giving it a different appearance. The frontal shield pattern also differs: smooth in *F. kalliste* sp. nov., dimpled to reticulate in both *F. cavernicola* sp. nov. and *F. juani*. Furthermore, *F. juani* has fewer, larger pseudopores at the autozooidal periphery and between the orifice and the ascopore, which are depressed, infundibular and pseudostellate. In contrast, *F. kalliste* sp. nov. has pseudopores on a level with the frontal shield, tri- to quadrifoliate, spinulose. Oral spines in periancestrular autozooids are more numerous (up to six) in *F. juani*. Zooids are more elongate in *F. juani* than in *F. kalliste* sp. nov. (ZL/ZW: 1.64 vs 1.23), with a proportionally smaller orifice (ZL/OL: 6.77 vs 3.76). The ovicell is also smaller in *F. juani* (285 × 303 μm vs 369 × 326 μm), especially in comparison with autozooidal dimensions, and proportionately wider than long. Similar but subtler size differences exist between *F. kalliste* sp. nov. and *F. cavernicola* sp. nov., with the latter species having larger autozooids but relatively smaller orifices. Bifurcated proximal spines, as in *F. kalliste* sp. nov., also occur in *F. foveolata* sp. nov., which however differs in ovicell and frontal shield texture, among other features. Some periancestrular autozooids of *F. malusii* also show bifurcate proximal spines and *F. cavernicola* sp. nov. may possess them, as suggested by spines with occasionally flattened terminations (Fig. 7B). This character, reported for *F. juani*, appears relatively common among Mediterranean species, yet globally it is known from only a few other southern hemisphere species: *F. cervicornis* and *F. dictyota* Hayward & Ryland, 1990 from Antarctica (Hayward and Ryland 1990), and *F. disjuncta* (Hincks, 1885) (see Gordon 1984) and *F. littoralis*Gordon (2009: fig. 13) from New Zealand. The holotype of *F. kalliste* sp. nov. shows two ovicell-forming kenozooids with flat, smooth surfaces lacking pseudopores and ascopores (Fig. 15H–J), a previously unreported character in the genus. Some colonies described by Gautier (1962) as *F. malusii* may belong to this species based on his mention of a bifurcate proximal pair of oral spines, or to *F. foveolata* sp. nov.

##### Habitat distribution.

To date, *F. kalliste* sp. nov. has been found only in organogenic sediments collected from an outer shelf setting at 110 m depth, where the Offshore Detritic Bottoms biocoenosis occurs (Rosso 1989; Emig 2018).

##### Geographical distribution.

*Fenestrulina kalliste* sp. nov. is currently known only from the type locality, off Calvi. At least part of the material examined by Gautier (1962) might belong to this species. Most of his colonies also originate from the same geographical area of our type (Mediterranean coast of France). However, some come from other Mediterranean localities, suggesting a potential wider geographical distribution. Gautier’s collection needs revision.

#### ﻿ Fenestrulina ovata

Taxon classificationAnimaliaCheilostomatidaFenestrulinidae

Rosso & Di Martino sp. nov.

F1AAED4E-3B0A-551B-A7F4-D257B9737D50

https://zoobank.org/87FEE866-82B2-47AD-91FB-52BDDBBC1E0C

**Figure 16.**
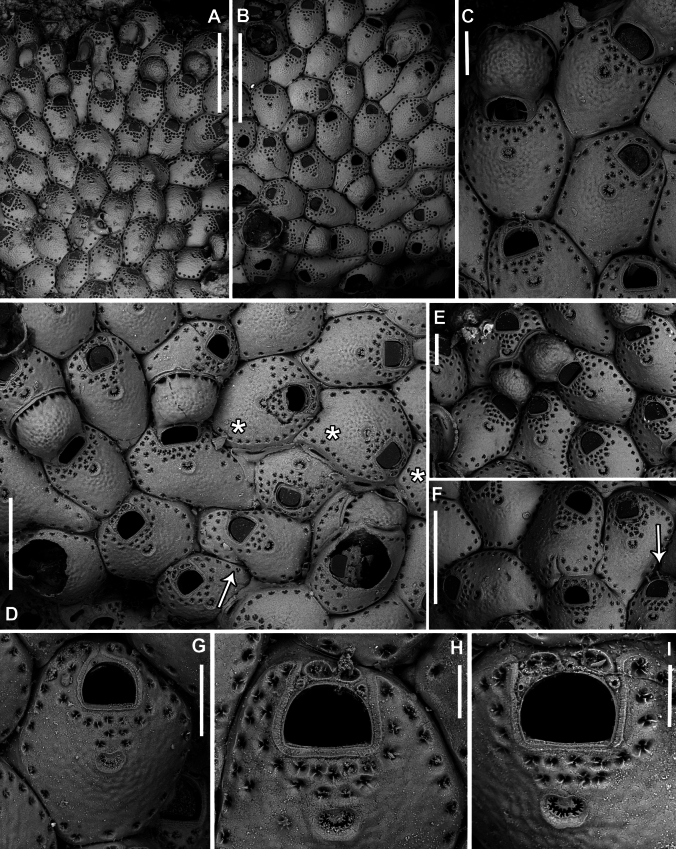
*Fenestrulina ovata* Rosso & Di Martino, sp. nov. **A.** Holotype PMC.B42.23.10.2024.a, calanque of Port Miou, Cassis, Liguro-Provençal basin; **B–D, G–I.** Paratypes PMC.B42.23.2.2024.b, Trémies cave, Cassis, Liguro-Provençal basin; **E, F.**PMC Harmelin Coll. PMC.B42.23.2.2024.b. Trémies cave, Cassis, Liguro-Provençal basin. **A.** Holotype multilayered colony; **B.** Central colony part; **C.** Group of autozooids, two ovicellate, showing the ovate morphology of the ovicell with collar-like extensions nearly completely hiding the oral spines; **D.** Close-up of autozooids in the bottom right portion of panel **B**. Some autozooids (marked with asterisks) overgrow a previous colony layer. The asterisked autozooids, laterally budded by the central ovicellate autozooid, and those in the upper right, elevate to overgrow autozooids from the same layer. Note also the irregular morphology of the central ovicellate autozooid and the proximal one, which show an abnormal cryptocystidean lateral lappet encircling the orifice (white arrow); **E.** Two ovicells produced by a single deformed distal autozooid; **F.** Relatively large autozooid with evidence of bud fusion at its proximal end. Note the occasionally preserved, thin and short oral spines (arrowed); **G–I.** Close-ups of autozooids showing the morphology of pseudopores and ascopore, as well as orificial characters, including the pattern in oral spine number (1–3), development of lateral lappets, and distal cryptocystidean areas. Scale bars: 1 mm (**A, B**); 500 μm (**D, F**); 200 μm (**C, E, G**); 100 μm (**H, I**).

Figs 1, 16, 22, 23; Tables 1, 4

##### Type material.

France • ***Holotype*** 1 ovicellate, lobate, locally multilaminar owing to self-overgrowth colony, encrusting the inner side of a cemented bivalve on an old coralligenous concretion. Mediterranean, Liguro-Provençal basin, Cassis, calanque of Port Miou, Stn-JGH-73.06; 43°12'12.00"N, 5°30'51.74"E; 17 m depth; 18 Mar. 1973; J.-G. Harmelin leg.; PMC.B42.23.10.2024.a. France • ***Paratypes*** 3 ovicellate colonies on the same coralligenous concretion; same details as the holotype; PMC.B42.23.10.2024.b1–3. France • 9 ovicellate multilaminar colony fragment detached from its substrate. Mediterranean, Liguro-Provençal basin, Cassis, Trémies cave, left chamber; 43°12'00"N, 5°30'50"E; 6 m depth; 2 May 1985; scuba diving; J.-G. Harmelin leg.; PMC.B42.23.10.2024.b4–12. France • 6 ovicellate multilaminar fragmentary colonies on small limestone substrates. Mediterranean, Liguro-Provençal basin, Cassis, Trémies cave, dark zone B; 43°12'00"N, 5°30'50"E; 8 m depth; 7 Jan. 1982; scuba diving; J.-G. Harmelin leg.; PMC.B42.23.2.2024.b13–18.

##### Diagnosis.

*Fenestrulina* with multilaminar colonies owing to self-overgrowth; relatively wide and flat autozooids; numerous, small subcircular to trifoliate pseudopores with 2–4 spiny, radial processes unfused centrally, arranged in 1–3 rows between orifice and ascopore and one or two marginal, often complete, rows along the barely visible cryptocystidean rim; 1–3, very distal oral spines; arcuate ovicell lateral lappets overarching lateral sides of orifice.

##### Description.

Colony encrusting, multiserial, lobate, multilaminar owing to self-overgrowth (Fig. 16A, D), ~1 cm^2^ in size.

Autozooids rounded hexagonal or irregularly shaped, distinct, with very narrow, deep grooves marking the boundaries (Fig. 16B–E). Lateral and proximal walls deeply sloping to sub-vertical, only locally exposing their upper parts, mostly at corners. Frontal shield nearly flat with faint dimpled appearance, more marked centrally in slightly elevated ascopore zone. Gymnocyst forming a narrow rim of calcification distal and lateral to orifice. Cryptocystidean area extensive, almost undefined, mirroring autozooidal boundary and proximal and lateral margins of orifice, lining it in non-ovicellate autozooids, slightly diverging laterally in ovicellate ones (Fig. 16E), forming subtriangular latero-oral extensions, longer in non-ovicellate autozooids (110–152 μm long), usually reaching the distal orifice margin. Pseudopores of the frontal shield arranged in a peripheral row of ~20, usually adjacent to frontal edge, more spaced proximally (Figs 16C–F, 23G), with some sparse pseudopores forming an additional discontinuous row. Two, rarely three, additional rows of pseudopores (9–14) between orifice and ascopore. Pseudopores on a level with frontal surface, subcircular to trifoliate, with 2–4 spiny radial processes unjointed centrally (Fig. 16G, H). Two (occasionally 1 or 3) circular to elliptical cryptocystidean areas, lined by an elevated rim, distal to orifice, each with a single pseudopore and numerous spiny processes (Fig. 16D, E–G).

Primary orifice transversely D-shaped, hinge-line straight, with smooth thin rim; proximal and distal rims smooth, with very low shoulders at proximal ends. Two, occasionally one or three, thin and short oral spines (base diameter ~12 μm) distal to orifice (Fig. 16F–I), sometimes four in periancestrular autozooids; two spines laterally and proximally displaced, barely visible in ovicellate zooids (Fig. 16E).

Ascopore placed slightly distal to autozooid centre, at variable distance (84–126 μm) from the orifice (Fig. 16C–H), lumen transversely C-shaped (Fig. 16G, H), wide, with finely denticulated rim, situated in a sub-circular to transversally elliptical field of smooth gymnocyst marked by a smooth raised rim, fusing with arched proximal rim of frontal shield in the presence of an ovicell (Fig. 16D).

Ovicell subglobular, prominent, obscuring the distal part of orifice, with lateral lappets forming an overarching ovate-like structure (Fig. 16C–E), seemingly subcleithral, only partly closed by the operculum, produced by the distal autozooid (Fig. 16B–D). Endooecium well calcified, dimpled centrally, becoming smoother and intumescent peripherally and proximally, ending in a narrow rim folding upward; rimmed by a row of 15 or more marginal pores (12–40 μm wide, occasionally up to 96 μm). Ectooecial margin comprising a very low, thin gymnocystal arc curving across frontal surface of distal autozooid (Fig. 16D).

Ancestrula covered by self-overgrowing colony lobes.

Kenozooids not observed.

##### Etymology.

From the Latin *ovatus*, meaning egg-shaped, referring to the overall shape of the ovicell created by the lateral lappets.

##### Remarks.

*Fenestrulina ovata* sp. nov. resembles *F. juani*, *F. cavernicola* sp. nov., and *F. foveolata* sp. nov. in its ornamented frontal surface, particularly sharing the dimpled appearance of the ovicell surface with the latter. However, it can be easily distinguished from these species. The prominent ovicell ornamentation, the morphology of both the ascopore and the frontal pseudopores, and the presence of bifurcated spines set it apart from *F. juani*. The combination of a spiny ovicell, a large fissure separating the endooecium and ectooecium, and long lateral lappets deeply indented by prominent, large spines distinguishes *F. cavernicola* sp. nov. The prominent, bifurcated spines, even in ovicellate autozooids, are typical of *F. foveolata* sp. nov.

Lateral lappets of the ovicell extending to the proximal border of the orifice are also observed in other species, mostly from the Southern Hemisphere. *Fenestrulina ampla* Canu & Bassler, 1928, from a depth of 120 m off Brazil, has smooth frontal shields and ovicells, with stellate pseudopores commonly found on the proximal part of the autozooids. Similarly, *F. antarctica* has ovicells that extend over the proximal rim of the orifice. However, it lacks lateral lappets, and the ovicell surface is granular unlike *F. ovata* sp. nov. (see also Remarks of *F. granulosa* sp. nov.). *Fenestrulina catastictos* Gordon, 1984, from the Kermadec Ridge, also has proximally extending ovicells, which, however, feature a complex and prominent ornamentation, as well as pseudopores similar to those of the autozooids. This species also has numerous pseudopores, initially open in young autozooids and later occluded, distributed on the entire frontal shield, a distinct ribbon-like cryptocystidean area distal to orifice, and lacks oral spines. *Fenestrulina epiphytica* Hayward & Ryland, 1995, from the Great Barrier Reef, Australia, has large, flat and smooth ovicells reaching the proximal border of the orifice. This species also has autozooids with smooth frontal shields, a row of peripheral pseudopores encircling the orifice also distally, and doubling between the orifice and the ascopore, which is crescentic and smooth-rimmed.

Autozooids in *F. ovata* sp. nov. exhibit considerable size variability and may develop irregular shapes, including proximal cauda-like extensions (Fig. 16B) or pointed proximal corners wedged between adjacent modules (Fig. 16C). Deformed autozooids, including giant forms (Fig. 16D, centre) or those with abnormally large latero-oral lappets extending distally to encircle the orifice (Fig. 16D, arrowed) have been observed. These deformities are similar to those observed in *F. communis* sp. nov. Evidence of regeneration, although rarer than in other species like *F. variorugosa* sp. nov., includes intramural budding with partial reconstruction of the frontal shield (Fig. 16D) or the simple oral rim (Fig. 16E, left ovicellate autozooid). Notably, *F. ovata* sp. nov. demonstrates the ability to self-overgrow forming multilayered colonies, with autozooids elevating above parental ones and laterally overgrowing adjacent zooids (Fig. 16D). This feature, to our knowledge previously unreported in *Fenestrulina* species, may offer an advantage in cave colonisation. Similar to other bryozoans adapted to cryptic and cave habitats, such as *Onychocella marioni* Jullien, 1882 (Harmelin 1985; Rosso et al. 2019b, 2020), this strategy likely aids in maintaining colony space and elevating the living layer into the water flow, thereby enhancing feeding opportunities in food-depleted submarine cave environments.

A colony encrusting a large, broken piece of pottery collected at 23 m depth in the Gulf of Fos by J.-G. Harmelin, remains of uncertain attribution. The dimpled frontal shield resembles that of *F. ovata* sp. nov. or *F. foveolata* sp. nov. but the few preserved spines are not bifurcating unlike *F. foveolata* sp. nov., making the attribution to *F. ovata* more plausible. However, the absence of ovicells and the inability to perform SEM examination, due to the large size of the substrate that cannot be reduced without risking damage to the colony, preclude definitive identification.

##### Habitat distribution.

*Fenestrulina ovata* sp. nov. has so far been found in different habitats and contexts, all characterised by a reduction of light at relatively shallow depths (6–23 m). Colonies seem to be relatively common in submarine caves in completely dark zones (6 m), as well as in cryptic microhabitats in coralligenous concretions (17 m). The schiaphilic preferences of *F. ovata* sp. nov. are also indicated by its occurrence on the underside of a rock in coarse sedimentary bottoms at 23 m depth.

##### Geographical distribution.

*Fenestrulina ovata* sp. nov. has currently been found only in localities in the Gulf of Lion, in the northern sector of the Liguro-Provençal basin.

#### ﻿ Fenestrulina variorugosa

Taxon classificationAnimaliaCheilostomatidaFenestrulinidae

Rosso & Di Martino sp. nov.

9BB7F1AA-3432-5707-8BCB-5C43E1A623E0

https://zoobank.org/2BA6A249-F566-4C88-B7E6-ECAC8BD470AB

Figs 1, 17, 18, 19, 20, 22, 23, 24; Tables 1, 4

Fenestrulina malusii (Audouin): Zabala et al. 1993: list of species; Chimenz Gusso et al. 2014: 166 (pars), fig. 83a, b.

##### Type material.

Italy • ***Holotype*** 1 large ovicellate colony without ancestrula on a rhizome of *Posidonia oceanica* (Linnaeus) Delile. Mediterranean, Sicily Strait, W Sicily, Egadi Islands, Formica Isle, sample EFI 20; 37°59'14"N, 12°25'34"E; 8 m depth; Oct. 2007; scuba diving; A. Sinagra leg.; PMC.B43.23.2.2024.a. Italy • ***Paratype*** 1 juvenile colony including 15 non-ovicellate autozooids around the ancestrula; same details as the holotype; PMC.B43.23.2.2024.b.1.

##### Other material examined.

Italy • 2 living colonies on the shell of a living specimen of *Lithophaga lithophaga* (Linnaeus, 1758). Mediterranean, Italy, Tyrrhenian Sea, NE Sicily, Secca di Levante, Capo Milazzo Peninsula, sample MI_SdL_G; 38°14'43"N, 15°14'26"E; 33 m depth; Coralligenous biocoenosis; 17 May 2024; scuba diving; G. Donato leg.; PMC Rosso-Collection I.H.B.160.a.1a. Italy • 5 living colonies on a soft-bodied alga. Mediterranean, Italy, Tyrrhenian Sea, NE Sicily, Secca di Ponente, Capo Milazzo Peninsula, sample MI_SdPn_G; 38°16'28"N, 15°13'22"E; 33 m depth; Coralligenous biocoenosis; 6 May 2024; scuba diving; G. Donato leg.; PMC Rosso-Collection I.H.B.160.a.1b. France • 1 living colony with only few functional autozooids on a dead coral fragment. Mediterranean, Liguro-Provençal basin, Cassis, Cassidaign Canyon; no coordinates available; 300 m depth; Bathyal Corals biocoenosis; 21 Jun. 1969; J.-G. Harmelin leg.; PMC J-GH-Collection F.H.B.160.b.1. France • 5 living ovicellate and non-ovicellate colonies on a bioconcretion of white corals hosting a few *Crania anomala* (Müller, 1776) specimens and several cryptic arciid and mytilid bivalves, with 1 out of 5 colonies encrusting the outer shell of an arciid. Mediterranean, Liguro-Provençal basin, off Banyuls-sur-Mer, sample 5; 42°4.30'N, 3°25'E; 200–300 m depth; Bathyal Corals biocoenosis; Jun. 1984; J.-G. Harmelin leg.; PMC J-GH Collection F.H.B.160.c. France • 1 living colony encrusting the underside of a stone. Mediterranean, Liguro-Provençal basin, Port Cros Park, Cave of the Bagaud Island; 43°00.9'N, 6°21.6'E; 7 m depth; June 1984; J.-G. Harmelin leg.; PMC J-GH Collection F.H.B.160.d. France • 1 living colony encrusting the inner surface of an empty shell of a dead *Pinna nobilis* (Linnaeus, 1758). Mediterranean, Liguro-Provençal basin, Veyron Plateau, off Marseille; coordinates not available; 24 m depth; 23 Sep. 1983; J.-G. Harmelin leg.; PMC J-GH Collection F.H.B.160.e. Tunisia • 2 colonies. Mediterranean, Sicily Strait, off Tabarka; coordinates not available; 86 m depth; J. Jullien Collection; MNHN_IB_2008_2590.

##### Diagnosis.

*Fenestrulina* with lobate pseudopores characterised by three or four irregularly curving spinules, thickening and flattening towards the centre without meeting, arranged in a single row, some becoming semicircular, leaning against rim of frontal shield; globose ovicell rimmed by several small peripheral pores with variable endooecial ornamentation, ranging from gently nodular, to faintly ribbed and scalloped at the periphery, or prominently rough with radial crests.

##### Description.

**Figure 17.**
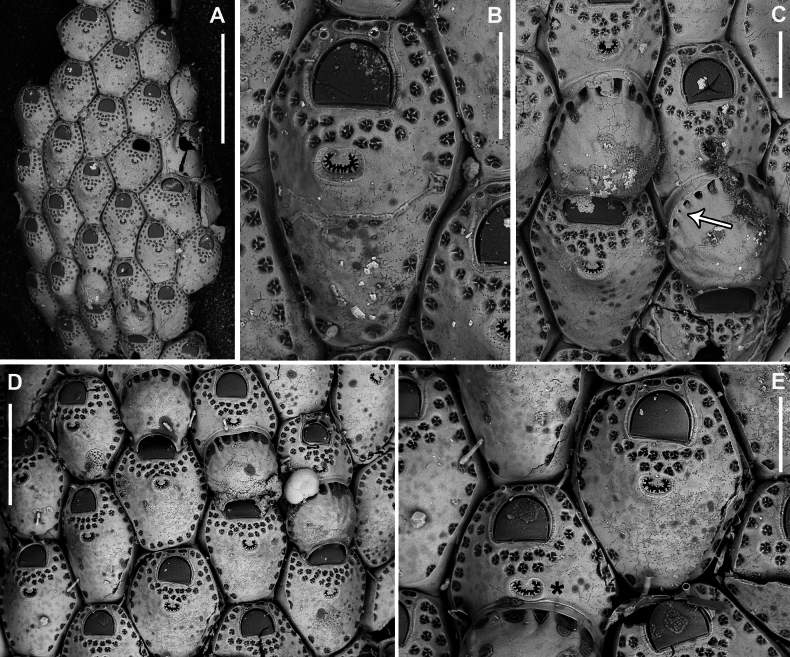
*Fenestrulina variorugosa* Rosso & Di Martino, sp. nov. holotype PMC.B43.23.2.2024.a, Formica Isle, Egadi Islands, W Sicily, Sicily Strait. **A.** Colony portion; **B.** Autozooid with two very distally located oral spines and an elongated fused cryptocystidean area in between; **C.** Slightly distal view of two ovicells showing the peripheral row of pores partly occluded by secondary calcification (arrowed); **D.** Ovicellate and non-ovicellate autozooids; **E.** Two non-ovicellate autozooids, one distal to an ovicell. Note some semicircular to fissure-like pseudopores proximo-laterally. Scale bars: 1 mm (**A**); 200 μm (**B, C, E**); 500 μm (**D**).

**Figure 18.**
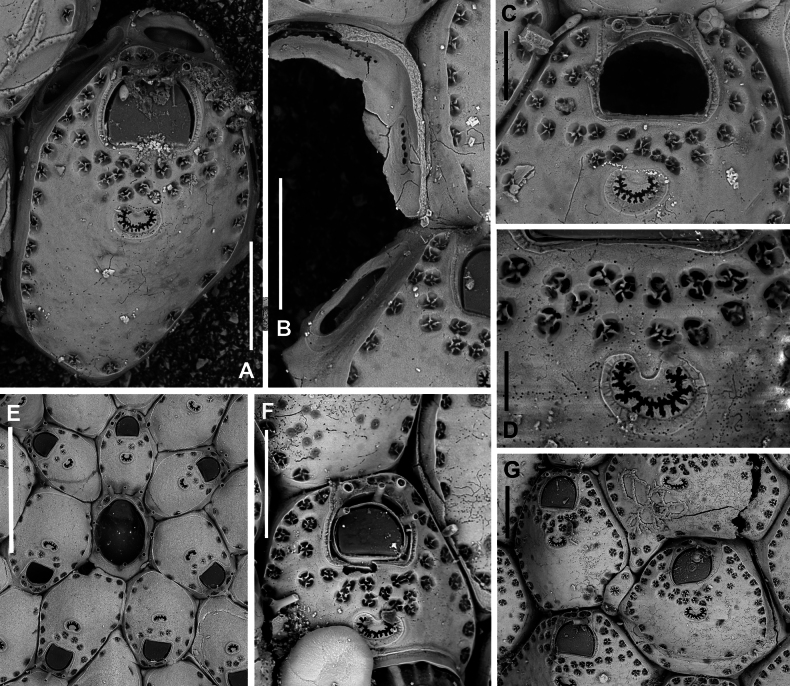
*Fenestrulina variorugosa* Rosso & Di Martino, sp. nov. **A–D, F, G.** Holotype PMC.B43.23.2.2024.a, Formica Isle, Egadi Islands, W Sicily, Sicily Strait; **E.**PMC Harmelin Coll. F.H.B.160.e Veyron Plateau, off Marseille, Liguro-Provençal basin. **A.** Autozooid showing distal and distolateral pore chamber windows; **B.** Internal view of the lateral and distal walls of an autozooid showing multiporous septula; **C.** Distal part of an autozooid. Note the orifice with gently denticulated distal rim and straight proximal margin with two lateral denticles; **D.** Detail of the flower-like pseudopores and the denticulate ascopore; **E.** Tatiform ancestrula and periancestrular autozooids; **F.** Regenerated autozooid with three oral spines; additional spines from the original bud are also visible; **G.** Small kenozooid filling the space between autozooids. Scale bars: 200 μm (**A, B, E–G**); 100 μm (**C**); 50 μm (**D**).

**Figure 19.**
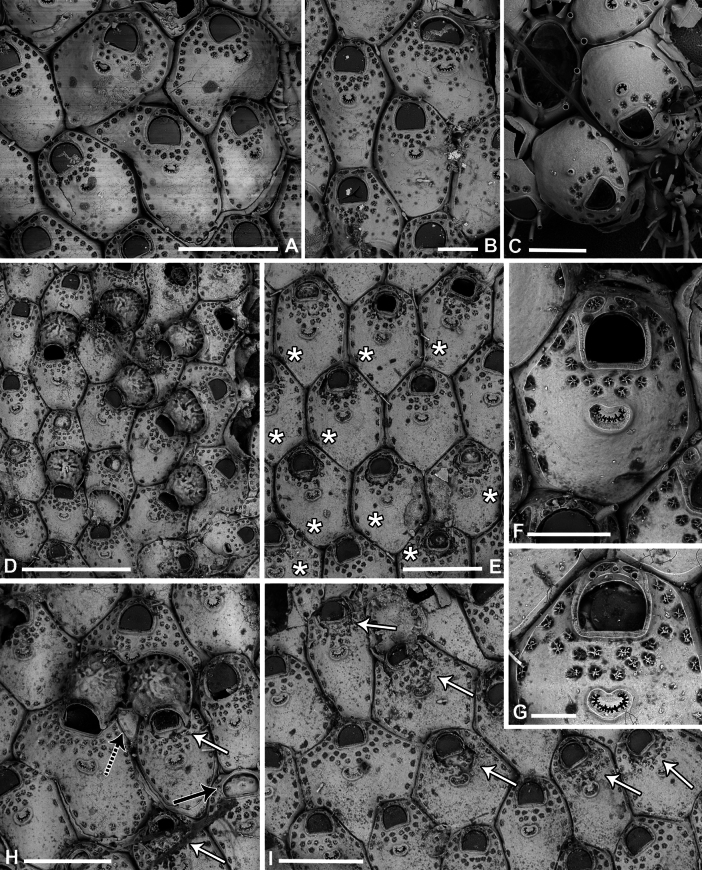
*Fenestrulina variorugosa* Rosso & Di Martino, sp. nov. regenerations and teratologies. **A, B.** Holotype B43.23.2.2024.a Formica Isle, Egadi Islands, W Sicily, Sicily Strait; **C.** Paratype B43.23.2.2024.b, Formica Isle, Egadi Islands, W Sicily, Sicily Strait; **D–I.**PMC Rosso Coll. I.H.B.159a, Secca di Levante, Capo Milazzo Peninsula. **A.** Damaged colony portion with regenerated, irregularly shaped autozooids, leaving spaces between them; **B.** Very large autozooid (upper right corner) with a noticeably wider orifice and operculum, and a deformed ascopore; **C.** Deformed autozooid at the periphery of the young paratype colony, intergrowing with a cribrimorph that is only partially covered; **D.** Group of autozooids, some ovicellate. Note the prominent rugosity, which show a preferential radial pattern; **E.** Group of non-ovicellate autozooids, several (asterisks) showing evidence of regeneration at orifice level; **F, G.** Details of autozooids, orifice, ascopore and pseudopores. Note the distal position of the oral spines; **H.** Deformed, giant autozooids, one developing two ovicells for both autozooids located proximally. Note the regeneration evidence (white arrows), the presence of a small kenozooid (black dashed arrow), and the orificial closure plate (black solid arrow); **I.** Some irregularly shaped autozooids with frequent evidence of regeneration (arrowed). Scale bars: 500 μm (**A, E, H, I**); 200 μm (**B, C, F**); 1 mm (**D**); 100 μm (**G**).

Colony encrusting, multiserial, unilaminar (Fig. 17A), irregularly shaped in relation to the substrate morphology, ~1 mm in diameter. Interzooidal communications via pore-chambers, two proximolateral, two distolateral (130–150 μm), one distal (~120 μm) (Figs 18A, B, E, 19C, F), each with 6–10 round pores, 6–9 μm in diameter (Fig. 18B).

**Figure 20.**
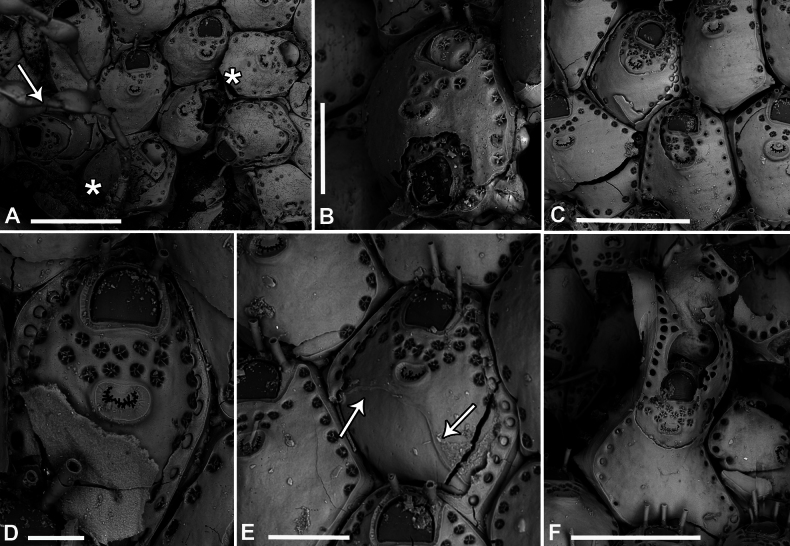
*Fenestrulina variorugosa* Rosso & Di Martino, sp. nov. regenerations, PMC Rosso Coll. I.H.B.160.a, Secca di Ponente, Capo Milazzo Peninsula, NE Sicily, Tyrrhenian Sea. **A.** Damaged zooids with evidence of multiple regenerations (arrowed) and occluded orifices (asterisks); **B.** Regenerated autozooid with opposite polarity and a pseudoporous plate occluding the orifice; **C–E.** Regenerated autozooids with pseudopores in the first frontal shield, hole-shaped and lacking the typical spinous processes, sometimes occluded by secondary gymnocystal calcification. The scar left by the margin of the detached original frontal shield is arrowed in **E**; **F.** Regeneration producing elevated, irregularly shaped autozooids that overarch the previous colony layer. Scale bars: 500 μm (**A, C, F**); 200 μm (**B, E**); 100 μm (**D**).

Autozooids hexagonal, distinct, contiguous, boundaries marked by narrow, deep grooves widening at triple junctions, exposing upper parts of sub-vertical lateral walls (Figs 17B–E, 19A–E). Frontal cryptocystidean area outlined by a thin, slightly raised rim, more pronounced at pseudopore level, lining orifice proximally and laterally, developing long (mean length 148 μm, *n* = 11) lappets on both sides of orifice (Figs 17B–E, 18A, F, G, 19A–C, F, G). These lappets occasionally extending distally and almost encircling the orifice but never joining (Fig. 19A). Two elliptical, occasionally one elongate, cryptocystidean areas distal to the orifice, between oral spines, each with one or two pseudopores, rarely more (Figs 17B–E, 18A, C, F, 19F, G). Frontal surface gently convex, more raised at ascopore level, smooth, perforated by 32–45 pseudopores, 12–20 in periancestrular autozooids (Figs 18E, 19C). Pseudopores mostly located in distal half of autozooid, arranged in two rows between orifice and ascopore, one or two rows in lateral lappets (Figs 17B, E, 18A, C, F), in a single row along lateral zooidal margins, sparse or absent proximally (Figs 17C–E, 19). Pseudopores on a level with frontal shield, irregularly subcircular to slightly lobate, semicircular (Figs 17D, 19) or slit-like (Fig. 17E) along lateral rim; each with three or four (rarely more) spinules projecting, thickening and flattening or branching centrally, never merging (Figs 18C, D, F, 23H). Circular pseudopores without spinules in regenerated autozooids in damaged colony areas (Fig. 20C–F), sometimes occluded by underlying gymnocystal calcification (Fig. 20D, E). Basal wall largely uncalcified.

Primary orifice transversely D-shaped, hinge-line straight with two tiny denticles near proximal corners; distal rim fairly denticulated (Fig. 18C). Two oral spines in most autozooids (Figs 17B–E, 18C, F, G), rarely three (Figs 18G, 19G), four observed in the first periancestrular ones (Fig. 18E), ~100 μm long (base diameter 15–25 μm), located along distal curvature (Figs 17B–E, 18C, G). Intrazooidal regeneration may alter spine count (e.g., Fig. 18F). Only two, barely visible, spines in ovicellate autozooids, lining ovicell margins near proximal rim (Figs 17C, D, 19D, H).

Ascopore ~90 μm proximal to orifice (Figs 17, 18A, C, D, G, 19F, G), within a reniform field of smooth gymnocyst marked by a slightly raised rim, in contact with the arched proximal rim of the frontal shield in the presence of an ovicell (Fig. 17E) or fusing with it (Fig. 19D, H); large transversely C-shaped lumen between the distal wide tongue and the arched proximal border; rim irregularly denticulated including tiny spindle-like spinules and larger, platy to branched denticles (Figs 17B, E, 18C, D, 19F, G).

Ovicell subglobular, prominent, restricted proximally to fit orifice width, slightly obscuring distal part of orifice, not closed by the operculum, produced by the distal autozooid (Figs 17C, D, 19D, H). Endooecium well calcified, gently nodular and faintly ribbed and scalloped to prominently rough with blunt spiny processes and radial crests at periphery but smoother proximally; rimmed by a row of ~15 quadrangular pores, separated by calcified bridges, often reduced in diameter by secondary calcification (Fig. 17C, arrowed); proximal margin with narrow upturned rim just at corners above oral spines. Calcified part of ectooecium consisting of a narrow (~30 μm) elevated rim of gymnocyst lining the row of pores.

Ancestrula tatiform (Figs 18E, 19C, 24F, G), irregularly oval, similar size to first periancestrular autozooids, gymnocyst apparently narrow, largely covered by periancestrular autozooids in examined material, with ten spines: five around orifice (three distal, closely spaced, two more proximally placed, at a greater distance, aligned with proximal margin of operculum), five around proximal half of opesia (widely and regularly spaced). Opesia oval (305 μm long by 220 μm wide) surrounded by a narrow (~15 μm), almost smooth cryptocyst. Two longitudinally elongated cryptocystidean areas (2 or 3 pores each) between distal triad and two more proximal oral spines (Fig. 18E). Budding pattern: one distal, two distolateral, two proximolateral and two proximal autozooids (Fig. 18E).

Kenozooids small, triangular to quadrangular, elongate, filling empty spaces between autozooids in areas without evidence of reparation, including few relatively large pseudopores with 5–7 denticles giving a stellate appearance (Figs 18G, 19H).

##### Etymology.

Referring to the variability of the ovicell ornamentation, especially the variable degree of the endooecial rugosity.

##### Remarks.

*Fenestrulina variorugosa* sp. nov. resembles *F. barrosoi* Álvarez, 1993, an Atlanto-Mediterranean species described from the Alboran Sea at depths of 15–20 and 50–60 m (Álvarez 1993), and later recorded at 112–120 m depth (Ramalho et al. 2022), and from the Galician coast on seagrasses at ~15 m depth (Reverter Gil et al. 2019). Both species share the general morphology of ovicells and autozooids, including the shape and location of the ascopore. However, all morphological measurements, except for the autozooidal length of the holotype, tend to be smaller in *F. barrosoi* than in *F. variorugosa* sp. nov. In *F. barrosoi*, pseudopores are always distributed in a single row along lateral and proximal margins, without any doubling or absence in these areas, as observed in *F. variorugosa* sp. nov. *Fenestrulina barrosoi* typically has 3–6 oral spines, most commonly four or five based on Álvarez (1993: 833), although his fig. 1 on p. 832 shows zooids with three spines, rather than the 2–4 (mostly two), seen in *F. variorugosa* sp. nov. Importantly, the proximal pair of spines in *F. barrosoi* is stout and shifted proximally. Colonies with rugose ovicells resemble *F. kalliste* sp. nov., but the latter species has heavier ornamentation, with more developed spiny processes and a central area with prominent and often transversal crests. The pseudopore number, distribution and morphology differ significantly, and the proximal oral spines in *F. kalliste* sp. nov. are bifurcated. Rough ornamentation of the endooecium is also seen in *F. cavernicola* sp. nov. and *F. juani*, but in these species, the frontal shield is dimpled. In *F. cavernicola* sp. nov., the endooecium is spinier and bordered by a large peripheral fissure with only a few pores, while in *F. juani*, the ornamentation consists of prominent tubercles.

The variability in *F. variorugosa* sp. nov. primarily pertains to the ornamentation of the ovicell endooecium, which ranges from nearly smooth to highly rough. Autozooid size and shape also vary. Although usually elongate hexagonal and arranged in regular alternating rows, repair in some damaged areas may cause changes in size and shape of some autozooids, including the formation of lateral prominences and relatively enlarged orifices (Figs 18E, 19A–C). Orifices also seem to be dimorphic, becoming slightly wider in ovicellate than in non-ovicellate zooids (Figs 17C, D, 19H). In a single case, a deformed autozooid had an orifice as long as (133 μm vs 112–137 μm) but decidedly wider (227 μm vs 144–170 μm) than average (Fig. 19B). Frontal pseudopores are very close to each other and can often fuse. The denticles on the proximal side of the orifice are very inconspicuous and barely recognisable in some colonies.

Regeneration is common in this species, particularly in specimens living on soft-bodied algae from the Capo Milazzo area (Figs 19, 20). In a colony from sample MI_SdL_G, autozooids were mostly damaged at orifice level, with intramurally budded autozooids showing smaller orifices and limited proximal areas of the frontal shield, usually extending distal to the ascopore (Fig. 19E, H, I). Heavier damage, also affecting larger sectors of the autozooidal frontal shield, was observed in a colony from sample MI_SdPn_G (Fig. 20), including multiple regeneration events per autozooid (Fig. 20A, C), regeneration with opposite polarity (Fig. 20B), and the production of oral closure plates (Fig. 20B, C). Detachment of the original frontal shield after regeneration left traces in the regenerated one (Fig. 20E, arrowed). Regeneration in damaged areas produced elevated irregularly shaped autozooids with circular pseudopores lacking spinules, overarching the underlying colony layer (Fig. 20F). Similar non-spinulose pseudopores occur in *F. caseola* Hayward, 1988 originating from Mauritius and later reported from other Indo-Pacific localities including Australia (Hayward 1988; Tilbrook 2006; Bock 2025https://bryozoa.net/cheilostomata/fenestrulinidae/fenestrulina_caseola.html). However, unlike in *F. variorugosa* sp. nov., those pseudopores can be occluded by the complete coalescence of spinous processes.

Colonies figured in Chimenz Gusso et al. (2014) as *F. malusii* resemble *F. variorugosa* sp. nov. in their general appearance, including the size, morphology and location of pseudopores against the frontal cryptocystidean rim. The two distal spines and the slightly ribbed ovicell are also similar. Colonies from a depth of 86 m off Tabarka (Tunisia) are highly likely to correspond to this species, based on stereomicroscope images kindly provided by Dr. P. Lozouet.

##### Habitat distribution.

To date, *Fenestrulina variorugosa* sp. nov. has been collected from a variety of habitats ranging from shaded and plant-rich areas on the shallow shelf to the upper slope, in association with white corals. Our colonies were found on roots of *P. oceanica*, collected in a flat, rocky area predominantly covered by algae from the Infralittoral Algae biocoenosis, surrounded by the *Posidonia* Meadows biocoenosis (CoNISMa 2009), as well as on soft-bodied algae forming the canopy of the Coralligenous biocoenosis. A few colonies were also observed colonising the shell of a *L. lithophaga* specimen still inside its bore-hole in the coralligenous concretion, suggesting that this species can thrive in cryptic habitats. A few colonies were found on dead coral fragments between 200 and 300 m depth in canyons off Spain and France (partly published in Zabala et al. 1993). However, the habitat distribution may be incomplete, as the information regarding the habitats of the specimens examined by Chimenz Gusso et al. (2014) and those from Tabarka (Tunisia) at the MNHN is not available.

##### Geographical distribution.

In addition to its type locality in the Egadi Archipelago (W Sicily), *F. variorugosa* sp. nov. has also been found in the Aeolian Archipelago (SE Tyrrhenian Sea) and in the north-western part of the Liguro-Provençal basin. The species distribution is further expanded when considering the colonies from off Tabarka (Tunisia), extending its occurrence also to the southern part of the Sicily Strait. Most colonies examined by Chimenz Gusso et al. (2014) were collected from several localities in the Tyrrhenian Sea, and subordinately from the Aegean Sea (Turkey), further expanding the known distribution of this species in the Mediterranean.

#### ﻿ Fenestrulina

Taxon classificationAnimaliaCheilostomatidaFenestrulinidae

sp.

EB4ED9E4-E7D5-5D31-9BE1-95094F9D1A46

**Figure 21.**
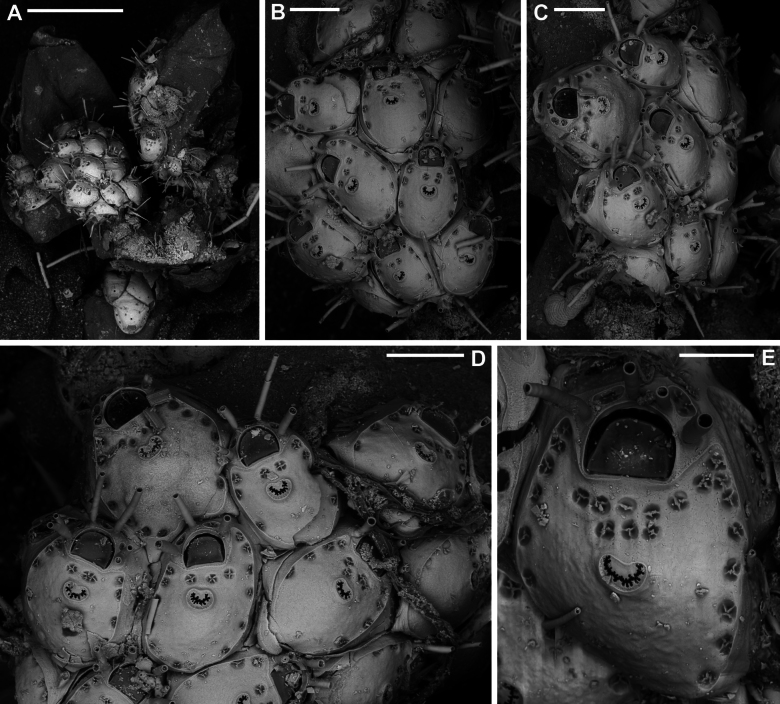
*Fenestrulina* sp., PMC Rosso Coll. I.H.B.159.a, Secca di Ponente, Capo Milazzo Peninsula, NE Sicily, Tyrrhenian Sea. **A.** A possibly lobate colony encrusting a soft-bodied alga. Note the co-occurrence of additional bryozoan colonies, including *Haplopoma* and *Microporella* species; **B, C.** Details of the lobe on the right in panel **A**. Note the long oral spines, with the proximal pair usually bifurcated; **D.** Close-up of some peripheral autozooids; **E.** Close-up of an autozooid showing details of the frontal shield texture, pseudopores, and the ascopore. Note also the unbranched oral spines and the irregularly undulating distal margin of the orifice. Scale bars: 1 mm (**A**); 200 μm (**B–D**); 100 μm (**E**).

**Figure 22.**
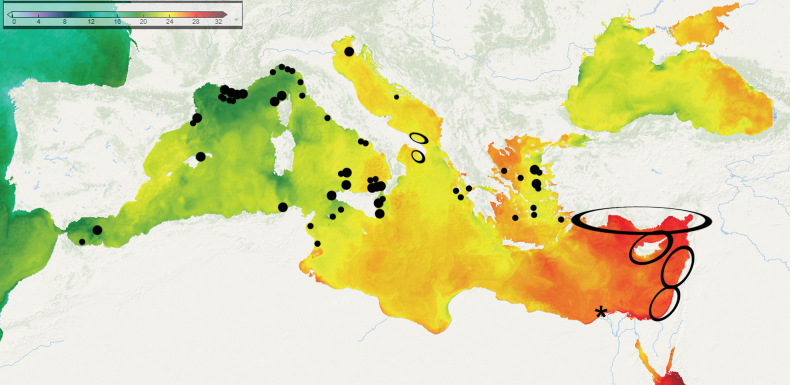
Geographical distribution of all known records of *Fenestrulina* in the Mediterranean, plotted against current summer Sea-Surface Temperatures (data from IFREMER-LOPS Data Visualization Portal https://syntool.ifremer.fr/, accessed Sept. 2024). An asterisk marks the probable origin of the original material of *F. malusii*. Large dots: collection sites of specimens with confirmed species attribution; small dots: records of putative *F. malusii* requiring taxonomic revision, but with known provenance; open ellipses: sites, covered by dedicated monographs, from which *Fenestrulina* species are absent.

**Figure 23.**
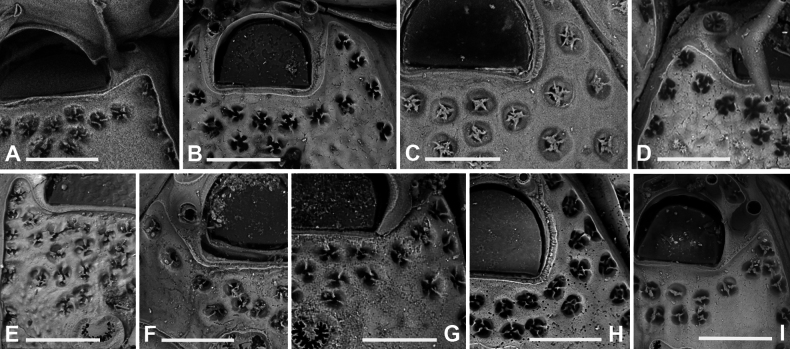
Comparison of pseudopores in the studied species of *Fenestrulina*. **A.***F. malusii*; **B.***F. cavernicola* sp. nov.; **C.***F. communis* sp. nov.; **D.***F. foveolata* sp. nov.; **E.***F. granulosa* sp. nov.; **F.***F. kalliste* sp. nov.; **G.***F. ovata* sp. nov.; **H.***F. variorugosa* sp. nov.; **I.***Fenestrulina* sp. Scale bars: 100 μm.

Figs 1, 21, 22, 23; Tables 1, 4

##### Material examined.

Italy • 1 living colony on a soft-bodied alga. Mediterranean, Tyrrhenian Sea, NE Sicily, Secca di Ponente, Capo Milazzo Peninsula, sample MI_SdPn_G; 38°16'28.2"N, 15°13'22.4"E; 33 m depth; Coralligenous biocoenosis; 6 May 2024; scuba diving; G. Donato leg.; PMC Rosso-Collection I.H.B.159.a.

##### Description.

Colony encrusting, multiserial, unilaminar; interzooidal communications via two proximolateral, two distolateral and one distal pore-chambers, visible externally as elongate, elliptical windows (Fig. 21C).

Autozooids ovoidal, distinct, with grooves in-between (Fig. 21B–D). Upper parts of vertical walls of autozooids gently sloping and exposed, more so at triple junctions (Fig. 21B, D). Frontal shield moderately convex, more elevated at ascopore level. Gymnocyst developed distal and lateral to orifice. Cryptocystidean area marked by a thin raised rim following autozooidal margins and distally lining the proximal margin of the orifice, widely diverging laterally (Fig. 21D, E), forming short blunt subtriangular latero-oral extensions (~24 μm long, not exceeding 58 μm except for a single case, reaching 83 μm). Frontal shield smooth to gently nodular locally. Pseudopores arranged in a single lateral row, usually restricted to distal half of autozooid, occasionally present proximally (Fig. 21B, D). One or occasionally two irregular rows of additional pseudopores (2–8) occurring between orifice and ascopore. Pseudopores on a level with frontal surface, subcircular (rarely elliptical) and slightly infundibular, with 2–4 spiny processes fusing in the centre (Figs 21E, 23I). Two relatively large (25–76 μm wide), subelliptical, smoothly-rimmed cryptocystidean areas occur distolaterally to the orifice, between spines (Fig. 21C–E), each bearing one pseudopore with some spiny processes.

Primary orifice transversely D-shaped, lined by a thin and smooth rim of calcification; hinge-line straight, distal margin fairly undulating (Fig. 21E). Four, occasionally three long (up to ~180 μm), tubular oral spines, ~20 μm in maximum diameter (Fig. 21B–D), the proximal pair bifurcating (Fig. 21B–D) at ~80 μm from the base, with the distal branch being the longer.

Ascopore relatively distal, ~74 μm proximal to orifice (Fig. 21E), the lumen transversely C-shaped, with large distal process and denticulated rim, within a reniform field of smooth gymnocyst, smooth-rimmed, flared and vertically protruding from frontal shield surface (Fig. 21E).

Ovicells, ancestrula, and kenozooids not observed.

##### Remarks.

The presence of very long spines, with the two proximalmost ones bifurcated, combined with a smooth frontal shield featuring only a few relatively large pseudopores having spiny processes joining in the centre, distinguishes this species from all others described here. However, we have opted to leave it in open nomenclature because of the absence of ovicells, pending the discovery of fertile colonies. *Fenestrulina* sp. has been found only once, with a single colony developing small lobes on a green alga, which complicated the identification of ancestrula and periancestrular zooids. The observed lobes are small, and consist of a few autozooids, some of which may represent the zone of astogenetic repetition, but ovicells are absent.

**Figure 24.**
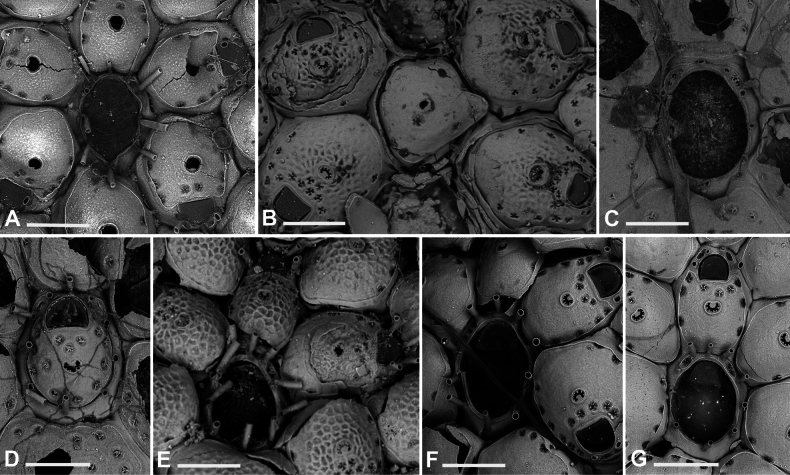
Comparison of ancestrulae in the studied species of *Fenestrulina*. **A.***F. malusii*, tatiform; **B.***F. cavernicola* sp. nov., regenerated as a kenozooid; **C.***F. communis* sp. nov., tatiform; **D.***F. communis* sp. nov., regenerated as a miniature autozooid; **E.***F. foveolata* sp. nov., tatiform; **F, G.***F. variorugosa* sp. nov., tatiform. Scale bars: 200 μm.

##### Habitat distribution.

*Fenestrulina* sp. has only been found in a coralligenous concretions at 33 m depth, on a green alga.

##### Geographical distribution.

*Fenestrulina* sp. has only been found in the Capo Milazzo area, north-eastern coast of Sicily, south-eastern Tyrrhenian Sea.

## ﻿Discussion

All *Fenestrulina* colonies found in the Mediterranean have been identified as *F. malusii* for two hundred years, except for a few colonies described as *F. barrosoi* and *F. juani* from the western part of the basin, with only the latter having a further record (Álvarez 1993; Souto et al. 2010b). Of all this material, good SEM images are available only for *F. juani* (Souto et al. 2010b) and additional SEM documentation is sporadically available in a few recent papers and online, clearly indicating that *F. malusii* is, in fact, a species complex within its proposed restricted Atlanto-Mediterranean distribution range.

The routine use of SEM has revolutionised bryozoan taxonomy by enabling the observation of even the finest morphological details. The application of SEM, often combined with genetic and molecular analyses, along with the modern redescription of historical type material in museum collections (see Di Martino et al. 2022; Di Martino 2022, 2023), has facilitated the dismantling of species complexes for species once considered as cosmopolitan or widely distributed. In the Atlanto-Mediterranean area, notable examples include *Microporella ciliata* (Pallas, 1866), which was separated into five species including the nominal one (Di Martino et al. 2020; Di Martino and Rosso 2021), and *Hemicyclopora multispinata*, which was segregated into at least five species (Harmelin and Rosso 2023). Additionally, the number of *Schizomavella* species has been continuously increasing as original species are re-examined and reclassified (e.g., Reverter-Gil and Fernandez-Pulpeiro 1995; Reverter-Gil et al. 2015a, b; Pica et al. 2022).

In the case of the *F. malusii*-complex, SEM examination of colonies from several localities spanning the entire Mediterranean has led herein to the identification of seven new species: *F. cavernicola* sp. nov., *F. communis* sp. nov., *F. foveolata* sp. nov., *F. granulosa* sp. nov., *F. kalliste* sp. nov., *F. ovata* sp. nov., and *F. variorugosa* sp. nov., significantly increasing the known number of *Fenestrulina* species in the region. Moreover, further species may exist, such as our *Fenestrulina* sp. and the morphotype figured in Gordon (1984: pl. 41F). The presence in the Mediterranean of *Fenestrulina orientalis* Liu, Liu & Sun, 2003, recorded as a non-native species from Cyprus by Galinou-Mitsoudi et al. (2023), is questionable. The stereomicroscope images shown in that paper, along with those kindly provided by S. Galinou-Mitsoudi, depict two or three oral spines, none of which persist in ovicellate autozooids, contrasting with the four oral spines (six in periancestrular autozooids), with two persisting in ovicellate autozooids, originally described by Liu et al. (2003). The ovicell has an unusual pattern of radial ribs seemingly alternating with tubercle alignments instead of the characteristic frontal “fenestra” seen in true *F. orientalis*. Although there is some rough similarity with *F. communis* sp. nov., the inclusion of such material in one of the species described here is hindered by the low definition of images available.

Rosso and Di Martino (2023) listed three *Fenestrulina* species from the Mediterranean Sea, i.e., *F. barrosoi*, *F. juani*, and the repeatedly reported *F. malusii*, considered for a long time the sole representative of the genus in the Mediterranean together with *F. joannae* (Calvet, 1902) now in *Microporella* (Di Martino and Rosso 2021). With the addition of these seven new species, *Fenestrulina* ranks among the eight most speciose Mediterranean genera.

Further studies are needed to fully understand the distribution of these species and to determine whether there are additional undescribed taxa. This would require locating and examining colonies that originated from the numerous records of putative *F. malusii* in the basin (Table 5), other than those already studied here (Fig. 1; Table 1). Contrary to our expectations, and especially considering evidence that drifting plastic can effectively transport encrusting bryozoans over long distances (e.g., Kannan et al. 2023; Rosso et al. 2025), no colony of *F. delicia* has so far been found in the Mediterranean. Since its original description from the western North Atlantic coasts, this species has been reported from widely separated regions, including the north-eastern (De Blauwe et al. 2014; Wasson and De Blauwe 2014) and south-western (López-Gappa and Liuzzi 2016) Atlantic, as well as the north-eastern Pacific (Dick et al. 2005), and is therefore considered highly invasive.

**Table 5.** Compilation of Mediterranean records of putative *Fenestrulina malusii* specimens pending taxonomic revision. The listed sites served as evidence of the genus’ presence in the construction of Fig. 22. Abbreviations for biocoenoses in the notes – CB: Bathyal Corals; DC: Coastal Detritic; DL: Offshore Detritic; GO: Dark Caves; GSO: Semidark Caves; HP: Posidonia Meadows; IA: Infralittoral Algae. Dedicated monographs covering Mediterranean sites where *Fenestrulina* species are absent were also reported.

Sea	Locality	Reference	Notes
Alboran	East of Gibraltar Strait	Harmelin and d’Hondt 1992	Outer shelf rocky bottom (45 m)
	Chella Bank	Ramalho et al. 2020	With coral rubble (250–321 m)
Liguro-Provençal	Balearic Islands, Hyeres, Gran Conclue, canyons along French coast, Napoli, Tunisia	Gautier 1962	Several localities and habitats mostly on the shelf but down to ~300 m
	Rhône delta	Lagaaij and Gautier 1965	Sedimentary bottoms, possibly dead
	Mostly off Marseille, but further localities included	Harmelin 1976	Frequent, mostly associated with Coralligenous, small substrata and detritic bottoms, as well as *Posidonia* meadows and colonisation panels
	Hyères, La Palude, Le Tuf	Harmelin 1977	*Pinna* valves (20–25 m, 35 m)
	Hyères, Port Cros	Harmelin 1978	Detritic bottoms (DC: 40–60 m; DL: 150–200 m)
	Blanes, Lacaze Duthiers, Banyuls	Harmelin et al. 1993	Canyons (CB: 180–350 m)
	3PP cave	Harmelin 1997	GO-GSO habitats
	Cap de Creus	Madurell et al. 2013	Shelf habitats, encrusting shells
Tyrrhenian	Riva Trigoso, Genova	Geraci and Valsuani 1973	Occasional, on colonisation panels (28 m)
	Procchio, Elba Island	Geraci and Cattaneo 1980	*Posidonia* meadows (9 m)
	Tigullio, Ligurian Sea	Balduzzi and Deandreis 1980	On asbestos panels
	Portofino, Ligurian Sea	Pisano and Boyer 1985	On colonisation panels (3–15 m)
	Mitigliano cave, Napoli	Balduzzi et al. 1989	GO-GSO habitats (< 16 m)
	Loano, Ligurian Sea	Balduzzi et al. 1994	Artificial panels (5–36 m)
	Latium coasts	Nicoletti and Chimenz 1995	Rock, ?IA-HP, (10 m)
	Vulcano, Aeolian Archipelago	Nicoletti et al. 1996a	Rock, IA, (15–32 m)
	Vulcano, Aeolian Archipelago	Nicoletti et al. 1996b	Rock, IA, (7–25 m)
	Ustica Island	Chimenz Gusso et al. 1999	Rock, on algae, (10–20 m)
	Elba Island, and Tuscany coast, from Livorno to Grosseto	Bedini et al. 2003	*Posidonia* meadows (5–26 m)
	Tuscany coasts	Balata et al. 2007	*Posidonia* meadows (10 m)
	Calafuria, Livorno, Tuscany	Balata et al. 2009	*Posidonia* meadows (7–25 m)
	Latium coasts, Ustica, Vulcano and Stromboli islands	Chimenz Gusso et al. 2014	Different habitats and substrata (6–88 m)
Sicily Straits	Talbot and Terrible banks, off Sicily	Chimenz and Scaletta 1985	Different algae including *Laminaria* (43 m)
	La Chebba, eastern Tunisia	Mabrouk et al. 2014	*Posidonia* meadows (5 m)
	Near Hammameth, eastern Tunisia	Mabrouk et al. 2016	Infralittoral algae (5 and 10 m)
	Tunisia	Ayari and Taylor 2014	Listed from unspecified localities
Ionian	Ciclopi Islands, Catania	Campisi 1973	Associated with algae (down to ~40 m)
	Ciclopi Islands, Catania	Campisi et al. 1973	Associated with *Cystoseira dubia* (25–45 m)
	Patras	Castritsi-Catharios et al. 1985	No clear information reported
	?Zakynthos, ?Kefalonia, Patras	Castritsi-Catharios et al. 1986	No clear information reported
	Gallipoli, south-western Apulia	Pica et al. 2022	NOT FOUND
Adriatic	Brindisi, eastern Apulia	Chimenz and Faraglia 1995	NOT FOUND
	Rovjni Island	Hayward and McKinney 2002	Rock (10–30 m), sediment (20–61 m)
	Lastovo Island	Novosel et al. 2004	Coralligenous and Semidark cave habitats
Aegean	Karpathos	Harmelin 1969	?Coralligenous-Offshore Rock (?30–130 m)
	Chios	Hayward 1974	*Posidonia* meadows (less than ~50 m)
	Chios	Hayward 1975	*Posidonia* meadows (less than ~50 m)
	Psara Island	Castritsi-Catharios and Ganias 1989	*Posidonia* meadows
	Skiathos, Lesvos, Naxos, Santorini	Ganias 1990	On a wide variety of substrates
	Milos	Morri et al. 1999	Rock, also close to hydrothermal vents (10–30 m)
	Datça, Turkey	Chimenz Gusso et al. 2014	Presumably *Posidonia* meadow (5–7 m)
	Marmara Sea and Turkish Aegean	Koçak and Aydin Onen 2014	Different habitats
	Yenikas, southern Turkey	Nicoletti et al. 1995	NOT FOUND
Levantine	Southern coasts of Turkey	Koçak and Aydin Onen 2014	NOT REPORTED
	Lebanon	Harmelin et al. 2016	NOT FOUND
	Israel	Sokolover et al. 2016	NOT FOUND
	Cyprus	Achilleos et al. 2020	NOT FOUND

Clarifying the status of *F. malusii*, following the designation of a neotype, fully compliant with the qualifying conditions set out in Article 75.3 of ICZN (1999), also has important implications for environmental monitoring and management. Possibly influenced by Hayward’s (1974, 1975) records of this species from Chios, where it was associated with *Posidonia* meadows, *F. malusii* has often been included in reports provided to stakeholders and monitoring agencies as indicative of this habitat (e.g., https://www.rac-spa.org/sites/default/files/doc_medmpanet2/a2posidonie_eng.pdf), and occasionally in Natura 2000 (network of protected areas) protocols and data sheets by the European Environment Agency (e.g., https://www.regione.fvg.it/rafvg/export/sites/default/webletter/agri_for/_41/Allegati/Allegato_2_alla_Delibera_945-2013.pdf or https://download.mase.gov.it/Natura2000/Trasmissione%20CE_dicembre2022/schede_mappe/Friuli/ZSC_schede/Site_IT3330008.pdf). *Fenestrulina malusii* is also mentioned in outreach materials illustrating *Posidonia* meadow habitat functioning (https://spa-rac.org/en/publication/download/1135/ecological-role-of-posidonia-oceanica-meadows-albanian-arabic-croatian-english-french-greek-italian-maltese-montenegrin-turkish). However, none of the colonies examined from *P. oceanica* were found to be conspecific with the designated *F. malusii* neotype. Instead, they were identified as other species, i.e., *F. granulosa* sp. nov. and *F. variorugosa* sp. nov. As a result, corrections are necessary in these official reference lists and monitoring protocols, underscoring the critical importance of accurate taxonomic identification for effective conservation and monitoring efforts.

The literature includes several records of “*F. malusii*” (Fig. 22; Table 5), most of which are from the western Mediterranean, especially the northern Liguro-Provençal basin and the Tyrrhenian Sea, although the exact provenance and status of the colonies, whether alive or dead, at the time of sampling is often unclear. Gautier (1962) reported “*F. malusii*” from several localities along the French coast, mostly near Marseille (Grand Conglue Island, Riou Archipelago, Hyères, Villefranche), and in submarine canyons of the region. It also occurs in deep settings along the French and Catalan Spanish coasts, later reported as associated with deep-water corals (Zabala et al. 1993; Madurell et al. 2013), and in the Alboran Sea (Harmelin and d’Hondt 1992; Ramalho et al. 2020). Dead colonies were also reported from the Rhone delta (Lagaaij and Gautier 1965). Harmelin (1976) reported the species from a large number of stations he analysed in his Tubuliporina monograph, primarily from localities in the Gulf of Lion, including 28 of 33 stations collected in the Coralligenous biocoenosis, 7/13 from *Posidonia* meadows, 29/70 from detritic bottoms (0–300 m: in 15 stations the species was found on small substrata), and 15/43 on colonisation panels deployed in different shallow-water habitats. Further records from the western sector of the Mediterranean include shallow-water bright habitats, such as *Posidonia* meadows where the species was rare on leaves (Calvi, Corsica: Lepoint et al. 2014, 2016), the Infralittoral Algae biocoenosis, on the lower side of *Codium bursa* (Olivi) C. Agardh, between 22 and 35 m depth (French Mediterranean coast: Voigt and Harmelin 1986), inside *Pinna* shells (Harmelin 1977), submarine caves (Harmelin 1997) and deeper habitats (Cadiz, 30–50 m: López de la Cuadra and Garcia Gomez 1988; Hyères and Port-Cros Island, at 40–60 m and 150–200 m, respectively: Harmelin 1978). Colonies have also been reported from drift plastic items off Palamós (Subías-Baratau et al. 2022).

Numerous records are also available from the Ligurian and the northern Tyrrhenian seas, usually from shallow-water settings associated with vegetated bottoms including *Posidonia*, or artificial substrates and colonisation panels (Geraci and Valsuani 1973; Balduzzi and Deandreis 1980; Geraci and Cattaneo 1980; Pisano and Boier 1985; Balduzzi et al. 1994; Bedini et al. 2003; Balata et al. 2007, 2009). There are also numerous records from the southern Tyrrhenian Sea (Gautier 1962; Balduzzi et al. 1989; Nicoletti et al. 1996a, b; Chimenz Gusso et al. 1999, 2014), and the Sicily Strait again often associated with *Posidonia* and algae in shallow settings (Gautier 1962; Chimenz and Scaletta 1985; Ayari and Taylor 2014; Chimenz Gusso et al. 2014; Mabrouk et al. 2014, 2016). In contrast, records are less numerous from the Ionian and the Adriatic seas where “*F. malusii*” has been reported only by Hayward and McKinney (2002) off Rovinj and by Novosel et al. (2004) off Lastovo Island (northern Adriatic Sea), in the Ciclopi Islands (western Ionian Sea) by Campisi (1973) and Campisi et al. (1973), and the Gulf of Patras (eastern Ionian Sea) by Castritsi-Catharios and Ganias (1989) and Castritsi-Catharios et al. (1985, 1986) and possibly also from Zakynthos and Kefalonia islands by these latter authors.

In the eastern Mediterranean, records are concentrated in the Aegean Sea (see Gerovasileiou and Rosso 2016). In addition to those already discussed, they include colonies collected along the eastern Peloponnese and around islands in the northern and central parts of the sea, including Psara, near Chios, Skiathos, Lesvos, Naxos and the southernmost localities at Santorini (Ganias 1990). Further collections were made from around Chios (Hayward 1974) and Milos islands (Morri et al. 1999). In the south-easternmost edge of the Aegean Sea, only a dead colony was reported off Kas, below 100 m by Harmelin (1969). Records also exist for Turkish localities such as Datça (Chimenz Gusso et al. 2014), restricted to the Aegean Sea but absent from south-eastern localities in the Levantine Sea (Koçak and Aydin Önen 2014, 2024).

The truly enormous, and possibly largely unsuccessful, effort that would require to find all this material often lacking repository information, is beyond the scope of this study. However, some of these colonies were examined, aiding characterisation of some of the new species. Notably, it is interesting to observe how *Fenestrulina* records become scarcer from the Ionian Sea, especially the African coast, and are completely absent from the Levantine Sea. While scarcity of records can often be attributed to the lack of local researchers and laboratories (and northern Africa suffers from such limitations), the above records still provide significant insights into the distribution of the genus *Fenestrulina* in the Mediterranean. It is noteworthy that the species is absent in monographs on bryozoans from both western (Gallipoli; Ionian Sea) and eastern Apulia (Brindisi; Adriatic Sea) by Pica et al. (2022: several *Neopycnodonte cochlear* [Poli, 1795] shells from 60 m depth) and Chimenz and Faraglia (1995: 52 stations from waters shallower than 28 m), respectively. It is also absent from localities in the southern Aegean, i.e., located south to Crete and east of Rhodes, although some of these areas were surveyed by the Calypso 1955–1956 (J.-G. Harmelin, pers. comm., 18 Nov. 2024) and Jean Charcôt 1967 (Harmelin 1969) expeditions, as well as from southern Turkey (Koçak and Aydin Önen 2024). No representatives of the genus *Fenestrulina* have been reported in comprehensive bryozoan monographs based on extensive sampling covering different habitats from around Cyprus (Achilleos et al. 2020), and along the coasts of Lebanon (Harmelin et al. 2016) and Israel (Sokolover et al. 2016).

The observed distributional pattern, showing a decline in records from NW to SE localities and a complete absence in some areas, correlates with the map of the Mediterranean Sea Surface Temperature (SST) (Fig. 22), which increases towards east and south. This suggests that *Fenestrulina* species may be negatively impacted by higher temperatures. The preferred SST range reported for putative *F. malusii*, as recorded in OBIS, is 10–15 °C. A significant proportion of *Fenestrulina* species seems to be restricted to polar or cold-temperate regions, with only a few species found in warmer or tropical seas. Consequently, in the near future, a shift in the distribution of *Fenestrulina* species towards the north and west may be expected, driven by the ongoing rise in sea-water temperatures associated with global change.

Despite the high species diversity of the genus and its frequent records in the Mediterranean, colonies of *Fenestrulina* appear to be scarce. An extensive survey of numerous samples (Table 1), conducted by one of us (AR), revealed only a few colonies per sampling site. A notable exception was recorded near Ustica where ~500 colonies of *F. malusii* were found at a single location. This observation is qualitative, and a more rigorous quantitative assessment is needed. Ideally, this should be based on standardised samples collected from similar substrates likely to be colonised by *Fenestrulina* spp.

The description of the new species also brings the global count of *Fenestrulina* species to 77, although some might be displaced into more appropriate genera upon examination of the type material. The attempt by Soule et al. (1995) to revise *Fenestrulina* and allocate some species to their new genus *Fenestruloides* Soule, Soule & Chaney, 1995 obtained no consensus and cannot be supported. These authors proposed the new genus for species lacking a defined rim of the frontal shield and a row of pseudopores distal to the orifice, but having a frontal avicularium (in a single case), ovicells with one or two pores or imperforate (in addition to the peripheral row of pores), ancestrulae of two types, tatiform (sometimes with a porous frontal skeletal wall without aperture), or a small zooid-like ancestrula with a single row of pores. However, most of these traits show a continuum of variation or vary inconsistently between species.

### ﻿Morphology

Examination of all available Mediterranean colonies, together with published descriptions and figures of the type material, revealed previously unreported characters for the genus. This prompted a revision of the generic diagnosis to incorporate features from recently described species that had previously been overlooked or not considered. When Jullien (1888) established the genus *Fenestrulina*, using *F. malusii* as the type species, he provided the following brief diagnosis “Zoécies dont la paroi frontale est perforée sur nombre de points par les origelles. Orifice semicirculaire avec la lèvre inférieure droite et entière. Fenestrulae en croissante à concavité supérieure.” (i.e., “Zooecia with the frontal wall perforated in numerous places by small pores. Orifice semicircular with a straight and entire lower lip. Ascopore crescentic distally concave”). Although most of these characters can be seen in Savigny’s figure, Jullien’s diagnosis is decidedly schematic and insufficient to confidently assign the increasing number of subsequently described species. As a result, the diagnosis of *Fenestrulina* has been continuously refined over time, occasionally appearing tailored to local or targeted species. These refinements have included details about colony morphology (whether encrusting or erect and flustrine), the abundance and distribution of frontal pseudopores, the fine morphology of the semicircular orifice with or without condyles, the presence/absence of oral spines, the ovicell morphology and its structure including endooecium and ectooecium, the nature of autozooidal connections, and always the absence of avicularia (e.g., Gordon 1984; Hayward 1995; Hayward and Ryland 1999). The absence of avicularia has been one of the most obvious differences between *Microporella*, which typically has one or two avicularia, and all known species of *Fenestrulina*, which lack them. The single exception is *F. morrisae* Soule, Soule & Chaney, 1995, where a single avicularium is found on the very proximal frontal shield of an autozooid, which is puzzling (Soule et al. 1995: fig. 57A).

All known *Fenestrulina* species are encrusting, although *F. jocunda* and *F. marioni* Hayward & Ryland, 1990, seem to have a particular association with erect bryozoans, forming encrusting sleeves around them (see Hayward and Ryland 1990), mimicking erect branches. Only *F. mutabilis* (Hastings, 1932) has been described as erect and bilaminar, seemingly the source of the inclusion of “flustrine” colony morphology in the diagnosis of *Fenestrulina* by Gordon (1984). However, it is possible that even this species is merely encrusting, potentially on laminar sponges, as Hastings (1932: 428) noted: “the two layers of the escharan specimens are readily separable and between them is a thin layer of material which contains some spicules and may be a sponge”. Hastings (1932) also described rootlet chambers for *F. mutabilis*, indicating the potential occurrence of rhizooids, and such structures have been reported for a further epibiont species, *F. commensalis*, growing on a cerianthid (Vieira and Stampar 2014), and for *F. thyreophora* (Gordon 1984).

Pseudopores, usually described as complex in the genus (Hayward and Ryland 1990), show significant variability among species (Fig. 23). They can be simple cylindrical openings (e.g., in *F. proxima* Waters, 1904, as depicted by Hayward and Ryland 1990) maintaining a consistent diameter throughout the thickness of the wall, or infundibular openings with a variable number of radial processes extending from the margins towards the centre without joining (such as in *F. kalliste* sp. nov.). Radial processes can thicken towards the centre and fuse, forming a stellate pattern with four or five points (such as in *F. communis* sp. nov.), or with additional points, often leaving a small, round hole in the middle (e.g., *F. disjuncta* and *F. vivianii* Moyano, 1991). Larger occluding plates with several points (e.g., *F. constellata* Winston, Vieira & Woollacott, 2014) or dentate margins can also develop. These radial processes or star-like occlusions can be placed on a level with the frontal walls or deeper, as it is often the case when more complex cribrate plates develop (e.g., *F. elevora* Florence, Hayward & Gibbons, 2007 but not *F. thyreophora*). In some other species, the pseudopores have a tri- to quadrilobate margin and the same number of radial processes simulating a small flower (such as in *F. variorugosa* sp. nov.), appearing more regular and with a central hole in *F. commensalis* or with more numerous radii in *F. candida* (MacGillivray, 1860, as reported in bryozoa.net), and complex multiple denticulate holes for each of the mostly seven radii in *F. multiflorum* Figuerola, Gordon & Cristobo (2018: 234, fig. 15).

Pseudopores occur within an interior-walled cryptocystidean area delimited by a more or less obvious raised rim outlining a sort of frontal scutum that has been reported only for a few species (e.g., Busk 1857; Gordon 1984; Hayward and Ryland 1995). In several species, the cryptocystidean area is extended to most of the entire frontal surface up to the proximal margin of the orifice, from which two short-to-long lappets extend lateral to the orifice but not distally; additional small roundish to elliptical cryptocystidean areas can develop between oral spines or distally to the orifice. In a few species, however, latero-oral cryptocystidean lappets can meet beyond the orifice (as in *F. commensalis*, see Vieira and Stampar 2014) or fuse, and the orifice becomes surrounded by one or two rows of distal pseudopores, such as in *F. constellata* (Winston et al. 2014).

Like pseudopores, the ascopore lumen also varies from roundish to elongate, though usually transversely C-shaped, with a smooth to finely or markedly and irregularly denticulated rim, located in a roundish/elliptical to reniform ascopore field. The ascopore is usually located in the central and most convex part of the autozooid, being almost equidistant from the proximal margin of the autozooid and that of the orifice. The shape of the ascopore field ranges from circular, as in *F. pauciporosa* Winston & Jackson (2021: 179, fig. 98), to transversely elliptical or reniform outlined by a variably elevated rim, assuming a proximally cup-shaped form in certain species (e.g., *F. catastictos* Gordon, 1984 and especially *F. juani*). An umbo can develop proximal to the ascopore, very small and pointed such as in *F. delicia*, *F. elevora*, *F. incompta* Gordon, 1984, and *F. incusa* Hayward & Ryland, 1990, being often sporadic. More constant and prominent umbones occur in *F. littoralis* Gordon, 2009, *F. proxima* (Waters, 1904), *F. umbonata* O’Donoghue & O’Donoghue, 1926, and *F. blaggae* Soule, Soule & Chaney, 1995. Referring to the latter species, Soule et al. (1995: 169) suggested that the development of an umbo was “stimulated by some type of predator activity”.

Connections between autozooids have been reported alternatively as being facilitated, at least for some species, by basal (e.g., Hayward 1980; Hayward and Ryland 1999) or lateral pore chambers (Hastings 1932; Gordon 1989; Hayward and Ryland 1990), which Gordon (1971) considered to be potential independent heteromorphs. In our material, the string of intercommunicating pores on the internal side and the wide fenestrae on the external side of the lateral walls have been observed in more heavily calcified species, particularly in *F. variorugosa* sp. nov. (Fig. 18B) and *F. communis* sp. nov. (Fig. 8I, J), but not in *F. malusii*, which exhibits smaller fenestrae (Fig. 6B–E), often becoming fissure-like (Fig. 4F), near the base of the lateral walls.

Ovicells are typically described as being produced by the autozooid distal to the maternal one, except for the only observed colony of *F. kalliste* sp. nov. in which two ovicells were produced by kenozooids (Fig. 15H–J). The co-occurrence of zooidal and kenozooidal ovicells within the same genus, and even in the same species, has been previously documented in *Microporella appendiculata* (Heller, 1867) and in *Figularia figularis* (Johnston, 1847) by Di Martino and Rosso (2021) and Rosso et al. (2021). Ovicells are characterised by a completely calcified endooecium surrounded by an almost entirely membranous ectooecium, which forms only a peripheral rim of calcification, leaving an arched fissure crossed by several buttresses or bridges of calcification outlining a row of variably sized marginal pores. However, ovicells in certain species, especially in *F. personata* (MacGillivray, 1883) and *F. jocunda* seem to differ significantly from the common morphology described above, both being entirely perforated. Their placement within the genus should be re-evaluated.

The ancestrula is unknown for some species. When observed, it has mostly been described and/or figured as tatiform (Nielsen 1981), with a large opesia encircled by a crown of usually nine or ten gymnocystal spines [e.g., Gordon (1989) for *F. reticulata* Powell, 1967; Moyano (1983) for *F. microstoma*; Waters (1904) for *F. parvipora* Waters, 1904], but rarely up to 12 (see Soule et al. 1995 for *F. farnswarthi* Soule, Soule & Chaney, 1995 and Liu et al. 2003 for *F. orientalis* Liu, Liu & Sun, 2003). In all Mediterranean species, pending its observation in *F. kalliste* sp. nov., the ancestrula is tatiform (Fig. 24) but it often regenerates as a miniature autozooid in *F. malusii* (Figs 3C, 5D) and *F. communis* sp. nov. (Fig. 9F, G), and as a kenozooid in *F. cavernicola* sp. nov. (Fig. 7D). This latter case seems to be the only observed instance, in addition to the one reported for *F. morrisae* (Soule et al. 1995), of an ancestrula with a porous frontal wall without an orifice. Ancestrulae regenerated as autozooids have been reported or figured for several species, including *F. morrisae* (Soule et al. 1995). These have sometimes been described as similar to subsequent autozooids, but possibly resulting from the regeneration of a tatiform ancestrula. The putative ancestrula resembling a normal autozooid is possibly the first budded zooid in *F. miramara* (Soule et al. 1995: fig. 62a), and in *F. sinica* (Liu et al. 2003: pl. 4, fig. 2). However, despite some possible contrasting interpretations, some species have ancestrulae that are genuine miniaturised autozooids. This is the case for the ancestrula of *Fenestrulina* sp. imaged from Safaga Bay (Ostrovsky et al. 2024, https://bryozoancollection.univie.ac.at/Sammlung/Bryozoa/Safaga_Bay/Cheilostomata/Microporellidae/Fenestrulina/Fenestrulina_sp.html), and those described and illustrated for *F. blaggae* by Dick et al. (2005: fig. 23F) and *F. thyreophora* by Gordon (1989: 61). *Fenestrulina parviporus* Dick & Grischenko, 2016, also has an ancestrula consisting of a miniaturised autozooid (Dick and Grischenko 2016: fig. 28D). All ancestrulae described in the literature for the Mediterranean species show two typical cryptocystidean areas lateral to the orifice, each including one or two pseudopores similar to those of the frontal shield. This character, which has never been described for other genera, could also be used to distinguish isolated ancestrulae, for instance in fouling communities. Interestingly, recently settled ancestrulae show an extensive gymnocyst, while the budding of subsequent autozooids implies a resorption process (e.g., Fig. 9A–C). This event appears to be consistent across all species within the genus, as noted in observations of available material and published photographs.

Kenozooids are described here for the first time in three of the seven Mediterranean species. While present in *F. variorugosa* sp. nov. (Figs 18G, 19H), they are extremely common in *F. communis* sp. nov. (Figs 10K, L, 11H, 12F), showing varying sizes and shapes, including or lacking an ascopore. Ovicell-producing kenozooids have been documented in *F. kalliste* sp. nov. (Fig. 15H–J). Though not explicitly reported, kenozooids can be spotted in some published figures of certain species, such as *F. blaggae* (Soule et al. 1995: fig. 60a), appearing both with and without an ascopore, possibly filling the space between two colonies or colony lobes, as well as in *F. juani* (Souto et al. 2010b: fig. 16), and apparently in *F. irregularis*Maplestone (1909: fig. 10). The “irregular growth with moderate sized pores in the calcareous wall” reported by Waters (1904) in *F. proxima*, filling up the interspaces between autozooids, possibly corresponds to kenozooids as well.

Based on the observations reported above, we propose the following revised diagnosis for the genus *Fenestrulina*: Colony encrusting unilaminar, occasionally becoming multilaminar owing to self-overgrowth. Autozooids with a scutiform cryptocystidean part of the frontal shield delimited by the slightly more elevated surrounding gymnocyst, forming latero-oral extensions (lappets) that in some species can extend, and eventually merge, distal to the orifice, but in most species small circular to elliptical interior-walled areas occur distally. Interior-walled cryptocystidean part of frontal shield entirely porous or with an imperforate central or proximal area, with pores restricted to the lateral margins. Pseudopores complex, with radial processes or occluding plates, usually stellate but sometimes cribrate, flower-shaped or even more convoluted, or composite, placed on a level with the frontal surface or deeper. Ascopore field distinct at a certain distance from the orifice, rounded or kidney-shaped with a round to elongate or C-shaped lumen, the margins smooth or finely to irregularly denticulated. Primary orifice semicircular, with straight proximal edge, sometimes with inconspicuous condyles. Oral spines absent or present, the proximalmost pair usually persisting in ovicellate zooids. Ovicell prominent, hyperstomial, produced by the distal autozooid or a kenozooid, cleithral or acleithral; endooecium calcified and imperforate, except for a basal ring of pores, the surface smooth or rugose and variably ornamented with crests, ridges and tubercles; ectooecium largely membranous, forming a variably developed calcified peripheral rim, characteristically produced as short buttresses between endooecial pores. Interzooidal communication via pore-chamber windows. Ancestrula usually tatiform with 9–12 spines, often regenerated as a miniature zooid or kenozooid, rarely resembling later autozooids. Kenozooids occurring in some species, the frontal surface pseudoporous, with or without an ascopore. Rhizooids occurring in a few species. Avicularia absent.

## Supplementary Material

XML Treatment for Fenestrulina

XML Treatment for Fenestrulina malusii

XML Treatment for Fenestrulina cavernicola

XML Treatment for Fenestrulina communis

XML Treatment for Fenestrulina foveolata

XML Treatment for Fenestrulina granulosa

XML Treatment for Fenestrulina kalliste

XML Treatment for Fenestrulina ovata

XML Treatment for Fenestrulina variorugosa

XML Treatment for Fenestrulina

## ﻿Additional information

### Use of AI

No use of AI was reported.

### Author ORCIDs

Antonietta Rosso https://orcid.org/0000-0001-5565-9513

Emanuela Di Martino https://orcid.org/0000-0002-3892-4036

Gemma Donato https://orcid.org/0000-0003-1783-4688

Blanca Figuerola https://orcid.org/0000-0003-4731-9337

Vasilis Gerovasileiou https://orcid.org/0000-0002-9143-7480

Chiara Siddiolo https://orcid.org/0000-0003-1871-5955

Rossana Sanfilippo https://orcid.org/0000-0001-6154-8027

Francesco Sciuto https://orcid.org/0000-0003-0306-9399

### Data availability

All of the data that support the findings of this study are available in the main text.
